# Reactivation of Epstein–Barr virus by a dual-responsive fluorescent EBNA1-targeting agent with Zn^2+^-chelating function

**DOI:** 10.1073/pnas.1915372116

**Published:** 2019-12-10

**Authors:** Lijun Jiang, Hong Lok Lung, Tao Huang, Rongfeng Lan, Shuai Zha, Lai Sheung Chan, Waygen Thor, Tik-Hung Tsoi, Ho-Fai Chau, Cecilia Boreström, Steven L. Cobb, Sai Wah Tsao, Zhao-Xiang Bian, Ga-Lai Law, Wing-Tak Wong, William Chi-Shing Tai, Wai Yin Chau, Yujun Du, Lucas Hao Xi Tang, Alan Kwok Shing Chiang, Jaap M. Middeldorp, Kwok-Wai Lo, Nai Ki Mak, Nicholas J. Long, Ka-Leung Wong

**Affiliations:** ^a^Department of Chemistry, Hong Kong Baptist University, Kowloon Tong, Kowloon, Hong Kong;; ^b^Department of Chemistry, Imperial College London, London W12 0BZ, United Kingdom;; ^c^Department of Biology, Hong Kong Baptist University, Kowloon Tong, Kowloon, Hong Kong;; ^d^School of Chinese Medicine, Hong Kong Baptist University, Kowloon Tong, Kowloon, Hong Kong;; ^e^Department of Cell Biology & Medical Genetics, Shenzhen University Health Science Center, 518071 Shenzhen, China;; ^f^Department of Applied Biology and Chemical Technology, The Hong Kong Polytechnic University, Hung Hom, Hong Kong;; ^g^Laboratory for Clinical Chemistry, Sahlgrenska University Hospital, SE-41345 Göteborg, Sweden;; ^h^Department of Chemistry, Durham University, Durham DH1 3LE, United Kingdom;; ^i^Department of Biomedical Sciences, The University of Hong Kong, Pokfulam, Hong Kong;; ^j^Department of Paediatrics and Adolescent Medicine, The University of Hong Kong, Pokfulam, Hong Kong;; ^k^Department of Pathology, VU University Medical Center, 1081HV Amsterdam, The Netherlands;; ^l^Department of Anatomical and Cellular Pathology, State Key Laboratory of Oncology in South China, Prince of Wales Hospital, The Chinese University of Hong Kong, Shatin, NT, Hong Kong

**Keywords:** EBV-specific lytic inducer, EBNA1-targeting agent, dual-responsive fluorescent EBV probe

## Abstract

Epstein–Barr nuclear antigen 1 (EBNA1) plays a vital role in the maintenance of the viral genome and is the only viral protein expressed in nearly all forms of Epstein–Barr virus (EBV) latency and EBV-associated diseases, including numerous cancer types. To our knowledge, no specific agent against EBV genes or proteins has been established to target EBV lytic reactivation. Here we report an EBNA1- and Zn^2+^-responsive probe (ZRL_5_P_4_) which alone could reactivate the EBV lytic cycle through specific disruption of EBNA1. We have utilized the Zn^2+^ chelator to further interfere with the higher order of EBNA1 self-association. The bioprobe ZRL_5_P_4_ can respond independently to its interactions with Zn^2+^ and EBNA1 with different fluorescence changes. It can selectively enter the nuclei of EBV-positive cells and disrupt the oligomerization and *oriP*-enhanced transactivation of EBNA1. ZRL_5_P_4_ can also specifically enhance Dicer1 and PML expression, molecular events which had been reported to occur after the depletion of EBNA1 expression. Importantly, we found that treatment with ZRL_5_P_4_ alone could reactivate EBV lytic induction by expressing the early and late EBV lytic genes/proteins. Lytic induction is likely mediated by disruption of EBNA1 oligomerization and the subsequent change of Dicer1 expression. Our probe ZRL_5_P_4_ is an EBV protein-specific agent that potently reactivates EBV from latency, leading to the shrinkage of EBV-positive tumors, and our study also suggests the association of EBNA1 oligomerization with the maintenance of EBV latency.

## Significance

EBNA1 is the only Epstein–Barr virus (EBV) latent protein responsible for viral genome maintenance and is expressed in all EBV-infected cells. Zn^2+^ is essential for oligomerization of the functional EBNA1. We constructed an EBNA1 binding peptide with a Zn^2+^ chelator to create an EBNA1-specific inhibitor (ZRL_5_P_4_). ZRL_5_P_4_ by itself is sufficient to reactivate EBV from its latent infection. ZRL_5_P_4_ is able to emit unique responsive fluorescent signals once it binds with EBNA1 and a Zn^2+^ ion. ZRL_5_P_4_ can selectively disrupt the EBNA1 oligomerization and cause nasopharyngeal carcinoma (NPC) tumor shrinkage, possibly due to EBV lytic induction. Dicer1 seems essential for this lytic reactivation. As can been seen, EBNA1 is likely to maintain NPC cell survival by suppressing viral reactivation.

The Epstein–Barr virus (EBV) is a human herpesvirus which infects the vast majority (>90%) of humans worldwide and can establish life-long persistence in the host. This virus is causatively associated with the development and progression of many human malignancies of lymphocyte and epithelial origin, including Burkitt’s lymphoma, Hodgkin’s diseases, gastric carcinoma, and nasopharyngeal carcinoma (NPC) (1). In EBV-infected tumor cells, viral gene expression is limited to only a few latency-associated proteins which actively contribute to tumor cell growth, apoptosis resistance, and immune evasion (2). Under certain circumstances, the latent virus can be reactivated into its productive lytic phase after induction of immediate early and early phases, which will eventually result in the synthesis of new viral DNA, late structural proteins, and secretion of mature infectious virions with concomitant cell death.

Epstein–Barr nuclear antigen 1 (EBNA1) is the only viral protein expressed in nearly all forms of EBV latency and its associated cancers which plays a vital role in the maintenance of viral genome (3). EBNA1 is also important for the transcriptional activation of some other EBV latency genes (4). Homodimerization of EBNA1 is known to be critical for EBNA1–DNA binding and the subsequent functions of *oriP* (latent origin of replication), including viral DNA replication and segregation, maintenance of the EBV episomal genome, and transcriptional activation (5). Thus, EBNA1 not only serves as a potential marker for clinical imaging but also emerges as a molecular target for the treatment of conditions associated with EBV. Specific inhibition of EBNA1 by dominant-negative EBNA1 mutants (6), antisense oligonucleotides (7), *oriP* blocking agents, and small molecules/macromolecules (8–12) is shown to inhibit tumor cell growth. Furthermore, our recent study shows that the EBNA1-binding peptide **P**_**4**_ derived from the EBNA1 dimeric interface is able to interfere with the homodimerization of the EBNA1 monomer and suppress EBV-infected cell growth (13–16).

**Fig. 1.**
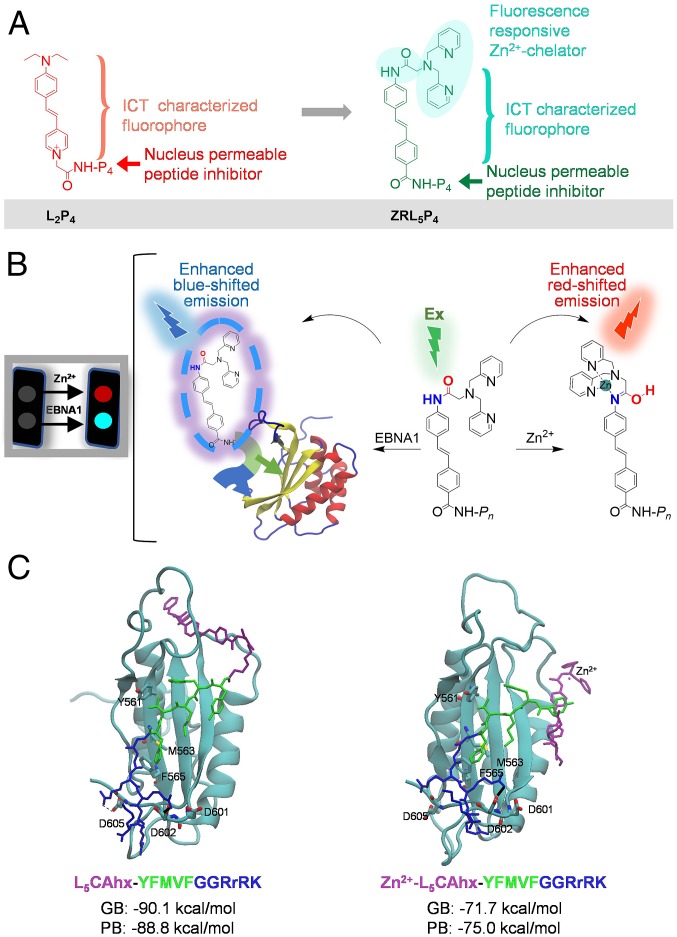
Chemical, fluorescent, and EBNA1-binding characteristics of the Zn^2+^ chelator EBNA1 probe **ZRL**_**5**_**P**_**4**_. (*A*) Chemical structures of **L**_**2**_**P**_**4**_ and **ZRL**_**5**_**P**_**4**_. (*B*) Schematic illustration of the emission response of **ZRL**_**5**_**P**_**4**_, binding to Zn^2+^ and EBNA1. (*C*) Representative conformations of **ZRL**_**5**_**P**_**4**_ and Zn^2+^-**ZRL**_**5**_**P**_**4**_ in the MD simulations. The calculated generalized Born (GB) and Poisson–Boltzmann (PB) values represent the binding free energy between EBNA1 and **ZRL**_**5**_**P**_**4**_ or Zn^2+^-**ZRL**_**5**_**P**_**4**_.

To further improve the activity of the previous peptide-based EBNA1-targeting probe **L**_**2**_**P**_**4**_, we have utilized the EBNA1 cofactor Zn^2+^ and constructed a dual-responsive fluorescent probe, **ZRL**_**5**_**P**_**4**_ (Fig. 1*A*), as a specific imaging and potent anticancer agent for EBV-associated malignancies. As previous studies have shown, Zn^2+^ is necessary for EBNA1 to dimerize and activate the *oriP*-enhanced transcription, and the unique region 1 (UR1) of EBNA1 contains a pair of essential cysteines which serve as donors to chelate the Zn^2+^ ion. This suggests that EBNA1 contains a second dimerization/oligomerization interface in its amino terminus, besides the one located within the DNA-binding domain (DBD) (4, 5). We therefore incorporated 1) an EBNA1 DBD-binding peptide **P**_**4**_ (14), 2) a Zn^2+^ chelator [amide-linked di-(2-picolyl)amine, DPA] to chelate Zn^2+^ adjacent to the EBNA1 protein, and 3) a dual-responsive fluorophore (**ZRL**_**5**_) independently reflecting its binding with Zn^2+^ and EBNA1 to construct a probe, **ZRL**_**5**_**P**_**4**_ (Fig. 1*A*). **P**_**4**_ (YFMVF-GG-RrRK) contains the pentapeptide **P**_**2**_ (YFMVF), which can occupy the first EBNA1 dimerization interface within the DBD (13, 17), and the nuclear localization sequence (NLS) tetrapeptide-RrRK (14). This NLS sequence can form salt bridges with the adjacent dimerization interface, which further enhances the interaction (14). The chosen Zn^2+^ chelator DPA upon binding with Zn^2+^, exhibits enhanced red-shifted emission of the probe (18). In addition, when the peptide **P**_**4**_ binds to EBNA1, the intramolecular charge transfer (ICT)-enabled fluorophore produces enhanced blue-shifted emission. Thus, the probe **ZRL**_**5**_**P**_**4**_ is able to emit 2 independent responsive emission signals when bound to a Zn^2+^ ion and the EBNA1 protein (Fig. 1*B*).

Here we report how the EBNA1 probe **ZRL**_**5**_**P**_**4**_ can interfere the EBNA1 functions and inhibit EBV-positive cell and tumor growth. Indeed, we found that Zn^2+^ is essential for oligomerization but not for dimerization of the full-length EBNA1 protein, and this higher form of EBNA1 structure was specifically inhibited by **ZRL**_**5**_**P**_**4**_, but not by the old **L**_**2**_**P**_**4**_ probe. We observed further enhanced inhibition of *oriP*-mediated transactivation of EBNA1, as well as suppression of growth in EBV-positive tumor cells by **ZRL**_**5**_**P**_**4**_. Surprisingly, we also observed the induction of EBV lytic gene expression and the production of infectious EBV particles when examining the mechanism of how **ZRL**_**5**_**P**_**4**_ could inhibit tumor growth. These interesting results suggest that **ZRL**_**5**_**P**_**4**_ can reactivate EBV from its latent phase through the disruption of EBNA1. The strategy of targeted reactivation of the latent viral genome and the induction of cytotoxic effects in virus-driven tumors is known as cytolytic virus activation therapy, and some EBV lytic inducers have recently entered phase I/II clinical trials (2). Our dual-responsive fluorescent EBNA1 probe **ZRL**_**5**_**P**_**4**_ represents a specific agent to disrupt the EBNA1 protein and to potently reactivate EBV from latency, leading to tumor cell lysis and/or induction of viral proteins that can be targeted by immune cells and antiviral agents to eliminate EBV-infected tumor cells.

## Results

### Molecular Dynamics Simulations of Interactions between ZRL_5_P_4_ and EBNA1.

Before the actual synthesis, interactions between **ZRL**_**5**_**P**_**4**_ (with or without Zn^2+^) and EBNA1 DBD were initially determined by molecular dynamics (MD) simulations. As only the X-ray crystal structure of the dimeric EBNA1 DBD is available, we decided to use this partial structure of EBNA1 to check whether the presence of the Zn^2+^-chelating fluorophore **ZRL**_**5**_ (with or without binding to Zn^2+^) in **ZRL**_**5**_**P**_**4**_ would affect its **P**_**4**_ to bind to an EBNA1 monomer.

Our previous **P**_**4**_-EBNA1 complex model (14) was included as a starting point for a 500-ns simulation by using the flexible peptide docking tool CABS (*SI Appendix*, Figs. S1 and S2) (19). In line with our previous analysis (14), the pentapeptide YFMVF in **P**_**4**_ had occupied the dimerization interface through a hydrophobic interaction, and the NLS tetrapeptide-RrRK formed salt bridges with the adjacent negatively charged residues (D601, D602, and D605), which further enhanced the interaction. Next, 3-dimensional models of **ZRL**_**5**_ in both Zn^2+^-free and Zn^2+^-coordinated scenarios were built and optimized with the molecular mechanics analysis and conformational searching tool LowModeMD. The Zn^2+^-coordinated DPA chelator fragment in the Zn^2+^-**ZRL**_**5**_ molecule superposed very well with the crystal structure of a highly similar compound, Zn^2+^-ZTF (*SI Appendix*, Fig. S3) (18), suggesting that these 2 configurations converged at the global energy minimum. The various scenarios of Zn^2+^-**ZRL**_**5**_ dissolved in different solvents were also taken into consideration, and the Zn^2+^ ion formed 5 coordination sites where a solvent molecule occupies an additional site in DMSO (dimethyl sulfoxide)-Zn^2+^-**ZRL**_**5**_ and H_2_O-Zn^2+^-**ZRL**_**5**_ (*SI Appendix*, Fig. S4).

The tertiary interaction models of the Zn^2+^-unbound and -bound **ZRL**_**5**_**P**_**4**_ with EBNA1 (**ZRL**_**5**_**P**_**4**_-EBNA1 and Zn^2+^-**ZRL**_**5**_**P**_**4**_-EBNA1 complexes) were constructed based on the aforementioned **P**_**4**_-EBNA1 DBD and **ZRL**_**5**_/Zn^2+^-**ZRL**_**5**_ models (*SI Appendix*, Fig. S5). The 2 models were then subjected to 200-ns MD simulations for structure optimization and binding energy calculations (*SI Appendix*, Figs. S6–S9 and Tables S1 and S2). The final optimized models are shown in Fig. 1*C* (simulation 1); both complexes were simulated twice, and the second simulation model is shown in *SI Appendix*, Figs. S7, S9, and S10. Both interaction models showed stable probe–protein interactions. The hydrophobic interactions mediated by YFMVF and the salt bridges formed by RrRK were well conserved in the **ZRL**_**5**_**P**_**4**_-EBNA1 and the Zn^2+^-**ZRL**_**5**_**P**_**4**_-EBNA1 complexes, suggesting that the Zn^2+^ ion and **ZRL**_**5**_ do not affect **P**_**4**_ in a **ZRL**_**5**_**P**_**4**_ molecule to bind to an EBNA1 monomer. This observation was further supported by the calculated binding energies using MMPBSA (Fig. 1*C* and *SI Appendix*, Fig. S10 and Table S3) (20).

### Synthesis of ZRL_5_P_4_ and Design/Synthesis of the Related EBNA1 Probes.

**ZRL**_**5**_**P**_**4**_ (Fig. 1*A*) was then synthesized, purified, and characterized. The synthetic route is shown in *SI Appendix*, Scheme S1, and the characterization processes were carried out (*SI Appendix*, Figs. S12–S14). Two other related probes, **ZRL**_**5**_**P**_**2**_ and **ZRL**_**5**_**P**_**6**_, were also synthesized to validate the EBNA1-blocking activity and to investigate the effects of the variation in a single amino acid in the blocking peptide. **ZRL**_**5**_**P**_**2**_ was used as a negative control, as it contains the EBNA1 binding motif (YFMVF) but lacks the NLS (RrRk) (14), and the rest of its structure is identical to **ZRL**_**5**_**P**_**4**_. On the other hand, **ZRL**_**5**_**P**_**6**_ contains the peptide **P**_**6**_ (YFIVF-GG-RrRK) which features the pentapeptide YFIVF to target the I^563^ in the common variant form of EBNA1 in various EBV strains (21). The purities of these 3 compounds were determined by high-performance liquid chromatography (HPLC) (*SI Appendix*, Fig. S11).

### Dual-Responsive Emission by ZRL_5_P_4_ toward Their Binding to Zn^2+^ and EBNA1.

**Fig. 2.**
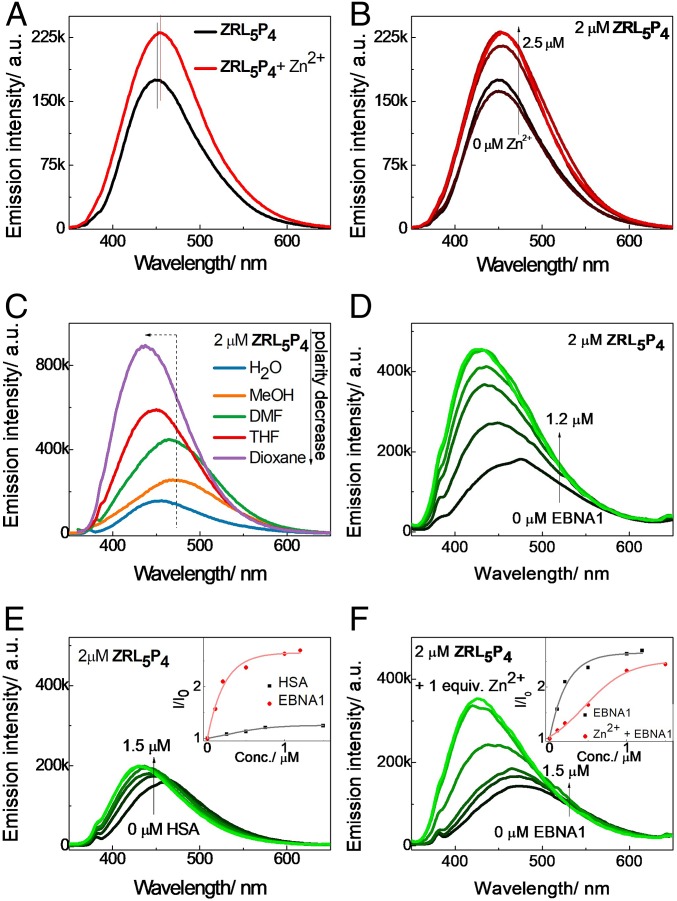
Dual-responsive emission of **ZRL**_**5**_**P**_**4**_ (2 µM; excitation 337 nm) toward Zn^2+^ and EBNA1. (*A*) Fluorescence spectral changes of **ZRL**_**5**_**P**_**4**_ in the presence of Zn^2+^ in aqueous solution (CH_3_CN/0.05 M Hepes [pH 7.4], 50:50). (*B*) Fluorescence spectral changes of **ZRL**_**5**_**P**_**4**_ upon gradual addition of Zn^2+^ in aqueous solution (CH_3_CN/0.05 M Hepes [pH 7.4], 50:50). (*C*) Solvation study of **ZRL**_**5**_**P**_**4**_ with a decrease of solvent polarity. Fluorescence spectral changes of **ZRL**_**5**_**P**_**4**_ in PBS upon addition of (*D*) EBNA1 and (*E*) HSA and upon addition of EBNA1 in the presence of (*F*) 1 equivalent Zn^2+^. (*E*, *Inset*) The selectivity of **ZRL**_**5**_**P**_**4**_ toward EBNA1 over HSA. (*F*, *Inset*) Interaction of **ZRL**_**5**_**P**_**4**_ with EBNA1 in the presence and absence of Zn^2+^. a.u., arbitrary units.

The fluorescent spectral changes of probes when bound to their targets are important indicators to reflect interactions (22). The absorption spectrum of **ZRL**_**5**_**P**_**4**_ in aqueous solution (Hepes) was first determined (*SI Appendix*, Fig. S15). The emission of **ZRL**_**5**_**P**_**2**_, **ZRL**_**5**_**P**_**4**_, and **ZRL**_**5**_**P**_**6**_ was then examined in the absence and presence of Zn^2+^ in aqueous solution (CH_3_CN/0.05 M Hepes [pH 7.4], 50:50) (Fig. 2*A* and *SI Appendix*, Fig. S16). Only **ZRL**_**5**_**P**_**4**_ showed a 1.3-fold emission enhancement with a 4-nm red-shifted emission (451 to 455 nm) upon the addition of Zn^2+^, while the other 2 probes showed either a slightly blue-shifted emission (**ZRL**_**5**_**P**_**2**_, 448 to 445 nm) or an unchanged maximum emission wavelength (**ZRL**_**5**_**P**_**6**_, 438 nm). We then focused on working out the detailed kinetics of the enhanced red-shifted emission mediated by **ZRL**_**5**_**P**_**4**_. We measured the luminescence of **ZRL**_**5**_**P**_**4**_ upon titration with various concentrations of Zn^2+^ to check their binding stoichiometry. A gradual red-shifted emission with a concomitant increase in the emission intensity was observed, and the change ceased when 1 equivalent Zn^2+^ had been added. This phenomenon indicates that **ZRL**_**5**_**P**_**4**_ and Zn^2+^ have a 1:1 stoichiometric ratio (Fig. 2*B*). In addition, the binding selectivity of **ZRL**_**5**_**P**_**4**_ toward Zn^2+^ versus other heavy transition metal ions was measured (*SI Appendix*, Fig. S17). Some of those metal ions decreased the emission of **ZRL**_**5**_**P**_**4**_ to different levels; while some showed blue-shifted emission others did not affect the emission. None of them showed a responsive emission similar to the one of Zn^2+^.

We next performed a solvation study to determine the ICT characteristic of each probe, as this mechanism contributes to the responsive signal emitted by the fluorophore in our EBNA1 probes when binding to EBNA1. The ICT-mediated emission is strongly solvent-dependent: The emission is enhanced and blue-shifted with decreasing solvent polarity. Also, the ICT emission is extremely sensitive to changes in the microenvironment and is generally increased when the microenvironment becomes hydrophobic, that is, when **ZRL**_**5**_**P**_**4**_ is bound to the EBNA1 monomer. Thus, this system can be utilized as a protein binding indicator (23). Emission changes of **ZRL**_**5**_**P**_**2**_, **ZRL**_**5**_**P**_**4**_, and **ZRL**_**5**_**P**_**6**_ were then measured as a function of solvent polarity. As can be seen in Fig. 2*C* and *SI Appendix*, Fig. S18, **ZRL**_**5**_**P**_**4**_ was shown to have the best ICT characteristics. Although the emission of **ZRL**_**5**_**P**_**6**_ was also enhanced, no blue shift was observed, however. **ZRL**_**5**_**P**_**4**_ was then evaluated for its binding activity toward the EBNA1 DBD protein. It was found that the addition of 1.2 µM EBNA1 DBD to **ZRL**_**5**_**P**_**4**_ caused a 2.7-fold emission enhancement, which was blue-shifted by 39 nm (466 to 427 nm) (Fig. 2*D*). When the irrelevant human serum albumin (HSA) was used as a negative control protein, the titration of HSA to **ZRL**_**5**_**P**_**4**_ showed only a slight increase in its emission intensity (Fig. 2*E*). Fig. 2*E*, Inset clearly shows that the binding of **ZRL**_**5**_**P**_**4**_ was highly selective for EBNA1 over HSA. In addition, the fluorescence response of **ZRL**_**5**_**P**_**4**_ was also measured in the presence of Zn^2+^ (Fig. 2*F*). A 2.4-fold enhancement with a 51 nm (477 to 426 nm) blue-shifted emission was observed, suggesting that the presence of Zn^2+^ did not significantly affect **ZRL**_**5**_**P**_**4**_-EBNA1 binding, as the same order of enhanced emission in the absence of Zn^2+^ was observed (Fig. 2*D*). This observation was further supported by the above MD simulation results (Fig. 1*C*).

### Confirmation of the Interaction of ZRL_5_P_4_ with Zn^2+^ by NMR Study.

To characterize the mechanism of how **ZRL**_**5**_**P**_**4**_ interacts with Zn^2+^, ^1^H NMR titration analysis of **ZRL**_**5**_**P**_**4**_ with and without 1 equivalent Zn^2+^ were conducted. The amide–imidic acid tautomer-binding mode of a similar Zn^2+^ chelator was adopted to analyze if our Zn^2+^-chelating fluorophore **ZRL**_**5**_ interacts with Zn^2+^ in a similar fashion in different solvents (18). We found that the Zn^2+^ chelate complex is an amide tautomer in CH_3_CN, whereas it is an imidic tautomer in DMSO. The ^1^H NMR spectroscopy analysis of compound 5 (the precursor of **ZRL**_**5**_) with/without Zn^2+^ in CD_3_CN and DMSO-d_6_ was conducted. Compound 5 was used as a substitute for **ZRL**_**5**_**P**_**4**_ because the presence of its peptide moiety will hinder an accurate Zn^2+^ binding analysis. Consistent with the previous study (18), proton 8 showed a large upfield shift from 10.93 to 9.43 parts per million (ppm) in CD_3_CN but showed a down-field shift from 10.72 to 10.84 ppm in DMSO-d_6_ (*SI Appendix*, Fig. S19). These findings have confirmed the 2 binding modes of **ZRL**_**5**_**P**_**4**_ in MeCN and DMSO, indicating that the Zn^2+^-triggered amide tautomerization can also occur for **ZRL**_**5**_**P**_**4**_ (Fig. 1*B*).

### ZRL_5_P_4_ Selectively Prevents Oligomerization of EBNA1 and Diminishes *oriP*-Enhanced Transactivation by EBNA1.

**Fig. 3.**
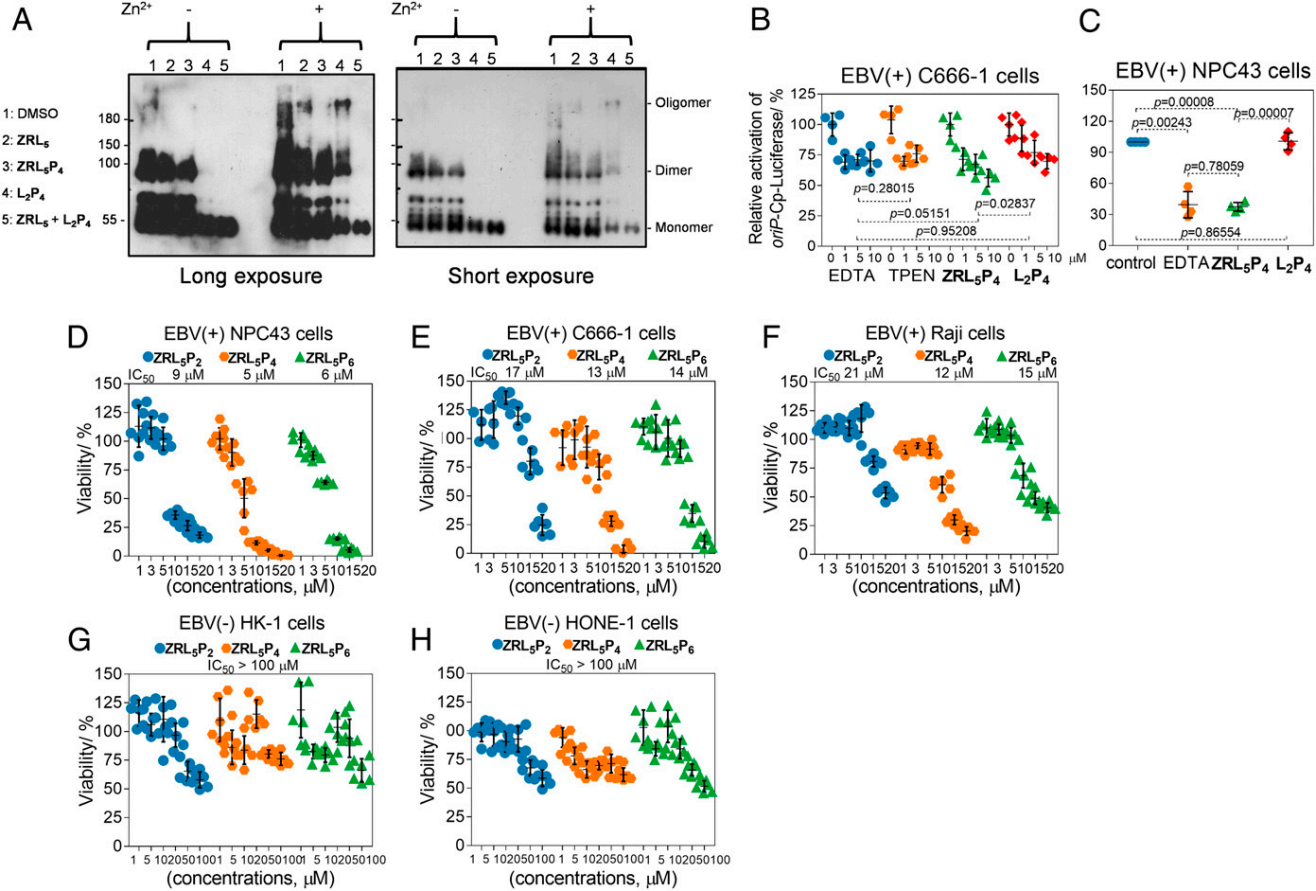
**ZRL**_**5**_**P**_**4**_ inhibits EBNA1 oligomerization and transactivation and cell viability. (*A*) Full- length EBNA1 self-association in the absence/presence of Zn^2+^ by **ZRL**_**5**_/**ZRL**_**5**_**P**_**4**_/**L**_**2**_**P**_**4**_/**ZRL**_**5**_ + **L**_**2**_**P**_**4**_; 0.1% DMSO serves as the solvent control. Long and short exposure images of the same blot are shown for comparison of various forms of EBNA1 self-association. Analysis of the effects of EBNA1 probes (**ZRL**_**5**_**P**_**4**_, **L**_**2**_**P**_**4**_) on the *oriP*-enhanced transactivation in EBV-positive (*B*) C666-1 and (*C*) NPC43 cells. The transactivation activities were detected by the *oriP*-Cp-luciferase reporter. EDTA and TPEN are the known chelators of Zn^2+^. (*D*–*H*) Cytotoxic activities of the EBNA1 probes **ZRL**_**5**_**P**_**2**_, **ZRL**_**5**_**P**_**4**_, and **ZRL**_**5**_**P**_**6**_ in the EBV-positive and -negative cell lines. Cytotoxicity of EBV-positive (*D*) NPC43 cells, (*E*) C666-1 cells, and (*F*) Raji cells (concentrations 1, 3, 5, 10, 15, and 20 µM) and EBV-negative (*G*) HK-1 cells and (*H*) HONE-1 cells (concentrations 1, 5, 10, 20, 50, and 100 µM) were measured by the MTT assay. Cells were treated with different probes and then incubated for 5 d to test their cytotoxicity (half of the medium was replaced every 4 d with fresh medium containing the appropriate concentration of the probes). Data are expressed as the means ± SD.

After the chemical and physical properties of **ZRL**_**5**_**P**_**4**_ were characterized, its ability to inhibit EBNA1 dimerization/oligomerization in the absence and presence of Zn^2+^ was assayed and was compared with the previous **L**_**2**_**P**_**4**_ EBNA1 probe. Zn^2+^ is necessary for the UR1-mediated self-association, and UR1 maps to the residues 64 to 89 of the amino terminus but not to the EBNA1 DBD. Thus, the full-length EBNA1 rather than the EBNA1 DBD was used. The Zn^2+^-chelating fluorophore, **ZRL**_**5**_, by itself was also included to determine the role of disruption of Zn^2+^ alone in the EBNA1 self-association. In the absence of Zn^2+^, the EBNA1 dimer formation was drastically prevented by **L**_**2**_**P**_**4**_ alone or in combination with **ZRL**_**5**_, whereas **ZRL**_**5**_**P**_**4**_ and **ZRL**_**5**_ had no obvious effect on the EBNA1 dimer (Fig. 3*A*). Interestingly, the EBNA1 oligomer could only be formed in the presence of Zn^2+^, and this oligomer was nearly completely abolished by **ZRL**_**5**_**P**_**4**_, but the dimer was not significantly affected by this probe. Whereas **L**_**2**_**P**_**4**_ could only partially inhibit the EBNA1 dimer but had no significant effect on the oligomer in the presence of Zn^2+^, **ZRL**_**5**_ had no obvious influence on both dimer and oligomer (Fig. 3*A*). These results indicate that the presence of Zn^2+^ can reinforce the dimerization and lead to oligomerization, and the exhaustion of Zn^2+^ ion in close proximity to EBNA1 by **ZRL**_**5**_**P**_**4**_ can eradicate the oligomerization. On the other hand, the combination treatment of **L**_**2**_**P**_**4**_ with **ZRL**_**5**_ could completely inhibit both dimer and oligomer formation. It is likely that these 2 compounds can work synergistically to eradicate the various forms of self-association of EBNA1. We then compared the effects of **ZRL**_**5**_**P**_**4**_ and **L**_**2**_**P**_**4**_ on the *oriP*-enhanced transactivation and investigated the role of the Zn^2+^ chelator in **ZRL**_**5**_**P**_**4**_ in disrupting the EBNA1 function. EBV-positive NPC C666-1 and NPC43 cells were exposed to **ZRL**_**5**_**P**_**4**_ and **L**_**2**_**P**_**4**_, and the luciferase reporter assay was performed. Two chelators known to have high specificity for Zn^2+^, ethylenediaminetetraacetic acid (EDTA) and N,N,N′,N′-tetrakis(2-pyridylmethyl)ethylenediamine (TPEN), were included as positive control agents. **ZRL**_**5**_**P**_**4**_ diminished the transactivation by EBNA1 in both C666-1 and NPC43 cell lines, and **L**_**2**_**P**_**4**_ could be only effective in C666-1 (Fig. 3 *B* and *C*). The effect of **ZRL**_**5**_**P**_**4**_ was found to be more potent than **L**_**2**_**P**_**4**_ in both C666-1 (*P* = 0.02837) and NPC43 cell lines (*P* = 0.00007) (Fig. 3 *B* and *C*). These data suggest that the addition of Zn^2+^ is more effective in reducing the EBNA1 transactivation activity, and disrupting the EBNA1 oligomer seems more critical than its dimer.

### ZRL_5_P_4_ Reduces the Viability of EBV-Positive Cells and Localizes to Their Nuclei.

We next measured the cell viability of a panel of EBV-positive (NPC43 and C666-1 NPC cells and Raji lymphoma cells) and EBV-negative (HK-1 and HONE-1 NPC cells) cells treated with **ZRL**_**5**_**P**_**2**_, **ZRL**_**5**_**P**_**4**_, and **ZRL**_**5**_**P**_**4**_ (Fig. 3 *D*–*H*). Both C666-1 and NPC43 have Y561F“M”VF565, while Raji has Y561F“I”VF565 at the DBD. Obvious cytotoxicity was observed in these 3 EBV-positive cells, even when treated with low dosages of 1 to 20 µM probes. **ZRL**_**5**_**P**_**4**_ and **ZRL**_**5**_**P**_**6**_ displayed very similar growth-inhibitory effects, with **ZRL**_**5**_**P**_**2**_ (lacking the NLS) being the least efficient one. In contrast, treatments with **ZRL**_**5**_**P**_**4**_ and **ZRL**_**5**_**P**_**6**_ in EBV-negative cells only showed <50% inhibition at a much higher dose (100 µM) (Fig. 3 *G* and *H*), indicating that the inhibitory activities are EBV-specific. When compared to the previous cytotoxicity study of **L**_**2**_**P**_**4**_ (median infectious dose; 23 µM in NPC43, 27 µM in C666-1, and 27 µM in Raji cells), **ZRL**_**5**_**P**_**4**_ is likely to be more potent, suggesting that the exploitation of the Zn^2+^ chelator enhances the cytotoxic activity, regardless of epithelial or lymphoid origin.

**Fig. 4.**
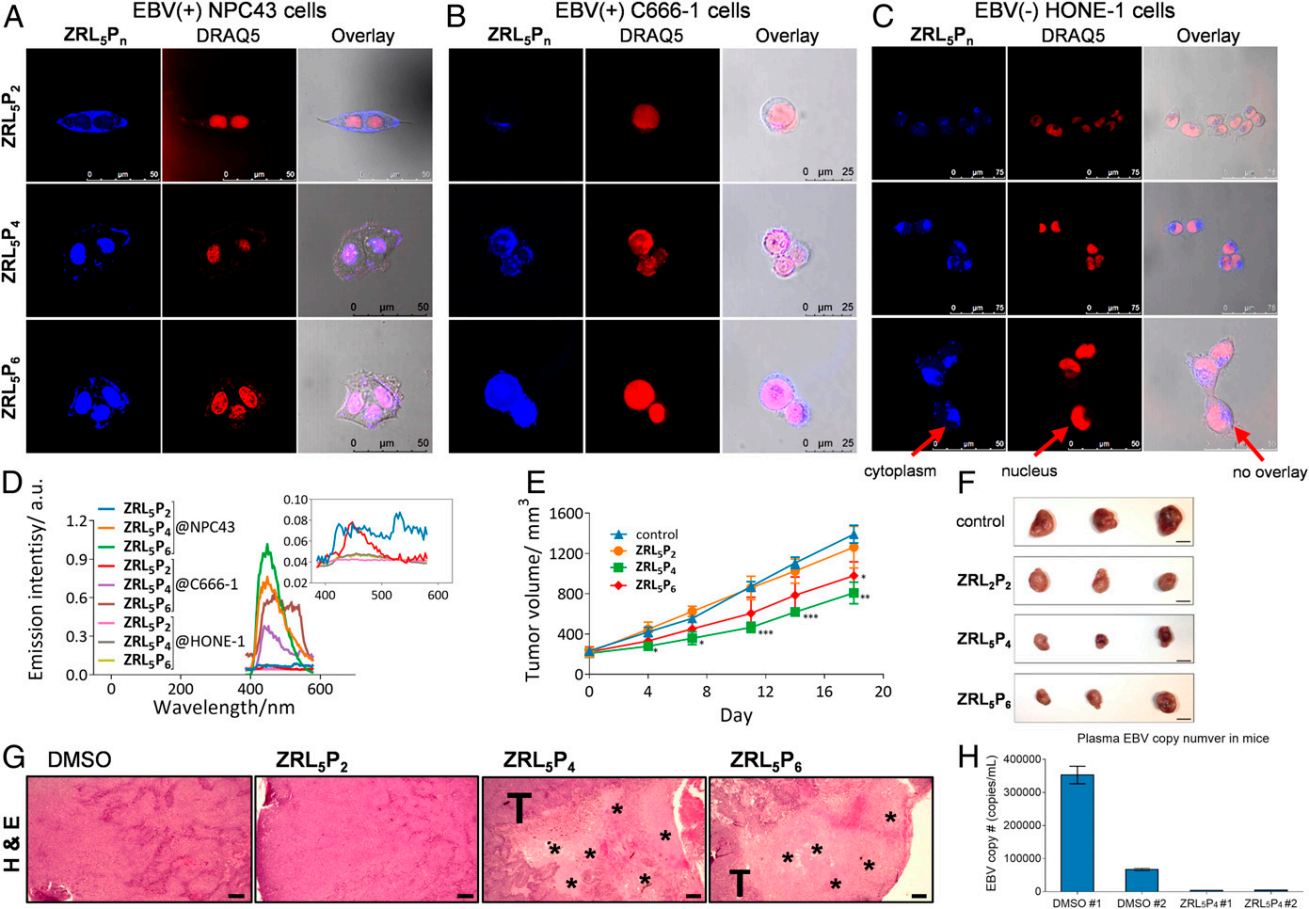
Nuclear localization of the EBNA1 probes and their in vivo antitumor activities. (*A*–*C*) Two-photon fluorescence imaging of **ZRL**_**5**_**P**_**2**_, **ZRL**_**5**_**P**_**4**_, and **ZRL**_**5**_**P**_**6**_ in living EBV-positive (*A*) NPC43 cells and (*B*) C666-1 cells and EBV-negative (*C*) HONE-1 cells. **ZRL**_**5**_**P**_**n**_, signal emitted from the respective EBNA1 probe. DRAQ5 is a fluorescent dye used to label the cell nuclei of the living cells as indicated. (*D*) In vitro emission spectra (from confocal microscopy) of **ZRL**_**5**_**P**_**2**_, **ZRL**_**5**_**P**_**4**_, and **ZRL**_**5**_**P**_**6**_ in the nucleus of EBV-positive NPC43 and C666-1 and EBV-negative HONE-1 cells. Emission intensity was much greater for **ZRL**_**5**_**P**_**4**_ and **ZRL**_**5**_**P**_**6**_ in EBV-positive cells. (*E*) In vivo antitumor activity of **ZRL**_**5**_**P**_**2**_, **ZRL**_**5**_**P**_**4**_, and **ZRL**_**5**_**P**_**4**_. Mice transplanted with C666-1–derived tumors were treated twice weekly with 4 µg per injection of the probes for 18 d. Throughout the treatment period, tumor volumes were measured. At the experimental endpoint, tumors were excised. Data are expressed as the means ± SEM. *P < 0.05; **P < 0.01; ***P < 0.001 vs. control (0.1% DMSO). (Scale bars, 10 mm.) (*F*) Representative photographs of tumors. (*G*) Representative H&E staining images of tumor sections derived from the above in vivo animal study. Cell necrosis (acellular areas indicated by asterisk) was observed in the tumor nodules treated with **ZRL**_**5**_**P**_**4**_ and **ZRL**_**5**_**P**_**6**_. T, adjacent area with tumor cells. Magnification, 400×. (Scale bars, 20 µm.) (*H*) Response of plasma EBV DNA levels in mice transplanted with C666-1 cells after the treatment of **ZRL**_**5**_**P**_**4**_. The circulating EBV DNA level of each mouse (DMSO #1, #2; **ZRL**_**5**_**P**_**4**_ #1, #2) is shown.

To show that entry into the nuclei is essential for the EBNA1 probes to inhibit tumor cell viability, subcellular localization of **ZRL**_**5**_**P**_**2**_, **ZRL**_**5**_**P**_**4**_, and **ZRL**_**5**_**P**_**6**_ were evaluated in both EBV-positive (C666-1 and NPC43) and EBV-negative (HONE-1) cell lines, using 2-photon excitation microscopy (λ_ex_: 700 nm). After incubation for 3 h, the fluorescence signals were collected in the blue channel. **ZRL**_**5**_**P**_**4**_ and **ZRL**_**5**_**P**_**6**_ were primarily located in the nuclei of EBV-positive cells (Fig. 4 *A*, *B*, and *D*) and this is in contrast to the imaging results of **L**_**2**_**P**_**4**_ we previously reported, which showed that the majority of **L**_**2**_**P**_**4**_ was localized to the cytoplasm, and only a small portion was found in the nuclei (14). On the other hand, **ZRL**_**5**_**P**_**2**_ was only found in the cytoplasm (Fig. 4 *A*, *B*, and *D*) and all 3 probes showed no nuclear localization in EBV-negative cells (Fig. 4 *C* and *D*). The EBNA1 expression detected by Western blot in these 3 cell lines correlates quite well with the signal intensities reflected by **ZRL**_**5**_**P**_**4**_ (*SI Appendix*, Fig. S20*A*). The cellular uptake of the new and old EBNA1 probes by EBV-positive cells was also compared and it was found that there the uptake of **L**_**2**_**P**_**4**_ was faster than **ZRL**_**5**_**P**_**4**_ into their nuclei; however, **L**_**2**_**P**_**4**_ also accumulated in the cytoplasm (*SI Appendix*, Fig. S20*B*). The inclusion of a Zn^2+^ chelator seems to decrease the uptake rate and increase the specificity of **ZRL**_**5**_**P**_**4**_. Taken together, all these imaging results indicated that the presence of both the Zn^2+^ chelator and NLS is critical for more specific nuclear localization of the new EBNA1 probes to selectively stain the EBV-positive cells.

### ZRL_5_P_4_ Inhibits the Growth of EBV-Positive Tumors in BALB/c Nude Mice.

The in vivo effects of the 3 EBNA1 probes in the C666-1 xenograft were then examined. Treatment with **ZRL**_**5**_**P**_**2**_, **ZRL**_**5**_**P**_**4**_, and **ZRL**_**5**_**P**_**6**_ did not cause any significant changes in body or organ weights when compared to vehicle control (*SI Appendix*, Figs. S21 and S22), indicating that the 3 probes did not exhibit a toxic effect in vivo. Tumor growth was significantly inhibited by treatment with **ZRL**_**5**_**P**_**4**_ and **ZRL**_**5**_**P**_**6**_. Both the average tumor volume and tumor weight of mice treated with **ZRL**_**5**_**P**_**4**_ or **ZRL**_**5**_**P**_**6**_ was significantly decreased, compared to the control (Fig. 4 *E* and *F* and *SI Appendix*, Fig. S23). There was no significant difference in either tumor volume or tumor weight when comparing the effects of **ZRL**_**5**_**P**_**4**_ and **ZRL**_**5**_**P**_**6**_. **ZRL**_**5**_**P**_**2**_ without NLS, however, did not significantly affect tumor growth. For the control HeLa xenograft, there was no significant difference in either tumor volume or tumor weight between the control mice and those treated with the probes (*SI Appendix*, Fig. S24). When the tumor sections were stained with hematoxylin/eosin (H&E) to examine the tissue morphology, we found that cell necrosis was much more frequently observed in the tumor tissues with the treatments of **ZRL**_**5**_**P**_**4**_ or **ZRL**_**5**_**P**_**6**_ than with the **ZRL**_**5**_**P**_**2**_ and the solvent control (Fig. 4*G*). The cell death could be due to the cytotoxic activities of **ZRL**_**5**_**P**_**4**_ and **ZRL**_**5**_**P**_**6**_ observed in the MTT [3-(4,5-dimethylthiazol-2-yl)-2,5-diphenyltetrazolium bromide] assay for C666-1 cells (Fig. 3*E*), and that can also explain why the tumors shrank after these treatments. When performing an independent set of in vivo experiments with transplanted C666-1–derived tumors, the plasma EBV DNA dramatically dropped from ∼350,000 and ∼67,000 to ∼3,900 and ∼4,400 copies after treatment with **ZRL**_**5**_**P**_**4**_ (Fig. 4*H*). This circulating EBV DNA load was directly proportional to the viable cell areas in the tumors and inversely associated with body weight (*SI Appendix*, Fig. S25). Hence, the plasma EBV DNA is likely a biomarker for predicting the effectiveness of **ZRL**_**5**_**P**_**4**_.

### Reactivation of the EBV Lytic Cycle by ZRL_5_P_4_.

**Fig. 5.**
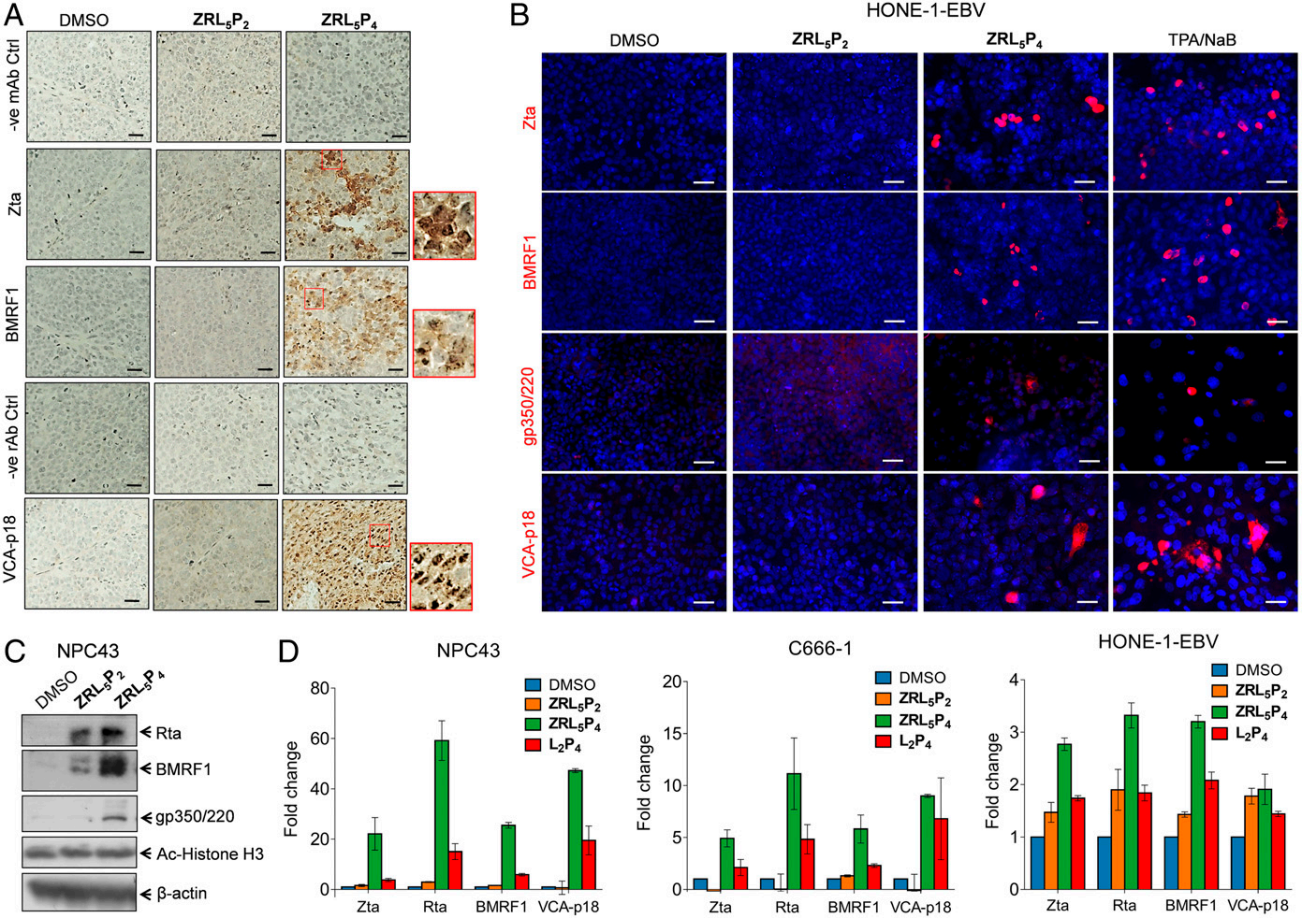
EBV lytic induction analysis of EBV-positive tumors and cells in response to the EBNA1 probes. (*A*) IHC analysis of lytic proteins Zta, BMRF1, and VCA-p18 in the transplanted C666-1–derived tumor tissues as described in Fig. 4 *E*–*G*. Representative results are shown. Nuclear and cytoplasmic staining of Zta, BMRF1, and VCA-p18 are observed in response to **ZRL**_**5**_**P**_**4**_. Insets (in the red boxes) are the enlarged images to indicate the cellular localization of the 3 proteins. Negative control staining images are included for the mouse antibodies (mAb) against Zta and BMRF1 and for the rat antibody (rAb) against VCA-p18. Magnification, 400×. (Scale bars, 20 µm.) (*B*) Representative images of immunofluorescent analysis of EBV lytic proteins, Zta, BMRF1, gp350/220, and VCA-p18 in HONE-1-EBV cells in response to 10 µM **ZRL**_**5**_**P**_**2**_ or **ZRL**_**5**_**P**_**4**_. TPA/NaB (20 ng/mL TPA, 3 mM NaB) serves as a positive control of lytic induction. The presence of these 4 lytic proteins is indicated by red signals. The nuclei were stained with DAPI. (Scale bars, 50 µm.) (*C*) Western blot analysis of Rta, BMRF1, and gp350/220 EBV lytic proteins in NPC43 cells cultured with or without 10 µM **ZRL**_**5**_**P**_**2**_ and **ZRL**_**5**_**P**_**4**_ for 7 d. Acetylated histone H3 (Ac-Histone H3) was also detected. β-actin serves as the loading control. (*D*) Gene expression analysis of EBV lytic genes, Zta, Rta, BMRF1, and VCA-p18 in NPC43, C666-1, and HONE-1-EBV cells cultured with or without 10 µM **ZRL**_**5**_**P**_**2**_, **ZRL**_**5**_**P**_**4**_, and **L**_**2**_**P**_**4**_, for 7 (NPC43 cells), 3 (C666-1 cells), or 5 (HONE-1-EBV) d. The gene expression was analyzed by qRT-PCR. The fold change of relative gene expression after each treatment was compared with the solvent control (DMSO). Data are expressed as the means ± CI.

Interestingly, when tumor sections of Fig. 4*G* were analyzed with immunohistochemistry (IHC), the EBV immediate early, early, and late lytic proteins, Zta, BMRF1, and VCA-p18, were mainly detected in the tumors injected with **ZRL**_**5**_**P**_**4**_ (Fig. 5*A*). Nuclear and cytoplasmic staining of Zta, BMRF1, and VCA-p18 was observed in these tumor tissues. After treatment with **ZRL**_**5**_**P**_**4**_, ∼10 and 15% tumor areas were positive for Zta and BMRF1, respectively, and more than 80% was VCA-p18 positive. On the other hand, only negative staining was detected in the solvent control, and 0 to 2% was observed in **ZRL**_**5**_**P**_**2**_ for these 3 lytic proteins.

Furthermore, other EBV-infected cell lines, NPC43 and HONE-1-EBV (the recombinant EBV-containing HONE-1), were included for the lytic phase analysis. The protein expression of the immediate early, early, and late lytic genes, Zta, BMRF1, gp350/220, and VCA-p18, was studied in NPC43, HONE-1-EBV, and C666-1 cell lines in response to 10 µM **ZRL**_**5**_**P**_**2**_ and **ZRL**_**5**_**P**_**4**_. Immunofluorescence (IF) staining was performed, and the conventional chemical inducer tetradecanoyl phorbol acetate (TPA) (20 ng/mL) with sodium butyrate (3 mM) (TPA/NaB) (2) was also included as a positive control. The positively stained cells for all 4 proteins were more frequently observed in **ZRL**_**5**_**P**_**4**_, and the response to **ZRL**_**5**_**P**_**2**_ was similar to that of the solvent control in HONE-1-EBV cells (Fig. 5*B*). Although the fold changes of the 4 lytic proteins in response to TPA/NaB were generally higher than **ZRL**_**5**_**P**_**4**_, the increased levels of the 2 late proteins gp350/220 and VCA-p18 were not statistically significant (*SI Appendix*, Fig. S26). A similar observation of lytic protein induction was obtained in the other 2 EBV-positive cell lines (*SI Appendix*, Figs. S27 and S28*A*).

The IF results in NPC43 and C666-1 cells were validated by Western blot analysis, which showed that the expression levels of a number of lytic proteins were dramatically induced by **ZRL**_**5**_**P**_**4**_ (Fig. 5*C* and *SI Appendix*, Fig. S28*B*). Although the expression of Rta and BMRF1 was also induced by **ZRL**_**5**_**P**_**2**_ in NPC43 cells, the increased levels of expression were much weaker than those of **ZRL**_**5**_**P**_**4**_ and the gp350/220 levels were similar to the solvent control. As some histone modifiers such as TPA/NaB can induce EBV lytic reactivation, we examined the effects of **ZRL**_**5**_**P**_**4**_ on the acetylation of histone H3 in 2 NPC cells lines. Our agent had no significant effect on both cell lines (Fig. 5*C* and *SI Appendix*, Fig. S28*B*), and it seems that histone modification might not be directly related to EBV reactivation induced by **ZRL**_**5**_**P**_**4**_.

We then compared the effects of our new **ZRL**_**5**_**P**_**4**_ versus the old **L**_**2**_**P**_**4**_ probes on the EBV lytic induction. The gene expression of EBV lytic genes including Zta, Rta, BMRF1, and VCA-p18, was studied in 3 EBV-positive cell lines (NPC43, HONE-1-EBV, and C666-1) in response to 10 µM **ZRL**_**5**_**P**_**2**_, **ZRL**_**5**_**P**_**4**_, or **L**_**2**_**P**_**4**_. The gene expression was quantified by the accurate qRT-PCR analysis. The expression of all 4 lytic genes was strikingly induced by **ZRL**_**5**_**P**_**4**_, whereas their expression in response to **ZRL**_**5**_**P**_**2**_ was similar to that of the solvent control (Fig. 5*D*). When compared with **ZRL**_**5**_**P**_**4**_, **L**_**2**_**P**_**4**_ could only induce the 4 lytic genes with much lower levels in NPC43 and C666-1 cells, and in HONE-1-EBV cells the changes of these genes were either comparable to or lower than the negative control **ZRL**_**5**_**P**_**2**_. As can be seen, the Zn^2+^ chelator in **ZRL**_**5**_**P**_**4**_ is critical in initiation of EBV reactivation.

**Fig. 6.**
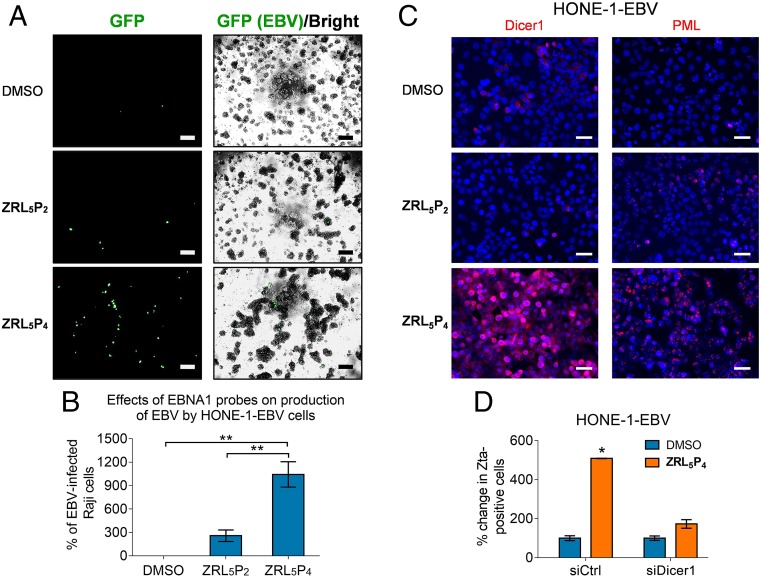
Production of infectious EBV particle in response to **ZRL**_**5**_**P**_**4**_. The HONE-1-EBV cell line, which expresses GFP to indicate the presence of the EBV genome, was used. This cell line was treated with 10 µM **ZRL**_**5**_**P**_**2**_ or **ZRL**_**5**_**P**_**4**_ for 4 d, and the viral particles released in the culture medium were detected by the Raji cell assay. The culture medium was added to Raji cells for 3 d, and GFP expression reflects the reinfection by the HONE-1–released EBV particles. (*A*) Representative results are shown. The GFP signal was detected by ultraviolet light exposure, and the cell morphology was captured by phase-contrast light microscopy and the bright-field image was merged with the GFP image. Magnification, 400×. (Scale bars, 100 µm.) (*B*) Relative average viral titer in response to **ZRL**_**5**_**P**_**2**_ or **ZRL**_**5**_**P**_**4**_ was compared with the solvent control (DMSO). The Raji cell assay was performed in triplicate for each treatment. **P < 0.01, statistically significant difference. Data are expressed as the means ± SD. (*C*) Representative images of immunofluorescent analysis of Dicer1 and PML in HONE-1-EBV cells in response to 10 µM **ZRL**_**5**_**P**_**2**_ or **ZRL**_**5**_**P**_**4**_. The nuclei were counterstained with DAPI and indicated in blue. (Scale bars, 50 µm.) (*D*) Comparison of the number of Zta-positive cells after treatment with **ZRL**_**5**_**P**_**4**_ in the presence versus the absence of Dicer1. HONE-1-EBV and NPC43 were included. Gene silencing of Dicer1 was achieved by siRNA transfection. Zta was detected by immunofluorescent analysis. Control siRNA (siCTL) was used as a negative control. Relative percentage of Zta-positive was compared against the DMSO solvent control in the siRNA control cells. *P < 0.05.

In order to verify if the EBV particles with infectious properties could actually be generated by **ZRL**_**5**_**P**_**4**_, HONE-1-EBV cells were used. The presence of the virions was detected by infecting Raji cells (an established B cell line), because the recombinant EBV genome in HONE-1-EBV encodes a green fluorescence protein (GFP), and the GFP-expressing Raji cells reflect the production of virions by the HONE-1-EBV cells. As can be seen, 10 µM **ZRL**_**5**_**P**_**4**_ could lead to the production of virions and which was 10.4-fold more than the DMSO control (*P* = 0.009) and was ∼4-fold more than the NLS-null version **ZRL**_**5**_**P**_**2**_ (*P* = 0.006) (Fig. 6 *A* and *B*). Although the viral titer of **ZRL**_**5**_**P**_**2**_ was 2.6-fold higher than the solvent control, the difference was not significant (*P* = 0.06). Taken together, the entry of **ZRL**_**5**_**P**_**4**_ into the nuclei of EBV-infected cells can induce the reactivation of EBV, which might mediate the shrinkage of the transplanted C666-1 tumors (Fig. 4 *E*–*G*).

To study the underlying mechanism(s) of how **ZRL**_**5**_**P**_**4**_ induces EBV lytic induction, the change in expression of Dicer and PML were examined, as previous studies indicate that these 2 proteins are associated with EBNA1-associated lytic induction (24, 25). The in situ protein expression of both Dicer1 and PML was consistently up-regulated in 2 NPC cell lines in response to **ZRL**_**5**_**P**_**4**_ (Fig. 6*C* and *SI Appendix*, Fig. S29*A*). The increased protein expression of Dicer1 and PML was validated by Western blot (*SI Appendix*, Fig. S29*B*). To show the specific induction of these 2 proteins by **ZRL**_**5**_**P**_**4**_, the expression was checked in the EBV-negative HONE-1 cells, and there was no induction of Dicer1 or PML in the **ZRL**_**5**_**P**_**4**_-treated cells (*SI Appendix*, Fig. S30*A*). Zta protein expression and cell viability were examined in both **ZRL**_**5**_**P**_**4**_-treated HONE-1 and HONE-1-EBV, to show that **ZRL**_**5**_**P**_**4**_ only had lytic-inducing and antitumor activities in the EBV-positive cells (*SI Appendix*, Fig. S30 *A*–*C*). Thus, the induction of Dicer1 and PML by **ZRL**_**5**_**P**_**4**_ is EBV-specific. To further demonstrate whether the lytic induction is mediated by Dicer1 and/or PML, we depleted their expression in EBV-positive cells and examined the expression of the key lytic protein Zta in response to **ZRL**_**5**_**P**_**4**_. When the expression of Dicer1 was depleted (*SI Appendix*, Figs. S29*B* and S31*A*), the **ZRL**_**5**_**P**_**4**_-induced Zta expression was almost completely attenuated in 2 EBV-positive cell lines (Fig. 6*D*), whereas the knockdown of PML itself had led to the induction of Zta expression in NPC43 cells, and **ZRL**_**5**_**P**_**4**_ could further enhance the Zta expression (*SI Appendix*, Fig. S31*B*). As can be seen, Dicer1 is likely a mediator which can be induced by **ZRL**_**5**_**P**_**4**_ to initiate the EBV reactivation.

## Discussion

EBNA1 is known as a dimeric viral protein encoded by EBV; our previous work has confirmed **P**_**4**_ (CAhxYFMVFGGRrRK) is a physical blocker to hamper EBNA1 dimerization, and hence inhibit the functions of EBNA1 as well as the growth of EBV-positive cells. Interestingly, Zn^2+^ was also recently reported as a cofactor of EBNA1 self-association; it mediates interactions of the UR1 region between 2 EBNA1 monomers through the formation of “zinc finger” structure. We thereby constructed a probe, **ZRL**_**5**_**P**_**4**,_ by taking advantages of its Zn^2+^-binding property. Unexpectedly, the Zn^2+^ chelator acts in close proximity to **P**_**4**_ to mainly disrupt the oligomerization rather than the dimerization of EBNA1 (Fig. 3*A*). Interestingly, the formation of EBNA1 oligomer was only observed in the presence of Zn^2+^ and could also stabilize the dimeric EBNA1. These results are in concordance with the previous studies that Zn^2+^ is required for self-association of EBNA1 at the UR1 domain where the Zn^2+^ coordination between 2 EBNA1 monomers occurs (4). The “self-association” can represent the dimer formation from 2 homomonomers of EBNA1, as well as the formation of higher-order EBNA1 complexes. This hypothesis is supported by the simulation model suggested by Hussain et al. (26) that a Zn^2+^ ion links 2 EBNA1 dimers to form an oligomer to bind to DNA. This EBNA1 complex structure could possibly represent a hexameric ring, as previously suggested (27). As the UR1 domain was reported to be essential for the transactivation activity of EBNA1 (4, 28), that can explain why **ZRL**_**5**_**P**_**4**_ was much more effective than **L**_**2**_**P**_**4**_ in disrupting the *oriP*-mediated transcription, growth inhibition, and lytic induction. The increased potency of **ZRL**_**5**_**P**_**4**_ on the various EBNA1 and cellular activities upon **L**_**2**_**P**_**4**_ is also reflected by the cellular imaging for the EBV-positive cell lines (Fig. 4). The imaging results explain that the stronger inhibition can be attributed to the nuclear localization of the probe, where EBNA1 is primarily located, and that nuclear EBNA1 is of critical importance for its dependent function. In vivo administration of **ZRL**_**5**_**P**_**4**_ was demonstrated to shrink subcutaneous tumors (Fig. 4). The plasma EBV DNA can act as a biomarker for prediction of the effectiveness of treatment with **ZRL**_**5**_**P**_**4**_ (Fig. 4*H*). Surprisingly, the subsequent IHC staining indicated that the necrotic tumor tissues were likely due to the induction of EBV lytic cycle (Figs. 4 and 5). The EBV immediate early, early, and late lytic gene/protein expression analyses and the in vitro Raji cell infection assay showed that **ZRL**_**5**_**P**_**4**_ can induce the EBV lytic cycle and produce infectious EBV particles (Figs. 5 and 6 *A* and *B*). When compared with the conventional chemical inducer (a common histone deacetylase inhibitor), the potency of **ZRL**_**5**_**P**_**4**_ seems not as strong as the TPA/NaB treatment (Fig. 5*B* and *SI Appendix*, Fig. S26). However, the micromolar **ZRL**_**5**_**P**_**4**_ concentration is sufficient to induce the late lytic proteins in a similar magnitude to the millimolar NaB treatment. The status of histone acetylation was not significantly affected by **ZRL**_**5**_**P**_**4**_, mechanisms other than histone modification might be involved.

In concordance with our findings, it has been reported that the depletion of EBNA1 gene expression by small interfering RNA (siRNA) in the EBV-infected epithelial cell lines can activate spontaneous lytic cycle induction, indicating that EBNA1 has a functional role in suppressing reactivation of EBV (24). Previous studies indicate that EBNA1 can disrupt PML and Dicer expression, and these 2 proteins are required for EBV reactivation (24, 25). Our results show that **ZRL**_**5**_**P**_**4**_ could specifically enhance the expression of these 2 proteins in the EBV-positive cells, but this was not observed in the EBV-negative cells. This observation further strengthens the evidence for the selectivity of **ZRL**_**5**_**P**_**4**_, which only functions in the presence of EBNA1. Importantly, the presence of Dicer1 seems essential for the lytic induction by **ZRL**_**5**_**P**_**4**_, while the change in PML expression levels is also associated with the **ZRL**_**5**_**P**_**4**_-induced EBV reactivation. Our study also suggests that the function of EBNA1 and the role of EBV latent infection is associated with the maintenance of tumor cell survival through suppressing lytic cycle reactivation. Substantial advancement has been observed toward development of novel viral-reactivating agents for induction of cytocidal effects in various EBV-associated tumor cells. This strategy has eventually entered clinical phase I/II trials, and significant improvements in clinical outcome were observed in at least a portion of the EBV-positive lymphoma patients (2). A recent study by Messick et al. (29) has described a small molecule that inhibits the DNA binding activity of EBNA1; this molecule can suppress the growth of NPC and other EBV-associated tumors but does not appear to induce the EBV lytic cycle. The lytic inactivation could be due to mechanisms other EBNA1-DNA binding, such as induction of Dicer1, PML, and so on, as reflected by the present study. Thus, to our knowledge, no specific lytic cycle inducers against EBV genes or proteins have been established. As EBNA1 is a foreign protein to the host, in theory the elimination of EBV-associated tumor cells by **ZRL**_**5**_**P**_**4**_ warrants absolute specificity over all other lytic inducers.

On the other hand, we initially thought that the genetic variation at the EBNA1 dimerization sequence (the amino acid 563) could be an important factor for the design of EBNA1 probes. **ZRL**_**5**_**P**_**6**_ was constructed to target the EBNA1 protein with an “I” residue at the variation position. However, the results of various biological assays show that both **ZRL**_**5**_**P**_**4**_ and **ZRL**_**5**_**P**_**6**_ were almost equally effective in all these assays, indicating that this amino acid residue is not critical for disrupting the self-association of the EBNA1 monomer. These findings suggest that **ZRL**_**5**_**P**_**4**_ and **ZRL**_**5**_**P**_**6**_ should be equally potent for suppressing the EBNA1 proteins in various EBV stains.

In conclusion, we have used Zn^2+^ as an important cofactor for EBNA1 function and have constructed a series of EBNA1 probes with a Zn^2+^ chelator and an EBNA1-binding peptide. The Zn^2+^ chelator can preferentially suppress the higher-order form of EBNA1 complex to further enhance the inhibitory activities of our new-generation compound **ZRL**_**5**_**P**_**4**_. Importantly, **ZRL**_**5**_**P**_**4**_ reactivates EBV lytic induction, which is associated with the shrinkage of EBV-positive tumors in the animal model. This study successfully targeted a single protein (EBNA1) to induce the EBV lytic cycle. As EBNA1 is a foreign protein to the host, in theory this strategy of elimination of EBV-associated tumor cells warrants absolute specificity over all other lytic induction therapies. The current lytic analysis results also suggest that the function of EBNA1 as well as the role of EBV latent infection is associated with the maintenance of tumor cell survival through the suppression of the lytic cycle reactivation. Exhaustion of Zn^2+^ in the microenvironment in protein molecules might represent a new strategy in designing inhibitory agents to target pathogenic proteins in cancer development and other diseases.

## Materials and Methods

### General.

Unless otherwise stated, all chemicals were used as purchased without further purification. Peptides were ordered from GL Biochem (Shanghai) Ltd. Full-length EBNA1 with N-terminal His tag was purchased from Abcam (ab138345). The siRNAs were supplied by Thermo Fisher. Solvents were dried using standard procedures. Purification of **ZRL**_**5**_**P**_**2**_, **ZRL**_**5**_**P**_**4**_, and **ZRL**_**5**_**P**_**6**_ was performed on a Waters semipreparative HPLC system. NMR spectra were recorded on a Bruker 400-MHz NMR spectrometer, and the chemical shifts were referenced internally to tetramethylsilane or the corresponding solvent residues in parts per million; coupling constants are reported in hertz. High-resolution mass spectra, reported as *m*/*z*, were obtained on either a Bruker Autoflex MALDI-TOF or an Agilent 6450 UHD Accurate-Mass Q-TOF spectrometer. UV-visible absorption spectra were recorded on a Cary 8454 spectrometer.

### Syntheses.

All new compounds were characterized by ^1^H and ^13^C NMR spectroscopy and high-resolution mass spectrometry. Characterization data are shown in *SI Appendix*.

### MD Simulation.

All-atom unbiased MD simulations in AMBER 14 with aff99SBildn force field were used. The system preparation and simulation procedures were the same as those reported previously (14). In brief, a periodic boundary, cubic, TIP3 (30) explicit water box with a 20-Å buffer was used with charge neutralized by adding Cl^−^ ions. The system was then minimized and equilibrated by sander using 3 stages: 1) heating from 100 to 300 K in 20 ps, 2) adjusting the solvent density to 1 g/mL in 20 ps, and 3) equilibrating in 200 ps with NPT ensemble. A subsequent 100-ns NPT simulation was performed with CUDA-accelerated PMEMD (31, 32). A 2-fs time step and SHAKE-enabled settings were used for all of the equilibration and production stages. A Berendsen thermostat was adopted for temperature control at all stages.

### Luminescence Measurements.

Luminescent spectra were recorded on a Horiba Fluorolog-3 spectrofluorometer equipped with a xenon lamp. Specifically, the selectivity assay of **ZRL**_**5**_**P**_**4**_ toward Zn^2+^ among many heavy metal ions was performed in Hepes buffer (0.05 M, pH 7.4)/CH_3_CN (50:50) using perchlorate salts as the metal source [Zn(ClO_4_)_2_, Cd(ClO_4_)_2_, Cu(ClO_4_)_2_, Co(ClO_4_)_2_, Ni(ClO_4_)_2_, Hg(ClO_4_)_2_ and Mg(ClO_4_)_2_]. The selectivity assay of **ZRL**_**5**_**P**_**4**_ toward EBNA1 was measured in phosphate-buffered saline (PBS) buffer. Luminescence titration experiments were performed by gradually increasing the concentration of the analytes in the aqueous solution of **ZRL**_**5**_**P**_**4**_, including Zn^2+^, EBNA1, and HSA; the titration was stopped when the emission change of **ZRL**_**5**_**P**_**4**_ ceased.

### Dimerization/Oligomerization Assay.

Full-length EBNA1 (residues 1 to 641) with an N-terminal His tag (ab138345; Abcam) was used in this assay. Each 0.875 µg of EBNA1 was incubated without or with 50 µM Zn^2+^ at room temperature in the presence of 8 ng *oriP* DNA and 100 µM probe (buffer/**L**_**2**_**P**_**4**_/**ZRL**_**5**_**P**_**4**_) for 1 h at 37 °C to allow self-association to occur. After incubation, sodium dodecyl sulfate (SDS) loading buffer was added to each system, which was then separated using denaturing SDS/polyacrylamide gel electrophoresis, transferred onto a nitrocellulose membrane, and blotted with an antibody against the His tag (GeneTex); the obtained protein bands provided information of dimerization/oligomerization inhibition.

### Luciferase Reporter Assay for EBNA1 oriPI-Dependent Transactivation.

To study EBNA1-dependent transactivation, the luciferase vector J988F containing the EBV C promoter and *oriPI* (family of repeats) was constructed. The EBV C promoter and *oriPI* (nucleotides 7447 to 11412) regions were subcloned from the previously described plasmid pgCp(-3889)CAT (33, 34) as a HindIII fragment into the pGL3Basic luciferase vector (Promega). Correct sequences were ascertained by Sanger sequencing using the ABI PRISM Big Dye terminator cycle sequencing kit (Applied Biosystems). EBV-positive C666-1 and NPC43 cells were then transiently transfected with the J988F reporter plasmid. Cells were seeded in 12-well plates and cotransfected with the J988F plasmid (2 µg per well) and a pRL *Renilla* luciferase control reporter (500 ng per well) (Promega) using Lipofectamine 2000 (Invitrogen). After 24 h, the cells were treated with **ZRL**_**5**_**P**_**4**_, **L**_**2**_**P**_**4**_, EDTA, or TPEN (10 µM) for another 8 h. Cells were lysed with Passive Lysis Buffer (Promega), and the lysate was then transferred onto a white, opaque, 96-well plate. The luciferase activities were measured using the Dual Luciferase Reporter Assay System (Promega) with the GloMax 96 Microplate Luminometer (Promega). The pRL *Renilla* luciferase reporter was used as an internal control to normalize the transfection efficiency among the samples.

### Cell Culture.

Six cell lines were used in this work: the EBV-negative HK-1 and HONE-1 lines and the EBV-positive NPC43, C666-1, HONE-1-EBV, and Raji lines. HK-1, HONE-1, HONE-1-EBV, C666-1, and Raji cells were grown in RPMI medium 1640 supplemented with 10% fetal bovine serum (FBS) and 1% penicillin and streptomycin at 37 °C and 5% CO_2_. NPC43 cells were maintained in RPMI 1640 with 10% FBS and 4 µM Y27362 (inhibitor of Rho-associated, coiled-coil-containing protein kinase; Enzo Life Sciences). C666-1, HK-1, and HONE-1 cells were obtained from the Hong Kong NPC AoE Cell Line Repository, NPC43 and HONE-1-EBV were supplied by S.W.T., and Raji was supplied by ATCC. All of the cell lines were authenticated by using the AmpFℓSTR Identifier PCR Amplification kit (Life Technologies) (*SI Appendix*, Table S5) and were tested to be mycoplasma-negative by PCR.

### MTT Assay.

All cells were subcultured in 96-well plates at the optimal growth density (HK-1, 1 × 10^4^ cells/100 µL per well; HONE-1, 8 × 10^3^ cells/100 µL per well; NPC43, 8 × 10^3^ cells/100 µL per well; C666-1, 3 × 10^4^ cells/100 µL per well; Raji, 1 × 10^4^ cells/100 µL per well) for 24 h. The growth medium was then replaced by solutions of **ZRL**_**5**_**P**_**2**_, **ZRL**_**5**_**P**_**4**_, or **ZRL**_**5**_**P**_**6**_ at concentrations of 1, 5, 10, 20, 50, and 100 µM for EBV-negative cells or at concentrations of 1, 3, 5, 10, 15, and 20 µM for EBV-positive cells (for the suspensions of C666-1 and Raji cells, the 96-well plate was centrifuged at 1,000 rpm for 3 min before each replacement/withdrawal of medium and operated with care during the assay). After culturing for a further 5 d (half of the volume of medium was replaced every 4 d with fresh medium containing the appropriate drug concentration), the cells were rinsed with PBS and then incubated with a solution of MTT (0.5 mg/mL, 50 µL) in PBS at 37 °C for 3 h. Then, 70% of the medium was carefully removed, DMSO (100 µL) was added, and the plate was shaken for 30 min to solubilize the formazan produced by living cells. The optical densities were measured with a dual-wavelength Labsystem Multiskan microplate reader (Merck Eurolab) at wavelengths of 540 and 690 nm and expressed as a percentage relative to control cells (cells without drug treatment served as the control). Measurements were performed in triplicate and repeated twice. Cell viability (percent) was calculated according to Eq. **1**:

$$\text{Viability}\left(\%\right)=\left({\text{OD}}_{\text{i}}/{\text{OD}}_{\text{c}}\right)\times 100\%,$$ [1]

where OD_i_ and OD_c_ are the optical densities of the surviving cells treated with or without drug, respectively.

### Confocal Microscopy and Costaining.

Cells were incubated with **ZRL**_**5**_**P**_**2**_/**ZRL**_**5**_**P**_**4**_/**ZRL**_**5**_**P**_**6**_ (10 µM) for 3 h and then costained with DRAQ5 (5 µM) for 30 min. Images were acquired using a Leica TCS SP8 confocal laser-scanning microscope equipped with a coherent femtosecond laser (680 to 1,050 nm), argon laser (432, 457, and 488 nm), He-Ne laser (632 nm), ultraviolet lamp, and a controlled CO_2_-content stage-top tissue culture chamber (37 °C, 2 to 7% CO_2_). In vitro images of **ZRL**_**5**_**P**_**2**_/**ZRL**_**5**_**P**_**4**_/**ZRL**_**5**_**P**_**6**_ were obtained under 2-photon excitation (λ_ex_ = 700 nm), whereas images of DRAQ5 were acquired under single-photon excitation (λ_ex_ = 638 nm). The real-time live imaging was performed with a Nikon Eclipse Ti2 confocal laser-scanning microscope.

### Nude Mice Xenograft and Intratumoral Injection.

C666-1 cells (8 × 10^6^) suspended in 100 µL of serum-free RPMI medium were injected into the right flanks of 6- to 8-wk-old male BALB/c nude mice. After 21 d of inoculation, when tumors had grown to an average volume of ∼220 mm^3^, mice were assigned to treatment groups (*n* = 5 per group) such that the average tumor volumes varied between groups by no more than 10%. Twice weekly, mice received 4 µg per tumor intratumoural injections of **ZRL**_**5**_**P**_**2**_, **ZRL**_**5**_**P**_**4**_, or **ZRL**_**5**_**P**_**6**_ in 0.1% DMSO using a 29-gauge syringe. Mice that received an equivalent volume of 0.1% DMSO alone served as controls. The treatment period lasted for 18 d. Body weight and tumor volumes were measured twice weekly, and tumor volumes were calculated as (length × width^2^)/2. At the end of the treatment period, mice were killed and their tumors and major organs were harvested and weighed. The investigators were blinded to treatment grouping during the experiments and analysis of data. All animal experiments were approved by the Department of Health of the Hong Kong Government and the Animal Subjects Ethics Sub-Committee of Hong Kong Polytechnic University. No power analyses were used to calculate the sample size for the animal studies.

### Quantitative Analysis of Cell-Free Plasma EBV DNA.

The plasma EBV DNA was detected as previously described (35). In brief, the plasma DNA was extracted using the QIAamp DNA Blood mini kit. The PCR primers and probe and the reaction condition strictly followed the same study. The EBV-positive cell line Namalwa was used as a standard, and the EBV copy number was calculated.

### qPCR Gene Expression Analysis.

qPCR was performed as reported (36). The primers used in this study were described in a previous publication (36, 37).

### IF Staining.

IF staining was performed as previously described (36). Primary antibodies against Zta were supplied from Argene (Verniolle). Antibodies against BMRF1, gp350/220, and VCA-p18 were generated by J.M.M. These antibodies were produced and characterized as previously described (38–40). The Dicer1 and PML antibodies were supplied by Abcam and Bethyl Laboratories, respectively. Images were captured by the Eclipse Ti System (Nikon).

### Western Blot Analysis.

Western blot analysis of Zta, Rta, BMRF1, and gp350/220 was performed as reported (41). The Rta antibody was supplied from Argene (Verniolle) and the β-actin antibody for loading control was supplied from Cell Signaling Technology. Antibodies against Zta, BMRF1, gp350/220, Dicer1, and PML were the ones used for IF staining. A 1:400 dilution of the primary antibodies was used.

### Preparation of Formalin-Fixed, Paraffin-Embedded Tumor Tissues, H&E Stain, and IHC.

The transplanted tumor tissues were fixed with formalin and embedded in paraffin accordingly to the general practice. The histologic sections were prepared and stained with H&E. The lytic protein markers were stained with IHC antibodies against Zta and BMRF1. The Zta and the BMRF1 antibody were the ones used for Western blot analysis. The slides were incubated with the primary antibodies (1:100 dilution) for IHC as previously described (41).

### EBV Infection Assay.

The HONE-1-EBV cell line was used to produce the infectious EBV particles for the lytic analysis; this cell line was generated by introducing a GFP open reading frame in the recombinant Akata EBV genome into the EBV-negative NPC cell line HONE-1 (42). The procedures of production of viral particles and quantitation of virus titers were followed as previously described (43, 44). In brief, after incubation with various EBNA1 probes for 96 h, the supernatants were filtered through 0.45-µm-pore filters and the viral particles were enriched by centrifugation at 20,000 × *g* for 2 h. The relative virus titers were determined by the Raji cell assay and were quantified with the GFP expression of Raji cells infected with the virus stocks to be analyzed. The Raji cells (1 × 10^5^) were incubated in 96-well plates and cultivated for 3 d at 37 °C to allow the expression of GFP. The number of GFP-positive cells was counted by ultraviolet microscopy.

## Supplementary Material

Supplementary File
