# Supplementary material for: Reactivation of Epstein–Barr virus by a dual-responsive fluorescent EBNA1-targeting agent with Zn2+-chelating function
Source: Proc Natl Acad Sci U S A. 2019 Dec 10;116(52):26614–24. doi: 10.1073/pnas.1915372116 (PMC6936348; doi:10.1073/pnas.1915372116)
Supplement: Supplementary File [file pnas.1915372116.sapp.pdf]

PNAS
www.pnas.org

Supplementary Information for

Reactivation of Epstein Barr virus by a dual-responsive fluorescent EBNA1-targeting agent with Zn^{2+}-chelating function

Lijun Jiang[a,b,#], Hong Lok Lung[c,#,*], Tao Huang[f,#], Rongfeng Lan[d,#], Shuai Zha[a], Lai Sheung Chan[c], Waygen Thor[a], Tik-Hung Tsoi[e], Ho-Fai Chau[a], Cecilia Boreström[h], Steven Cobb[i], Sai Wah Tsao[g], Zhao-Xiang Bian[f], Ga-Lai Law[e,*], Wing-Tak Wong[e], William Chi-Shing Tai[e], Wai Yin Chau[c], Yujun Du[c], Lucas Hao Xi Tang[c], Alan K.S. Chiang[j], Jaap M. Middeldorp[k], Kwok-Wai Lo[l], Nai-Ki Mak[c,*], Nicholas J. Long[b,*], Ka-Leung Wong[a,*]

Hong Lok Lung, Ga-Lai Law, Nai-Ki Mak, Nicholas J. Long, Ka-Leung Wong
Email: hllung2@hkbu.edu.hk, ga-lai.law@polyu.edu.hk, nkmak@hkbu.edu.hk, n.long@imperial.ac.uk, klwong@hkbu.edu.hk

This PDF file includes:

Supplementary text
Figures S1 to S31
Tables S1 to S5
Scheme S1
SI References

www.pnas.org/cgi/doi/10.1073/pnas.1915372116

Molecular dynamics (MD) simulations

Modelling and simulations of the P_4-EBNA1 complex. **P_4** (YFMVFGGRrRK) was docked into the dimeric interface of the EBNA1 DNA-binding domain (DBD) monomer which was generated in a previous study (1) with the online CABS-dock tool (2). Ten **P_4**-EBNA1 complex models were generated and subjected to constant pressure and temperature (NPT) simulations in AMBER 14 with CUDA acceleration (3, 4). By observing the fluctuation of **P_4** in each complex, the most stable complex model was chosen and subjected to an additional 500 ns NPT simulation. The results showed that **P_4** stably occupied the dimeric interface of the putative EBNA1 DBD monomer, suggesting that it is a strong EBNA1 dimeric interface binder (Fig. S1). The representative **P_4**-EBNA1 DBD monomer complex structure was obtained from the conformational clustering analysis (5). In line with the previous report (1), the hydrophobic packing effect mediated by YFMVF and the salt bridge formed with RrRK contributed to the interactions between **P_4** and EBNA1 DBD monomer (Fig. S2).

Modelling of the Zn^{2+}-ZRL_5 complex. The 3D models of **ZRL_5** in both Zn^{2+}-free and Zn^{2+}-coordinated scenarios were built using the Molecular Operation Environment (MOE, version 2012.10) (6). The MMFF94x force field was used to minimize the energy. In addition, LowModeMD was used to identify the global energy minimum conformation (Fig. S3 A–D). The partial charges of **ZRL_5** and Zn^{2+}-**ZRL_5** were calculated using the online RED server (7), and their atom types were manually determined by output from the parmchk tool (8) (Fig. S5). In addition, the missing force field parameters of **ZRL_5** and Zn^{2+}- **ZRL_5** were determined from comparable atoms in GAFF or measurements of RED-derived results (Tables S1 and S2).

Modelling and simulations of ZRL_5P_4-EBNA1 and Zn^{2+}-ZRL_5P_4-EBNA1 complexes. The initial structures of **ZRL_5P_4**-EBNA1 and Zn^{2+}- **ZRL_5P_4**-EBNA1 complexes were built based on the structure of **P_4**-EBNA1 complex and the putative Zn^{2+}-**ZRL_5**, with CAhx serving as the linker. The structures were then arranged for 200 ns MD simulations.

Post-simulation analysis. Ten conformational clusters of **P_4**-EBNA1 DBD complex were calculated with cpptraj by using the default settings with the distance defined by the root-mean-square deviation (RMSD) of the main chain atoms. The representative conformation of the most abundant cluster (cluster 0) was saved for **P_4**-EBNA1 DBD interaction analysis. The free energies of binding for the whole production trajectory were calculated by MMPBSA (9). The time intervals were adjusted to ensure 200 frames were included in the calculation. mbondi2 was used, and the salt concentration was set to 0.1 M in the generalized Born (GB) calculation; the radii from the prmtop file was used and the ionic strength was set to 0.1 mM for the Poisson-Boltzman (PB) calculation.

Synthesis

Experimental procedures.

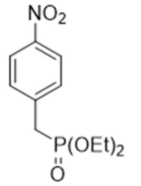

1

Synthesis of diethyl 4-nitrobenzylphosphonate (1). Compound **1** was prepared according to a previous report (11). A mixture of 4-nitrobenzyl bromide (200 mg, 0.93 mmol) and triethylphosphite (216 mg, 1.30 mmol) was heated at 160 °C under a nitrogen atmosphere for 2 hrs. Excess triethylphosphite was removed under reduced pressure to provide 1 as a brown oil (241mg, 95%). ^1H NMR (400 MHz, CDCl3): δ 8.19 (dd, J = 8.8, 0.8 Hz, 2H), 7.48 (dd, J = 8.8, 2.4 Hz, 2H), 4.07 (dq, J = 7.6, 0.4 Hz, 2H), 4.05 (dq, J = 7.6, 0.4 Hz, 2H), 3.25 (d, J = 22.4 Hz, 2H), 1.27 ppm (t, J = 6.8 Hz, 6H); ^{13}C NMR (100 MHz, CDCl3): δ 139.7 (d, 2J(C,P) = 8.9 Hz), 130.6 (d, 3J(C,P) = 6.4 Hz), 123.7 (d, 4J(C,P) = 2.9 Hz), 62.4 (d, 2J(C,P) = 6.7 Hz), 34.0 (d, 1J(C,P) = 136.7 Hz), 16.4 ppm

(d, 3J(C,P) = 5.9 Hz); HRMS (MALDI-TOF): m/z: calcd for $C_{11}H_{16}NO_5P$ 273.0766 $[M]^+$; found: 273.0755.

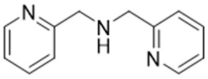
2

Synthesis of di-(2-picolyl)amine (2). Compound **2** was prepared according to a previous report (12). A solution of 2-picolylamine (5.0 g, 46.24 mmol) in absolute EtOH (100 ml) was added dropwise to a suspension of 2-pyridinecarboxaldehyde (4.9 g, 46.05 mmol) in absolute EtOH (100 ml) at 0 °C. After the addition, the reaction solution was stirred for 4 hrs and then cooled to 0 °C. $NaBH_4$ (3.5 g, 92.10 mmol) was added to the cooled solution in small portions. The reaction was stirred for another 12 hrs at room temperature. Then, HCl (5 N, 120 ml) was added slowly and the mixture was stirred for 1 hr. Aqueous NaOH (2 N) was then added until the pH reached 11. The mixture was extracted with DCM (6 × 50 ml), and the combined organic phases were dried over anhydrous $MgSO_4$ and filtered. Removal of the solvent afforded **2** in analytical purity as a brown oil (7.8 g, 85%). ^1H NMR (400 MHz, CD_3OD): δ 8.49 (qd, J = 4.8, 0.8 Hz, 2H), 7.81 (dt, J_1 = 7.6, 1.6 Hz, 2H), 7.51 (d, J = 8.0 Hz, 2H), 7.31 (m, 2H), 3.93 ppm (s, 4H); ^{13}C NMR (100 MHz, CD_3OD): δ 160.3, 149.8, 138.7, 124.1, 123.8, 54.8 ppm; HRMS (MALDI-TOF): m/z: calcd for $C_{12}H_{14}N_3$ 200.1188 $[M+H]^+$; found: 200.1326.

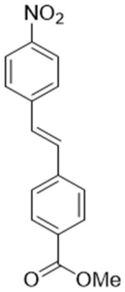
3

Synthesis of (E)-methyl 4-(4-nitrostyryl)benzoate (3). Compound **3** was prepared according to a previous report (13). A solution of diethyl 4-nitrobenzylphosphonate (200 mg, 0.73 mmol) and methyl 4-formylbenzoate (120 mg, 0.73 mmol) in THF (3 ml) was added to a suspension of sodium methoxide (79 mg, 1.46 mmol) in THF (6 ml). The mixed solution was stirred for 20 min at 0 °C. The solvent was removed under reduced pressure. H_2O (100 ml) was added, and the resulting aqueous solution was neutralized with HCl (0.1 N), followed by extraction with DCM (3 × 50 ml). The combined organic phases were dried over anhydrous $MgSO_4$ and filtered. Then, the solvents were removed under reduced pressure. The pure product **3** (yellow solid) was obtained by filtering the crude material through silica gel with DCM as the eluent (182 mg, 88%). ^1H NMR (400 MHz, $CDCl_3$): δ 8.25 (dd, J_1 = 6.8, 2 Hz, 2H), 8.07 (dd, J_1 = 6.8, 1.6 Hz, 2H), 7.67 (dd, J_1 = 7.2, 2 Hz, 2H), 7.61 (d, J = 8.4 Hz, 2H), 7.28-7.26 (2H, coincided with residual $CHCl_3$), 3.94 ppm (s, 3H); ^{13}C NMR (100 MHz, $CDCl_3$): δ 166.7, 147.2, 143.2, 140.5, 132.1, 130.2, 130.0, 128.7, 127.2, 126.9, 124.2, 52.2 ppm; HRMS (MALDI-TOF): m/z: calcd for $C_{16}H_{14}NO_4$ 284.0923 $[M+H]^+$; found: 284.0895.

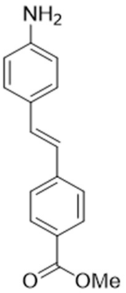
4

Synthesis of (E)-methyl 4-(4-aminostyryl)benzoate (4). Compound **4** was prepared according to a previous report (14). A solution of 3 (200 mg, 0.71 mmol) in EtOAc (10 ml) was stirred under a

nitrogen atmosphere at 75 °C. Stannous chloride dihydrate (801 mg, 3.55 mmol) was added and the mixture was heated at 75 °C and stirred overnight. After the reaction was complete (determined by TLC, thin-layer chromatography), the mixture was allowed to cool to room temperature, followed by the addition of aqueous $NaHCO_3$ until the pH reached 8. The suspension was filtered through a pad of celite and washed with EtOAc. The filtrate was washed with H_2O (twice) and brine, then dried over anhydrous $MgSO_4$ and filtered and concentrated under reduced pressure to afford **4** as an orange-yellow solid (144 mg, 80%). ^{1}H NMR (400 MHz, $CDCl_3$): δ 7.99 (d, J = 8.0 Hz, 2H), 7.51 (d, J = 8.4 Hz, 2H), 7.36 (d, J = 8.4 Hz, 2H), 7.13 (d, J = 16.4 Hz, 1H), 6.93 (d, J = 16.4 Hz, 1H), 6.69 (d, J = 8.4 Hz, 2H), 3.92 ppm (s, 3H); ^{13}C NMR (100 MHz, $CDCl_3$): δ 167.0, 146.7, 142.6, 131.3, 130.0, 128.2, 128.1, 127.4, 125.8, 123.8, 115.2, 52.0 ppm. HRMS (MALDI-TOF): m/z: calcd for $C_{16}H_{15}NO_2$ 253.1103 $[M]^+$; found: 253.1084.

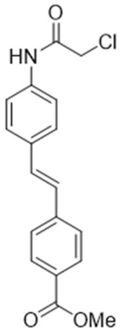

5

Synthesis of (E)-methyl 4-[4-(2-chloroacetamido)styryl]benzoate (5) (10). A solution of 2-chloroacetyl chloride (53 mg, 0.47 mmol) in DCM (3 ml) was added dropwise to a solution of 4 (100 mg, 0.39 mmol) and 4-(dimethylamino)pyridine (81 mg, 0.66 mmol) in DCM (15 ml) at 0 °C and stirred under a nitrogen atmosphere. After stirring for 2 hrs at room temperature, the solvent was removed under reduced pressure. The obtained pale-yellow solid was purified by silica gel with DCM as the eluent to give **5** as an orange solid (109 mg, 85%). ^{1}H NMR (400 MHz, $CDCl_3$): δ 8.28 (s, 1H), 8.03 (d, J = 8.4 Hz, 2H), 7.60-7.53 (m, 6H), 7.19 (d, J = 16.4 Hz, 1H), 7.09 (d, J = 16.4 Hz, 1H), 4.22 (s, 2H), 3.93 ppm (S, 3H); ^{13}C NMR (100 MHz, $CDCl_3$): δ 167.8, 163.7, 141.7, 136.5, 133.7, 130.2, 130.0, 128.9, 127.5, 127.3, 126.2, 120.1, 52.1, 42.9 ppm; HRMS (MALDI-TOF): m/z: calcd for $C_{18}H_{16}ClNO_3$ 329.0819 $[M]^+$; found: 329.0807.

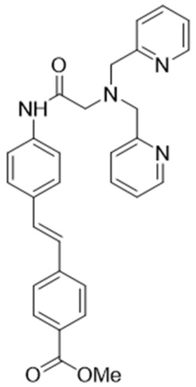

6

Synthesis of (E)-methyl 4-(4-{2-[bis(pyridine-2-ylmethyl)amino]acetamido}styryl)benzoate (6) (10). Compound **5** (50 mg, 0.15 mmol), di(2-picolyl)amine (DPA, 36 mg, 0.18 mmol), N,N-diisopropylethylamine (DIPEA, 33 mg, 0.26 mmol), and potassium iodide (16 mg, 0.67 mmol) were added to MeCN (25 ml). After stirring and heating at reflux overnight under a nitrogen atmosphere, the mixture was allowed to cool to room temperature, and the solvent was removed under reduced pressure to give a yellow-brown solid. The crude material was then purified by silica gel column chromatography using DCM/MeOH (100:1; the DCM was extracted with aqueous NH_3 before use, DCM/NH_3 200:1 v/v) to obtain **6** (59 mg, 80%). ^{1}H NMR (400 MHz, $CDCl_3$): δ 11.04 (s, 1H), 8.64

(qd, J_1 = 4.8, 0.8 Hz, 2H), 8.02 (d, J = 8.4 Hz, 2H), 7.82 (d, J = 8.4 Hz, 2H), 7.64 (dt, J = 7.6, 2.0 Hz, 2H,), 7.56 (d, J = 8 Hz, 2H), 7.53 (d, J = 8.4 Hz, 2H), 7.29 (d, J = 7.6 Hz, 2H), 7.22-7.18 (m, 3H), 7.07 (d, J = 16.4 Hz, 1H), 3.95 (s, 4H), 3.93 (s, 3H), 3.49 ppm (s, 2H); ^{13}C NMR (100 MHz, $CDCl_3$): δ 169.8, 166.9, 158.0, 149.4, 142.0, 138.7, 136.7, 132.2, 130.8, 130.0, 127.4, 126.2, 126.1, 123.3, 122.6, 119.8, 60.4, 58.7, 52.1 ppm; HRMS (MALDI-TOF): m/z: calcd for $C_{30}H_{28}N_4NaO_3$ 515.2059 $[M+Na]^+$; found: 515.2654.

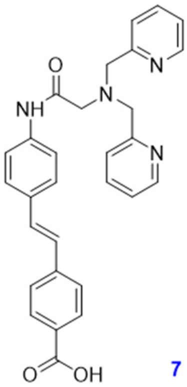

7

Synthesis of ZRL$_5$ (7) (14). KOH (114 mg, 2.03 mmol) was added to a suspension of 6 (200 mg, 0.41 mmol) in EtOH/H2O (1:1, 8 ml). After stirring and heating at reflux overnight, the reaction mixture was allowed to cool to room temperature. The solvent volume was reduced to approximately 50%, and then HCl (1 N) was added until the solution became acidic. The resulting precipitate was filtered and collected to give **7** as a yellow solid (157 mg, 78%). ^{1}H NMR (400 MHz, $CDCl_3$): δ 10.74 (s, 1H), 8.68 (d, J = 4.4 Hz, 2H), 8.00 (d, J = 8.4 Hz, 2H), 7.73 (d, J = 8.4 Hz, 2H), 7.67 (dt, J = 7.6, 1.6 Hz, 2H), 7.51 (d, J = 8.4 Hz, 2H), 7.43 (d, J = 8.8 Hz, 2H), 7.33 (d, J = 7.6 Hz, 2H), 7.26-7.22 ppm (m, 2H), 7.16 (d, J = 16.4 Hz, 1H), 7.02 (d, J = 16.4 Hz, 1H), 4.02 (s, 4H), 3.54 ppm (s, 2H); ^{13}C NMR (100 MHz, $CDCl_3$): δ 169.4, 157.4, 149.1, 142.4, 138.5, 137.2, 132.4, 130.9, 130.5, 127.3, 126.3, 126.1, 123.7, 122.9, 120.1, 60.3, 59.0 ppm; HRMS (MALDI-TOF): m/z: calcd for $C_{29}H_{26}N_4NaO_3$ 501.1903 $[M+Na]^+$; found: 501.1902.

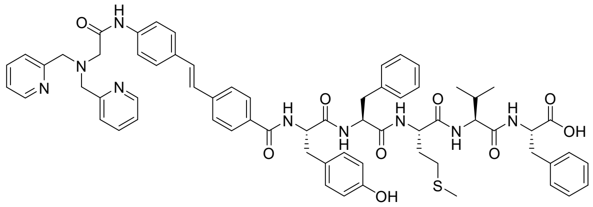

ZRL$_5$P$_2$

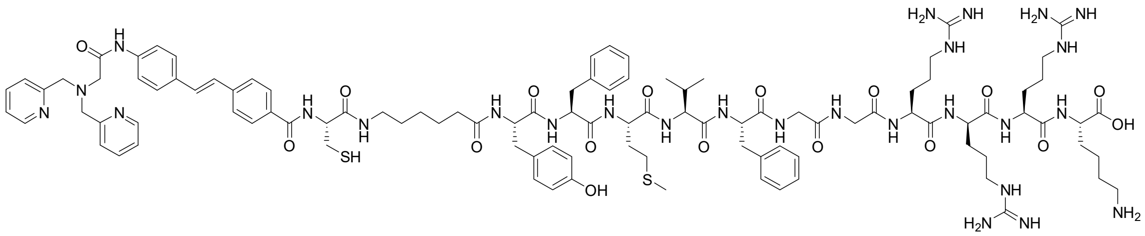

ZRL$_5$P$_4$

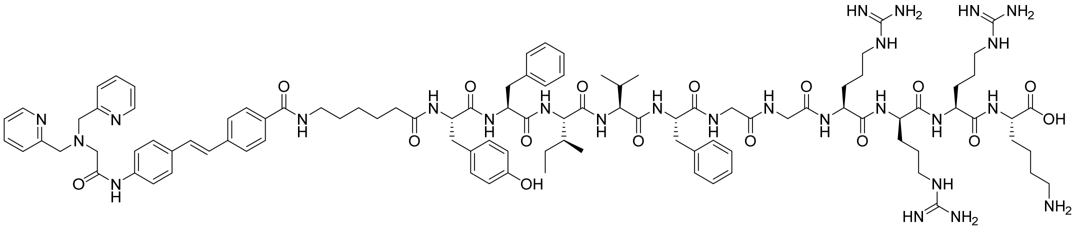

ZRL_5P_6

Synthesis of ZRL_5P_2/ZRL_5P_4/ZRL_5P_6.

Conjugation. A solution of **7** (148 mg, 0.30 mmol) and (benzotriazol-1-yloxy)tripyrrolidinophosphonium hexafluorophosphate (PyBOP, 156 mg, 0.3 mmol) was dissolved in DMF (3 ml). DIPEA (90 mg, 0.70 mmol) was added and the solution was stirred for 1 min. The solution was added to the appropriate peptide (**P_2**/ **P_4**/ **P_6**, 0.1 mmol) loaded onto Wang resin on an 8 ml solid-phase extraction (SPE) cartridge with PE frits. The peptide resin was allowed to swell in DMF overnight, and the chamber was drained before addition. The resulting mixture was agitated on a shaker for 45 min, and the solution was then filtered and washed with DMF three times. A second round of coupling was performed according to the same procedures, and a blank coupling cycle with PyBOP (156 mg, 0.3 mmol) and DIPEA (90 mg, 0.70 mmol) in DMF (3 mL) was performed. After 45 min, the solution was filtered and washed as described above.

Resin Cleavage. A mixture of TFA/H_2O/TIPS (0.95:0.25:0.25; TFA, trifluoroacetic acid; TIPS, triisopropylsilane) was added to the crude resin shrunken with diethyl ether. The mixture was agitated on a shaker for 3 hrs. The resin was then removed by filtration and washed with MeOH three times. The combined filtrate was then concentrated under reduced pressure to give the crude **ZRL_5P_2**/**ZRL_5P_4**/**ZRL_5P_6**, which were then purified by preparative HPLC.

Purification via HPLC. The crude **ZRL_5P_2**, **ZRL_5P_4** and **ZRL_5P_6** were then purified using a Waters semi-preparative High-Performance Liquid Chromatography (HPLC) system equipped with a Waters C18 column (250 × 19 mm). The purification process was detected under wavelength 220, 254, 280, 340, 360, and 380 nm. Mobile phase A was deionised water containing 0.05 % trifluoroacetic acid (TFA); mobile phase B was HPLC grade acetonitrile (MeCN). The method used for purification is shown below (Table S4). Identity of the target peak was confirmed with either Bruker Autoflex Matrix Assistant Laser Desorption Ionization iV Time of Flight Mass Spectrometer (MALDI-TOF MS) or Liquid Chromatography-Electrospray Ionization-Quadrupole-Time of Flight Mass Spectrometry (LC-ESI-Q TOF) operating in the positive ion mode.

ZRL_5P_2. Retention time, 24.061 min. HRMS(Q-TOF): calcd for $[M+H]^+$ 1166.5174, found: 1166.5195; calcd for [M+2H]/2 583.7626, found: 583.7619.

ZRL_5P_4. Retention time, 16.996 min. HRMS (MALDI-TOF): calcd for $[M+H]^+$ 2094.0552, found: 2094.2452.

ZRL_5P_6. Retention time, 17.319 min. HRMS (Q-TOF): calcd for [M+2H]/2 987.0487, found: 987.0501; calcd for [M+3H]/3 658.3684, found: 658.3686; calcd for [M+4H]/4 494.0282, found: 494.0280.

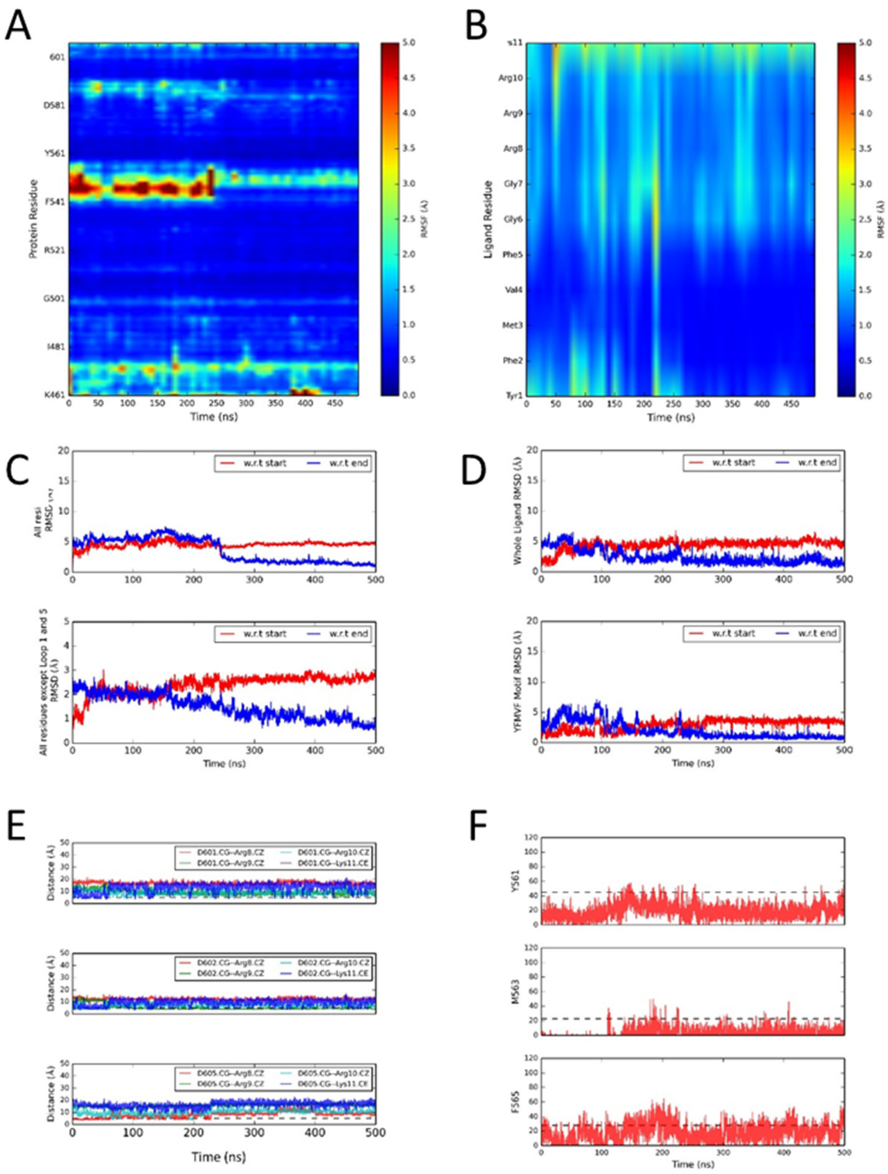

Fig. S1. 500 ns NPT simulation of the **P$_4$**-EBNA1 complex model (#9); the complex model was chosen using flexible peptide docking. (A) Root-mean-square fluctuation (RMSF) of the putative EBNA1 DBD monomer. (B) RMSF of **P$_4$**. (C) RMSD of the main-chain atoms of EBNA1 DBD from the initial (red) and end (blue) structures of the complex. (D) RMSD of the main-chain atoms of **P$_4$** from the initial (red) and end (blue) structures of the complex. (E) The salt-bridging between the RrRK motif and the acidic residues in the C-terminal EBNA1 DBD. (F) The hydrophobic packing between the YFMVF motif and the dimeric interface of the EBNA1 DBD.

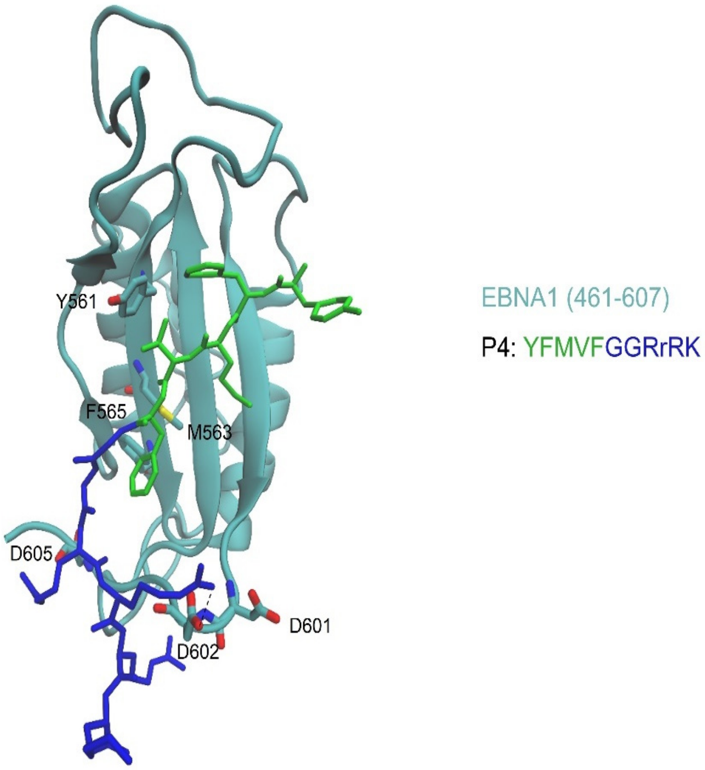

Fig. S2. Representative **P$_4$**-EBNA1 complex structure after 500 ns MD simulations. The EBNA1 DBD monomer, cyan; YFMVF motif in **P$_4$**, green; RrRK motif in **P$_4$**, blue. **P$_4$**-EBNA1 DBD binding was mediated both by hydrophobic interactions between YFMVF and the dimeric interface of EBNA1 DBD and by salt bridges between the RrRK motif and the C-terminus of EBNA1 DBD.

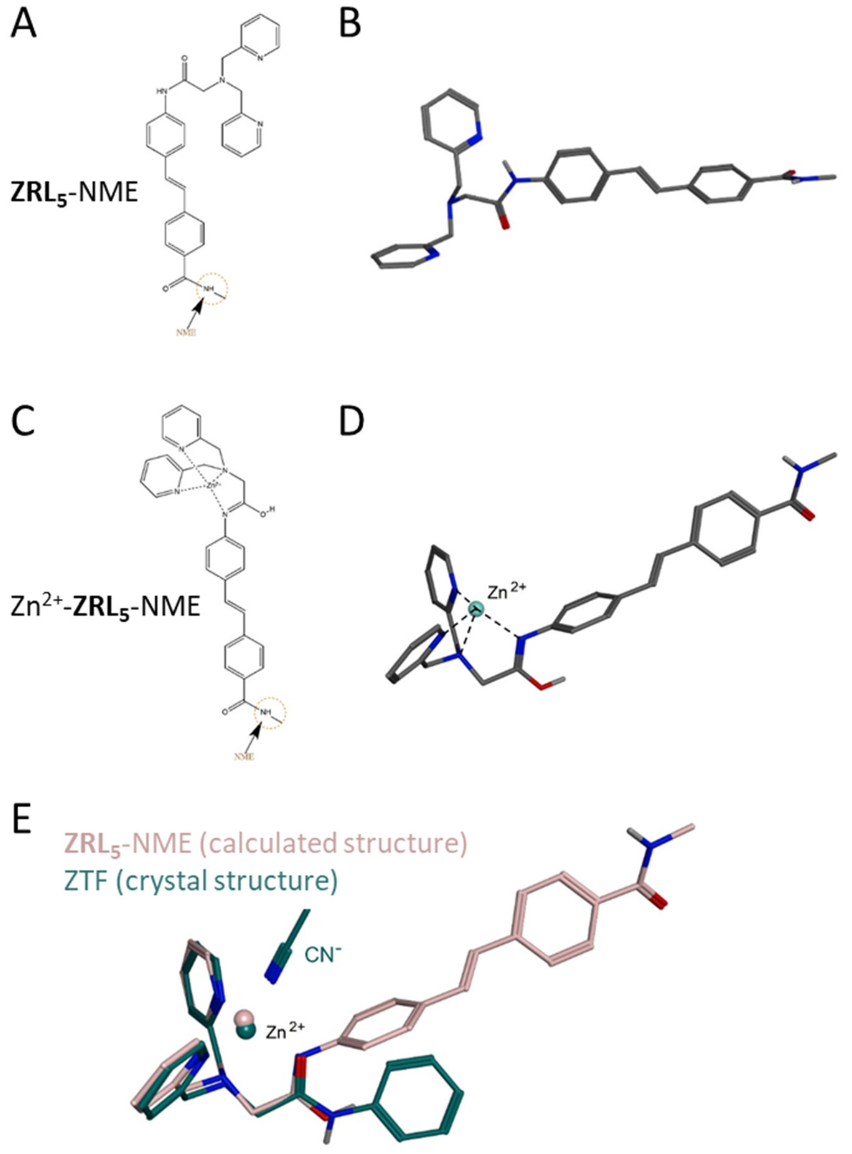

Fig. S3. Putative 3D structures of **ZRL$_5$**-NME and Zn^{2+}-**ZRL$_5$**-NME from the molecular mechanics calculation. (A) 2D structure of **ZRL$_5$**-NME. (B) Calculated 3D structure of **ZRL$_5$**-NME. (C) 2D structure of Zn^{2+}-**ZRL$_5$**-NME. (D) Calculated 3D structure of Zn^{2+}-**ZRL$_5$**-NME. (E) Superposition of the calculated Zn^{2+}-**ZRL$_5$**-NME 3D structure (pink) onto the crystal structure of ZTF-Zn^{2+} (green), where ZTF-Zn^{2+} is a highly similar compound that has been reported previously (10).

A

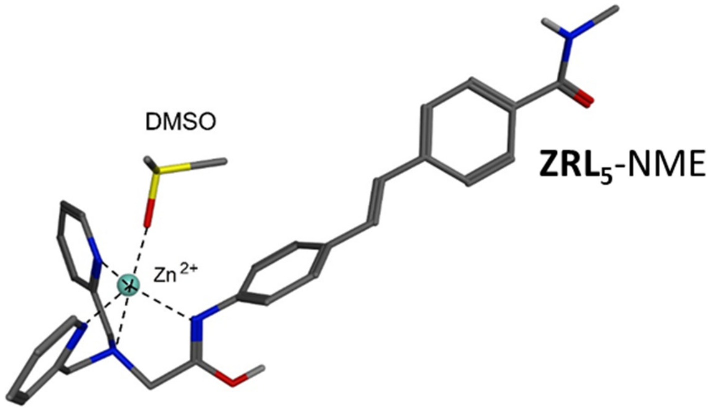

B

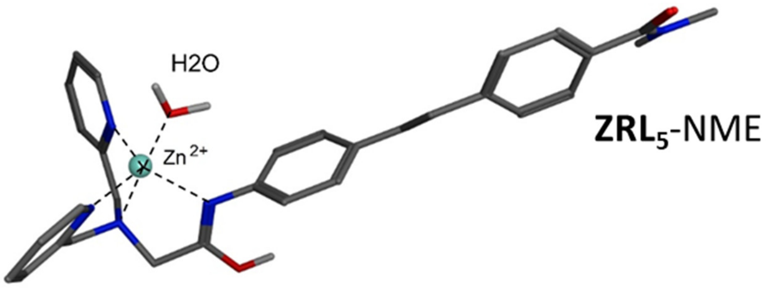

Fig. S4. Putative structures of the Zn^{2+}-**ZRL_5**-NME complex with 5-coordinated Zn^{2+}, where a solvent molecule (DMSO or H_2O) occupies the fifth coordination site. (A) Calculated 3D structure of DMSO-Zn^{2+}-ZRL_5-NME. (B) Calculated 3D structure of H_2O-Zn^{2+}-**ZRL_5**-NME.

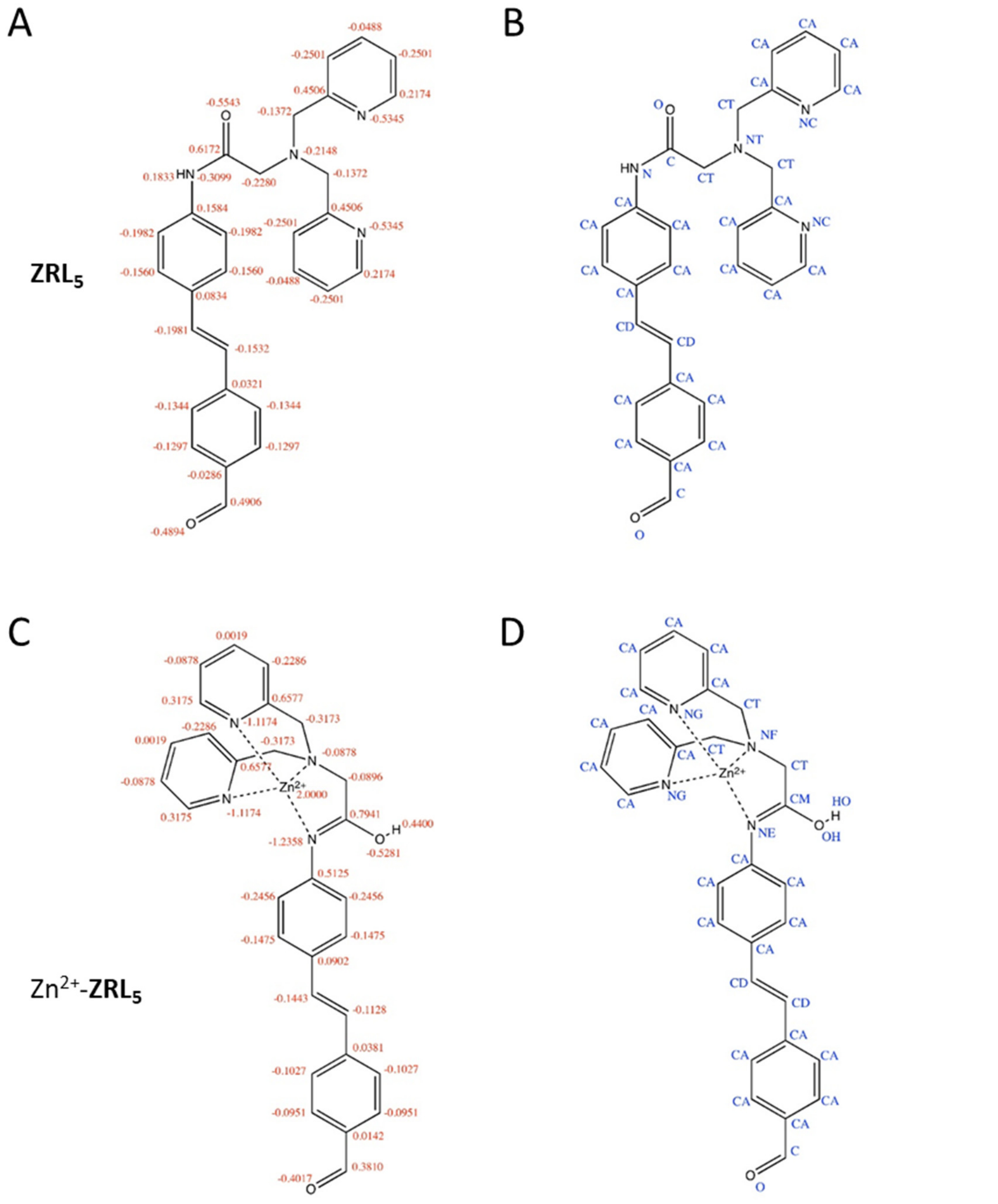

Fig. S5. Partial charge (A and C) and AMBER atom types (B and D) of **ZRL$_5$** (A and B) and Zn^{2+}-**ZRL$_5$** (C and D) for MD simulations.

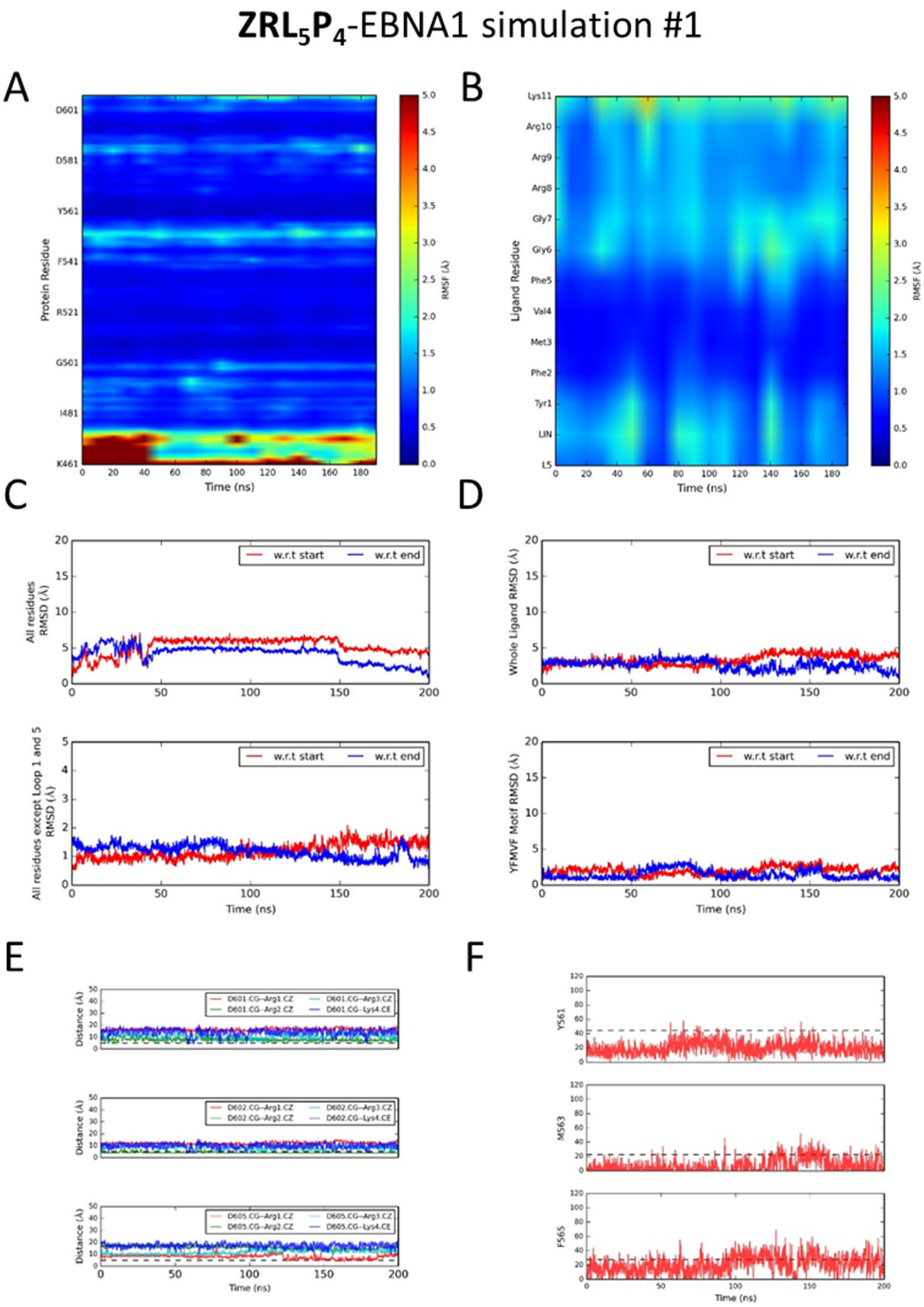

Fig. S6. 200 ns NPT simulation of the **ZRL₅P₄**-EBNA1 complex model (#1). (A) RMSF of the putative EBNA1 DBD monomer. (B) RMSF of **ZRL₅P₄**. (C) RMSD of the main-chain atoms of EBNA1 DBD from the initial (red) and end (blue) structures of the complex. (D) RMSD of main-chain atoms of **ZRL₅P₄** ligand from the initial (red) and end (blue) structures of the complex. (E) The salt-bridging between the RrRK motif and the acidic residues in the C-terminus of the EBNA1 DBD. (F) The hydrophobic interactions between the YFMVF motif and the dimeric interface of the EBNA1 DBD.

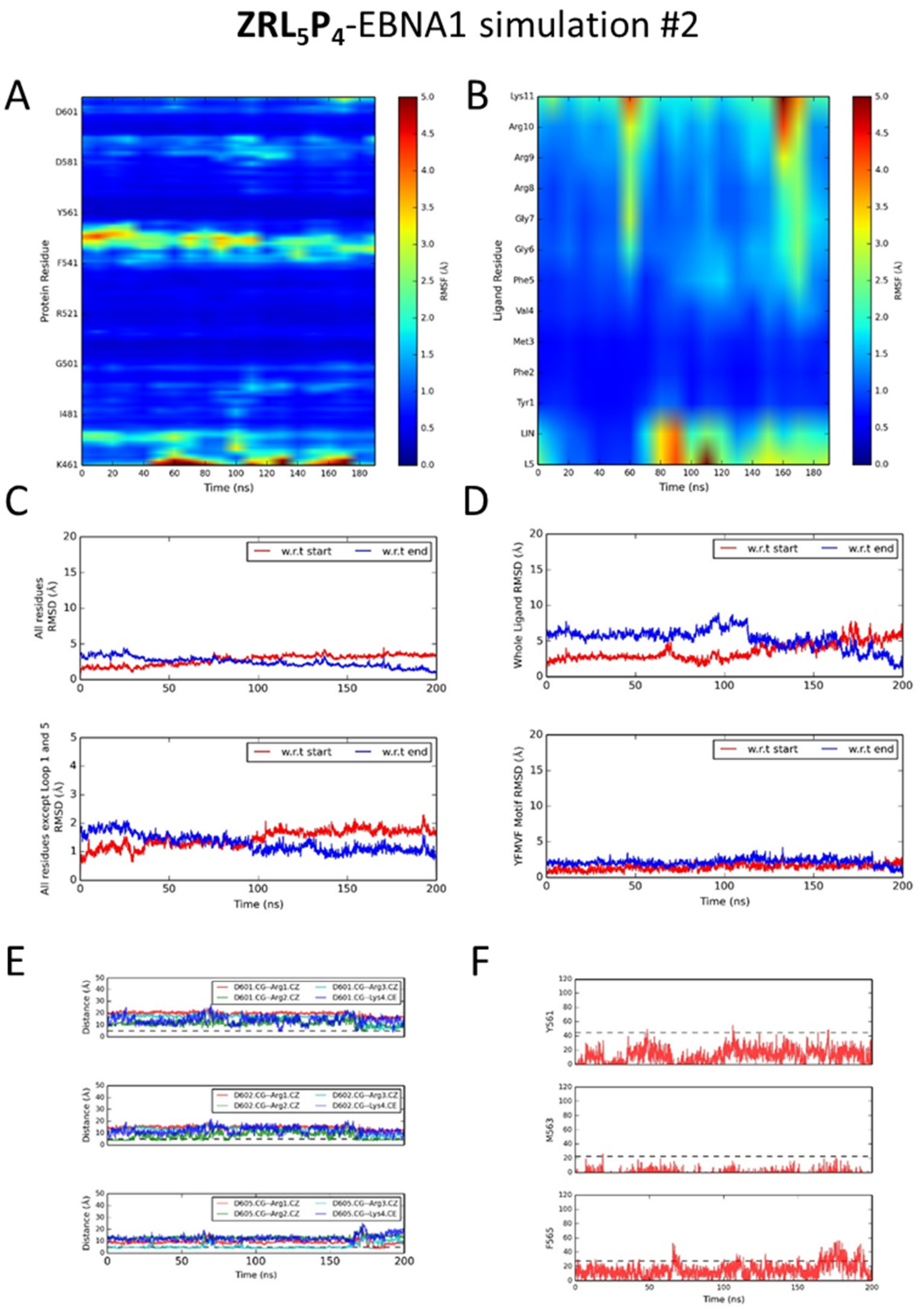

Fig. S7. 200 ns NPT simulation of the **ZRL$_5$P$_4$**-EBNA1 complex model (#2). (A) RMSF of the putative EBNA1 DBD monomer. (B) RMSF of **ZRL$_5$P$_4$**. (C) RMSD of the main-chain atoms of EBNA1 DBD from the initial (red) and end (blue) structures of the complex. (D) RMSD of the main-chain atoms of **ZRL$_5$P$_4$** from the initial (red) and end (blue) structures of the complex. (E) The salt-bridging between the RrRK motif and the acidic residues in the C-terminus of the EBNA1 DBD. (F) The hydrophobic interactions between the YFMVF motif and the dimeric interface of the EBNA1 DBD.

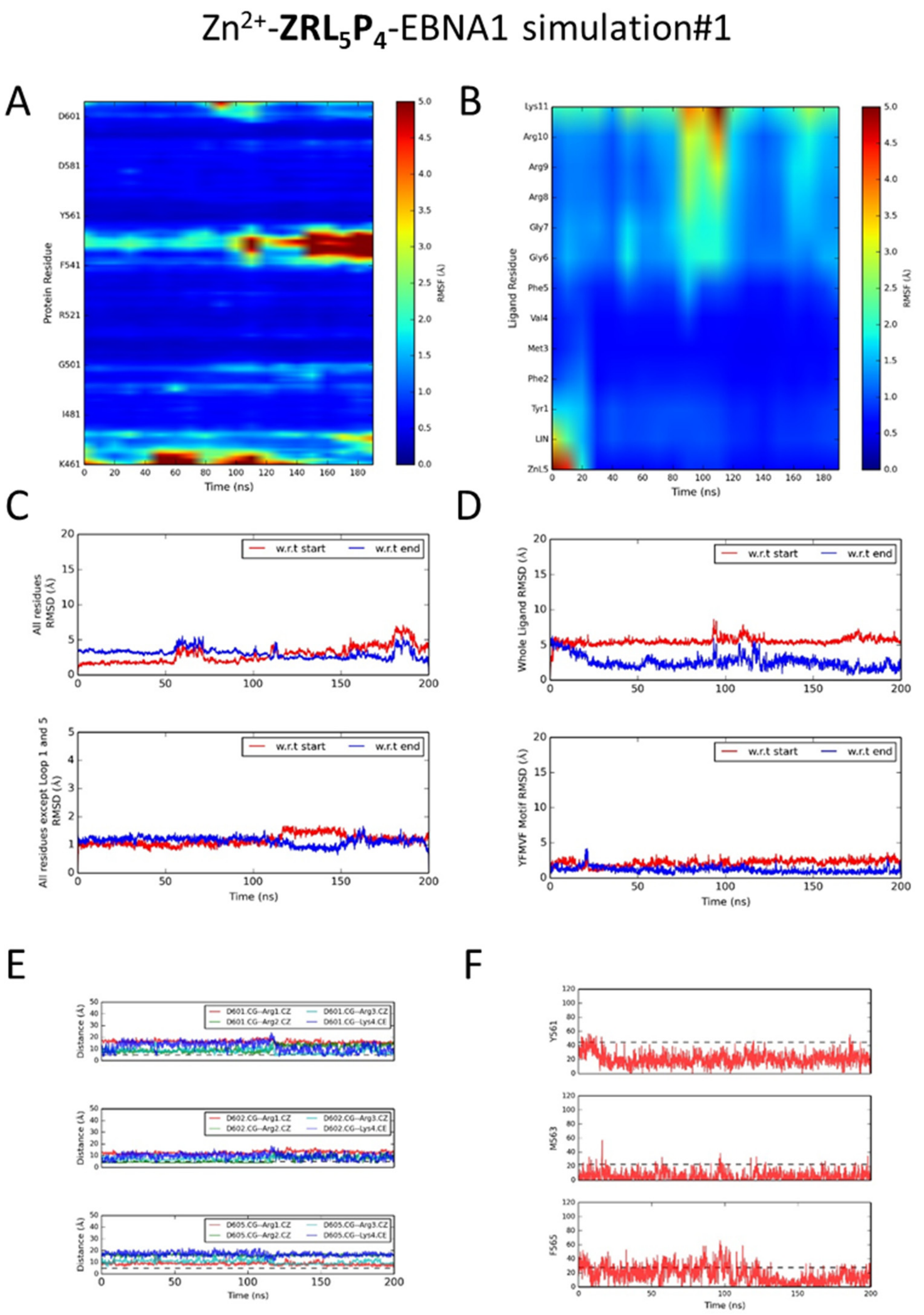

Fig. S8. 200 ns NPT simulation of the Zn^{2+}-**ZRL$_5$P$_4$**-EBNA1 complex model (#1). (A) RMSF of the putative EBNA1 DBD monomer. (B) RMSF of Zn^{2+}- **RL$_5$P$_4$**. (C) RMSD of the main-chain atoms of EBNA1 protein from the initial (red) and end (blue) structures of the complex. (D) RMSD of the main-chain atoms of Zn^{2+}-**ZRL$_5$P$_4$** ligand from the initial (red) and end (blue) structures of the complex. (E) The salt-bridging between the RrRK motif and the acidic residues in the C-terminus of the EBNA1 DBD. (F) The hydrophobic interactions between the YFMVF motif and the dimeric interface of the EBNA1 DBD.

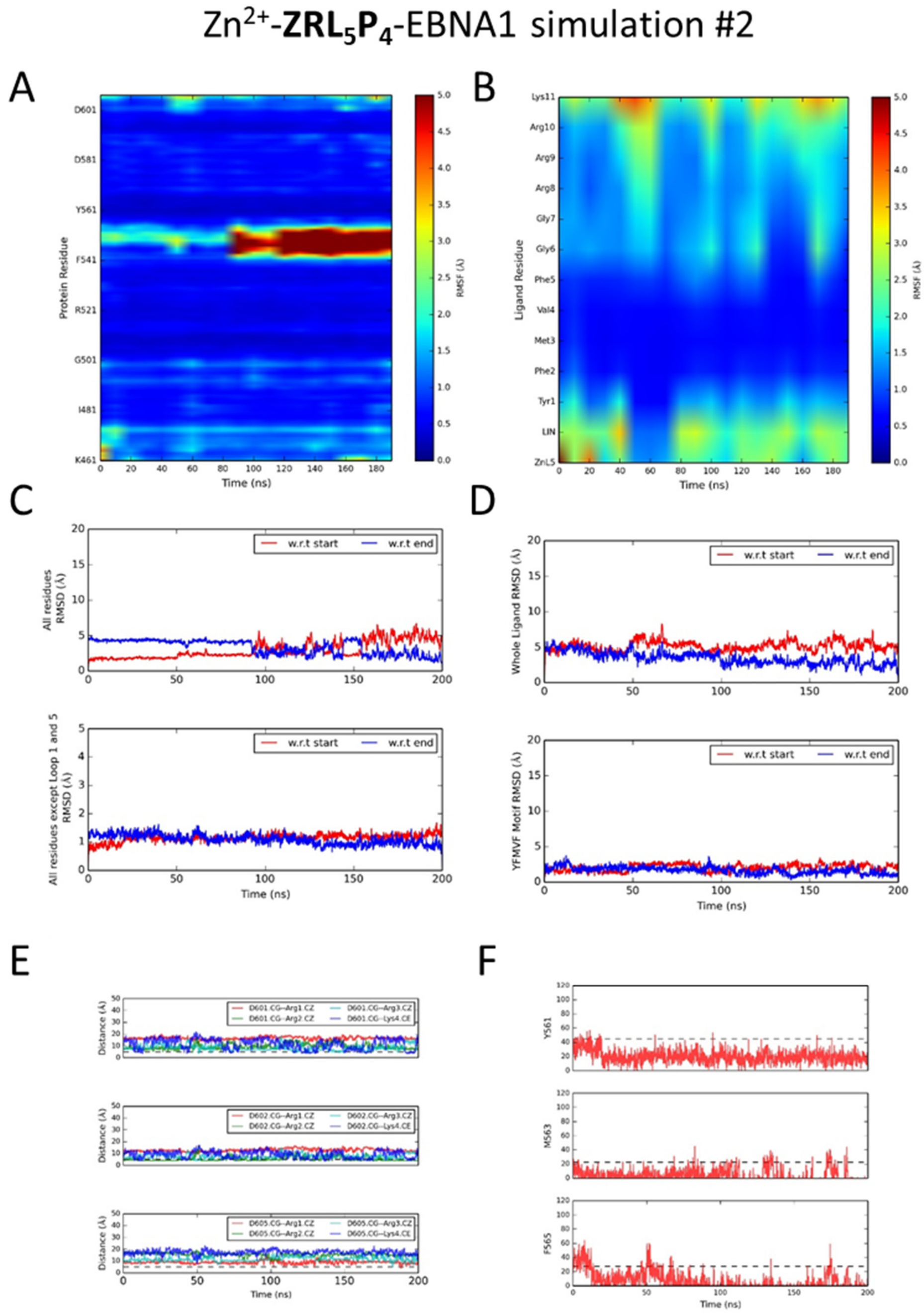

Fig. S9. 200 ns NPT simulation of the Zn^{2+}-**ZRL₅P₄**-EBNA1 complex model (#2). (A) RMSF of the putative EBNA1 DBD monomer. (B) RMSF of Zn^{2+}-**ZRL_5P_4**. (C) RMSD of main-chain atoms of EBNA1 DBD from the initial (red) and end (blue) structures of the complex. (D) RMSD of main-chain atoms of Zn^{2+}-**ZRL_5P_4** from the initial (red) and end (blue) structures of the complex. (E) The salt-bridging between the RrRK motif and the acid residues in the C-terminus of the EBNA1 DBD. (F) The hydrophobic interactions between the YFMVF motif and the dimeric interface of the EBNA1 DBD.

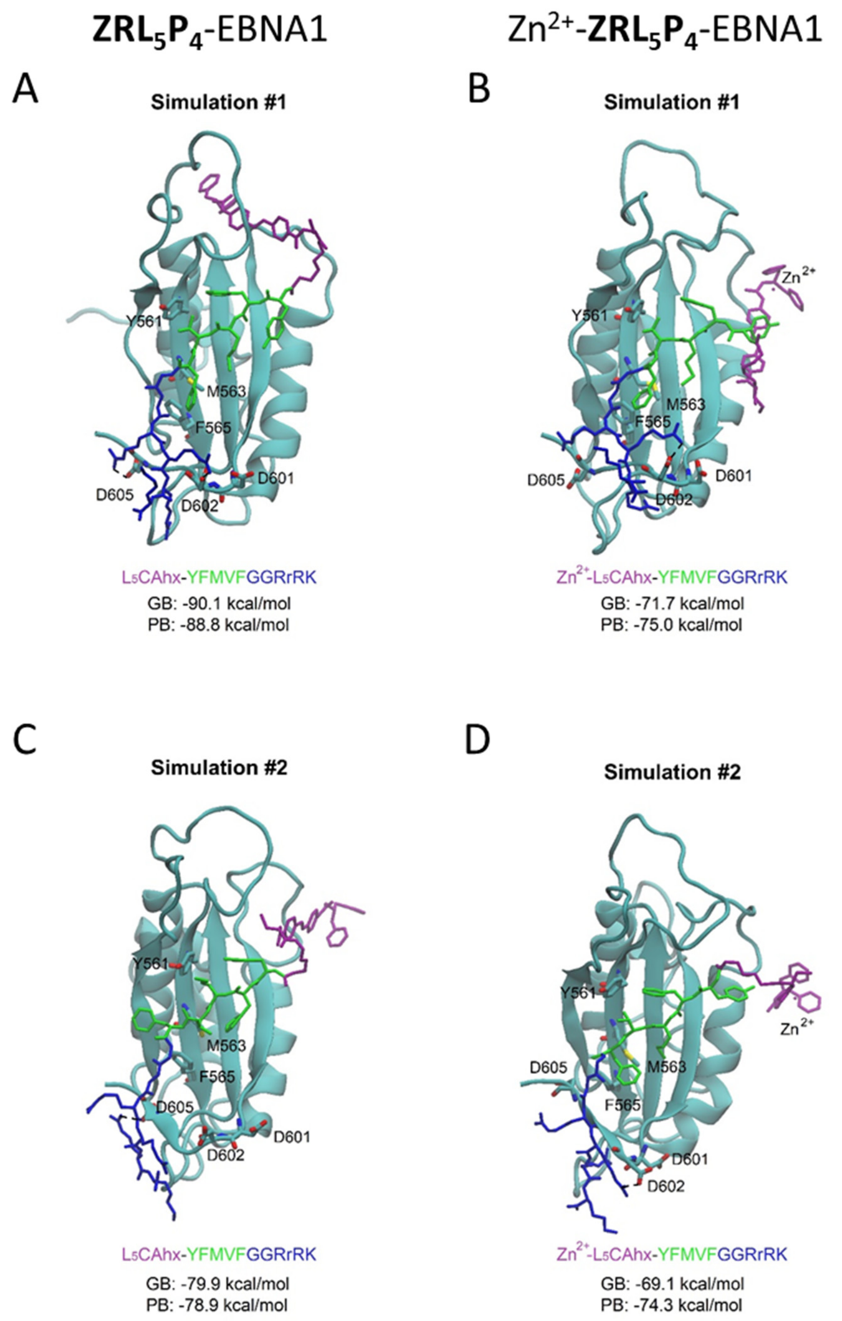

Fig. S10. Putative structures of the **ZRL₅P₄**-EBNA1 and Zn^{2+}-**ZRL₅P₄**-EBNA1 complexes. (A and B) Representative conformation of the **ZRL₅P₄**-EBNA1 complex resulting from two independent MD simulations; the calculated GB and PB values represent their free energy of binding. (C and D) Representative conformation of the Zn^{2+}-**ZRL₅P₄**-EBNA1 complex resulting from two independent MD simulations; the calculated GB and PB values represent their free energy of binding.

Table S1. Modified AMBER force field parameters for Zn^{2+}-free **ZRL₅** residues.

Non-standard residues	Type	Item	Parameters
**ZRL_5**	Bonded	CA-CD	*k*=361.3 kcal/(mol*Å^2), d_o=1.4763 Å
		CA-N	*k*=384.2 kcal/(mol*Å^2), d_o=1.4121 Å
	Angle	CA-C-N	*k*=67.68 kcal/(mol*rad^{-2}), $θ_o$=115.25
		CA-C-O	*k*=68.72 kcal/(mol*rad^{-2}), $θ_o$=122.60
		CA-CA-CD	*k*=64.50 kcal/(mol*rad^{-2}), $θ_o$=120.82
		CA-CA-N	*k*=67.87 kcal/(mol*rad^{-2}), $θ_o$=120.19
		CA-CA-NC	*k*=68.83 kcal/(mol*rad^{-2}), $θ_o$=122.94
		CT-CA-NC	*k*=67.30 kcal/(mol*rad^{-2}), $θ_o$=116.68
		H4-CA-NC	*k*=51.88 kcal/(mol*rad^{-2}), $θ_o$=116.03
		CA-CD-CD	*k*=63.68 kcal/(mol*rad^{-2}), $θ_o$=127.52
		CA-CD-HA	*k*=46.96 kcal/(mol*rad^{-2}), $θ_o$=115.13
		C-CT-NT	*k*=66.32 kcal/(mol*rad^{-2}), $θ_o$=111.14
		CA-CT-H1	*k*=46.99 kcal/(mol*rad^{-2}), $θ_o$=109.56
		CA-CT-NT	*k*=66.22 kcal/(mol*rad^{-2}), $θ_o$=112.16
		C-N-CA	*k*=63.82 kcal/(mol*rad^{-2}), $θ_o$=123.71
		CT-C-N	*k*= 66.79 kcal/(mol*rad^{-2}), $θ_o$=115.18
		N-C-OH	*k*= 74.29 kcal/(mol*rad^{-2}), $θ_o$=112.82
		CA-NC-CA	*k*=68.35 kcal/(mol*rad^{-2}), $θ_o$=117.22
	Dihedral	CA-CA-CD-CD	υ=26.6 kcal/(mol*rad^{-2}), phase=180.0, period=2
		CA-CA-CD-HA	υ=26.6 kcal/(mol*rad^{-2}), phase=180.0, period=2
		CA-N-C-OH	υ=10.0 kcal/(mol*rad^{-2}), phase=180.0, period=2
		CA-N-C-CT	υ=10.0 kcal/(mol*rad^{-2}), phase=180.0, period=2
		N-CA-CA-HA	υ=14.5 kcal/(mol*rad^{-2}), phase=180.0, period=2
		N-CA-CA-CA	υ=14.5 kcal/(mol*rad^{-2}), phase=180.0, period=2
		N-C-OH-HO	υ=4.60 kcal/(mol*rad^{-2}), phase=180.0, period=2
		N-C-CT-H1	υ=0.00 kcal/(mol*rad^{-2}), phase=180.0, period=2
		N-C-CT-NT	υ=0.00 kcal/(mol*rad^{-2}), phase=180.0, period=2
		CA-CA-N-C	υ=1.80 kcal/(mol*rad^{-2}), phase=180.0, period=2

Table S2. Modified AMBER force field parameters for Zn^{2+}-**ZRL$_5$** residues.

Non-standard residues	Type	Item	Parameters
Zn^{2+}-**ZRL$_5$**	Bonded	NE-ZN	k=640.0 kcal/(mol*Å^2), d_o=2.048 Å
		NF-ZN	k=640.0 kcal/(mol*Å^2), d_o=2.201 Å
		NG-ZN	k=640.0 kcal/(mol*Å^2), d_o=2.049 Å
	Angle	CA-C-N	k=70.790 kcal/(mol*rad^{-2}), θ_o=115.08
		CA-NE-ZN	k=50.0 kcal/(mol*rad^{-2}), θ_o=121.90
		CM-NE-ZN	k=50.0 kcal/(mol*rad^{-2}), θ_o=114.0
		NE-ZN-NF	k=50.0 kcal/(mol*rad^{-2}), θ_o=81.2
		NE-ZN-NG	k=50.0 kcal/(mol*rad^{-2}), θ_o=118.0
		CT-NF-ZN	k=50.0 kcal/(mol*rad^{-2}), θ_o=105.0
		NF-ZN-NG	k=50.0 kcal/(mol*rad^{-2}), θ_o=81.30
		CA-NG-ZN	k=50.0 kcal/(mol*rad^{-2}), θ_o=120.0
		NG-ZN-NG	k=50.0 kcal/(mol*rad^{-2}), θ_o=117.40
	Dihedral	X-NE-ZN-X	υ=0.00 kcal/(mol*rad^{-2}), phase=0.0, period=2
		X-ZN-NT-X	υ=0.00 kcal/(mol*rad^{-2}), phase=0.0, period=2
		X-ZN-NC-X	υ=0.00 kcal/(mol*rad^{-2}), phase=0.0, period=2
		X-CA-NE-X	υ=0.00 kcal/(mol*rad^{-2}), phase=0.0, period=2
		X-NE-ZN-X	υ=0.00 kcal/(mol*rad^{-2}), phase=0.0, period=2
		X-NE-CA-X	υ=0.00 kcal/(mol*rad^{-2}), phase=0.0, period=2
		X-CM-NE-X	υ=0.00 kcal/(mol*rad^{-2}), phase=0.0, period=2
		X-NC-ZN-X	υ=0.00 kcal/(mol*rad^{-2}), phase=0.0, period=2
		X-ZN-NF-X	υ=0.00 kcal/(mol*rad^{-2}), phase=0.0, period=2
		X-NF-ZN-X	υ=0.00 kcal/(mol*rad^{-2}), phase=0.0, period=2
		X-ZN-NG-X	υ=0.00 kcal/(mol*rad^{-2}), phase=0.0, period=2
		X-CA-NG-X	υ=0.00 kcal/(mol*rad^{-2}), phase=0.0, period=2
		X-CT-NF-X	υ=0.00 kcal/(mol*rad^{-2}), phase=0.0, period=2
		X-NG-ZN-X	υ=0.00 kcal/(mol*rad^{-2}), phase=0.0, period=2
		X-CT-NF-X	υ=0.00 kcal/(mol*rad^{-2}), phase=0.0, period=2

Table S3. Calculated binding affinities between $\mathbf{ZRL_5P_4}$/Zn^{2+}-$\mathbf{ZRL_5P_4}$ and EBNA1 DBD.

System	Simulation	GB (kcal/mol)	PB (kcal/mol)
$\mathbf{ZRL_5P_4}$-EBNA1 DBD	#1	−90.1365±8.5697	−88.8280±8.7654
	#2	−79.9369±11.5724	−78.9043±13.2484
Zn^{2+}-$\mathbf{ZRL_5P_4}$-EBNA1 DBD	#1	−71.6652±9.1082	−74.9505±10.8770
	#2	−69.1021±12.3023	−74.3209±13.8954

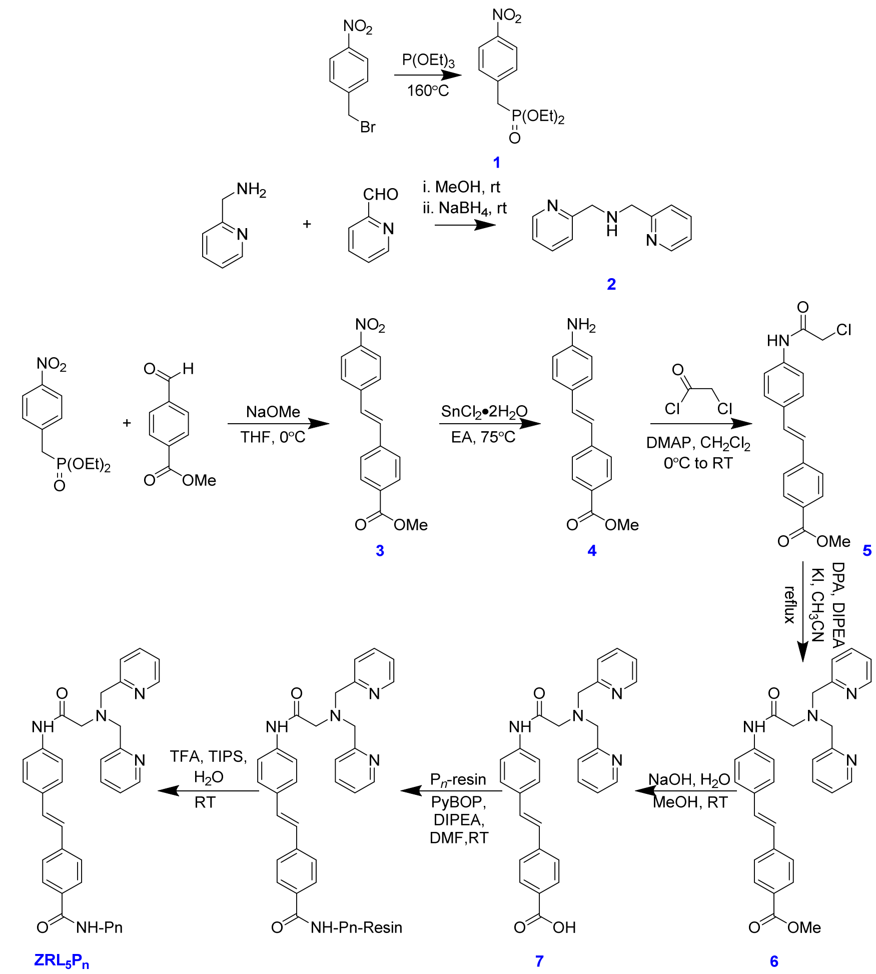

Scheme S1. Synthesis of **ZRL$_5$P$_n$** (n=2:**P$_2$**,YFMVF; n=4:**P$_4$**,C-Ahx-YFMVFGGRrRK; n=6:**P$_6$**,Ahx-YFIVFGGRrRK; Ahx, 6-aminohexanoic acid; r, D-arginine).

Table S4. Gradient of the solvent used for preparative HPLC.

Time (min)	% A	% B	Flow rate (ml/min)
0	80	20	8
35	20	80	8
37	0	100	8
40	80	20	8
41	80	20	8

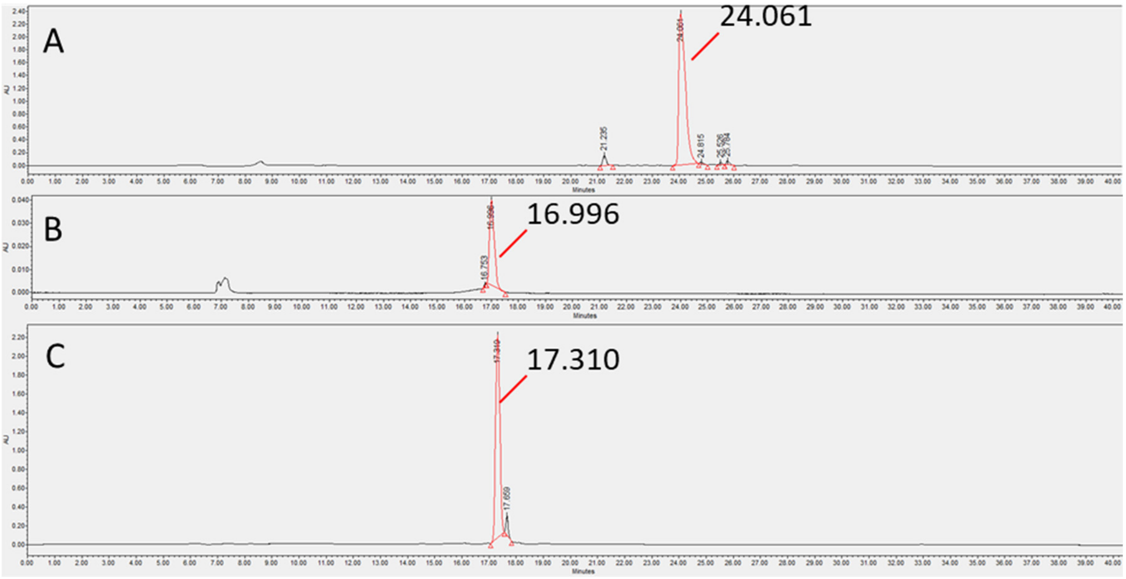

Fig. S11. HPLC spectrum of the purified (A) **ZRL$_5$P$_2$**, (B) **ZRL$_5$P$_4$**, and (C) **ZRL$_5$P$_6$**.

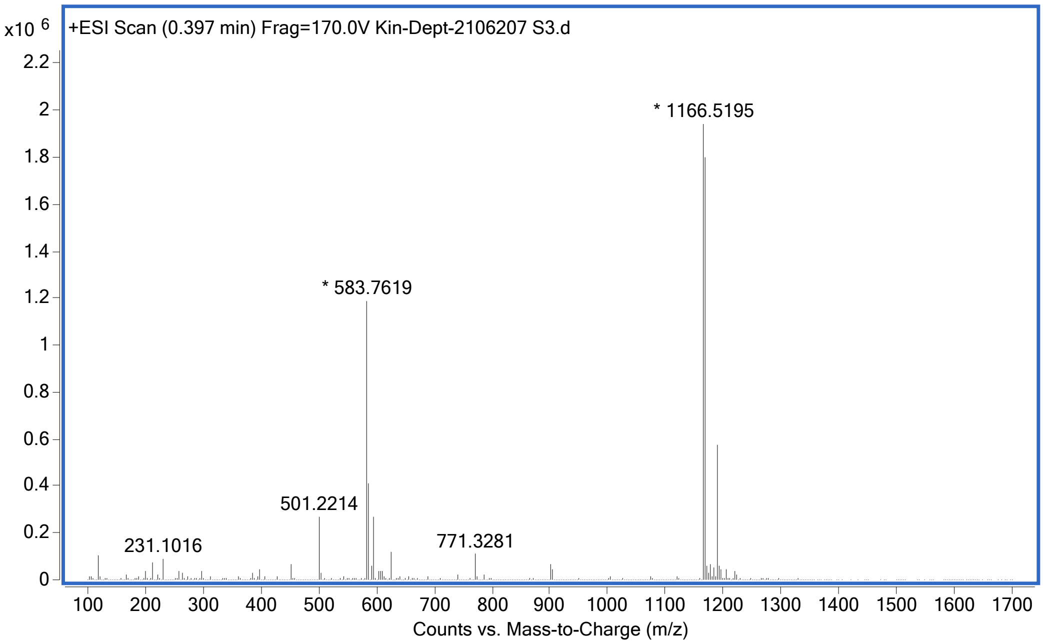
x10 6
+ESI Scan (0.397 min) Frag=170.0V Kin-Dept-2106207 S3.d
* 1166.5195
* 583.7619
501.2214
771.3281
231.1016
Counts vs. Mass-to-Charge (m/z)

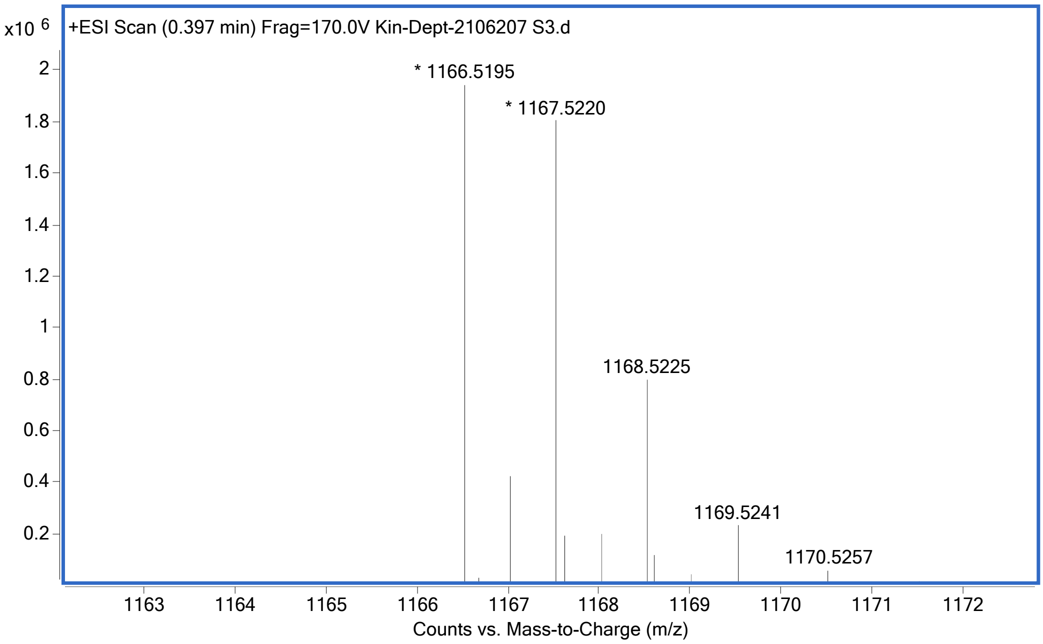
x10 6
+ESI Scan (0.397 min) Frag=170.0V Kin-Dept-2106207 S3.d
* 1166.5195
* 1167.5220
1168.5225
1169.5241
1170.5257
Counts vs. Mass-to-Charge (m/z)

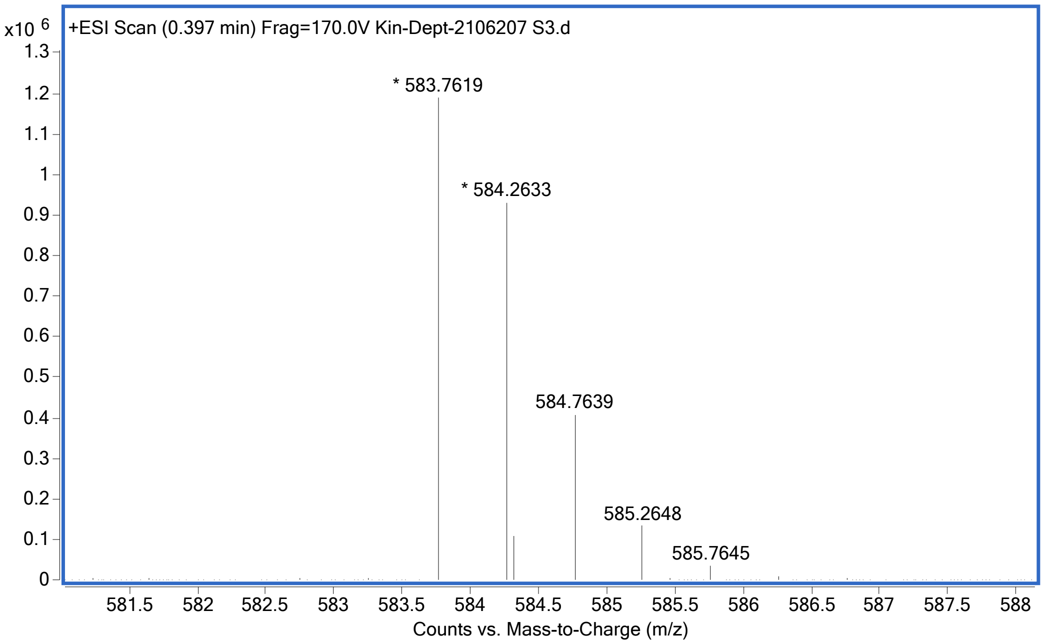

Fig. S12. High-resolution mass spectrum of **ZRL_5P_2**.

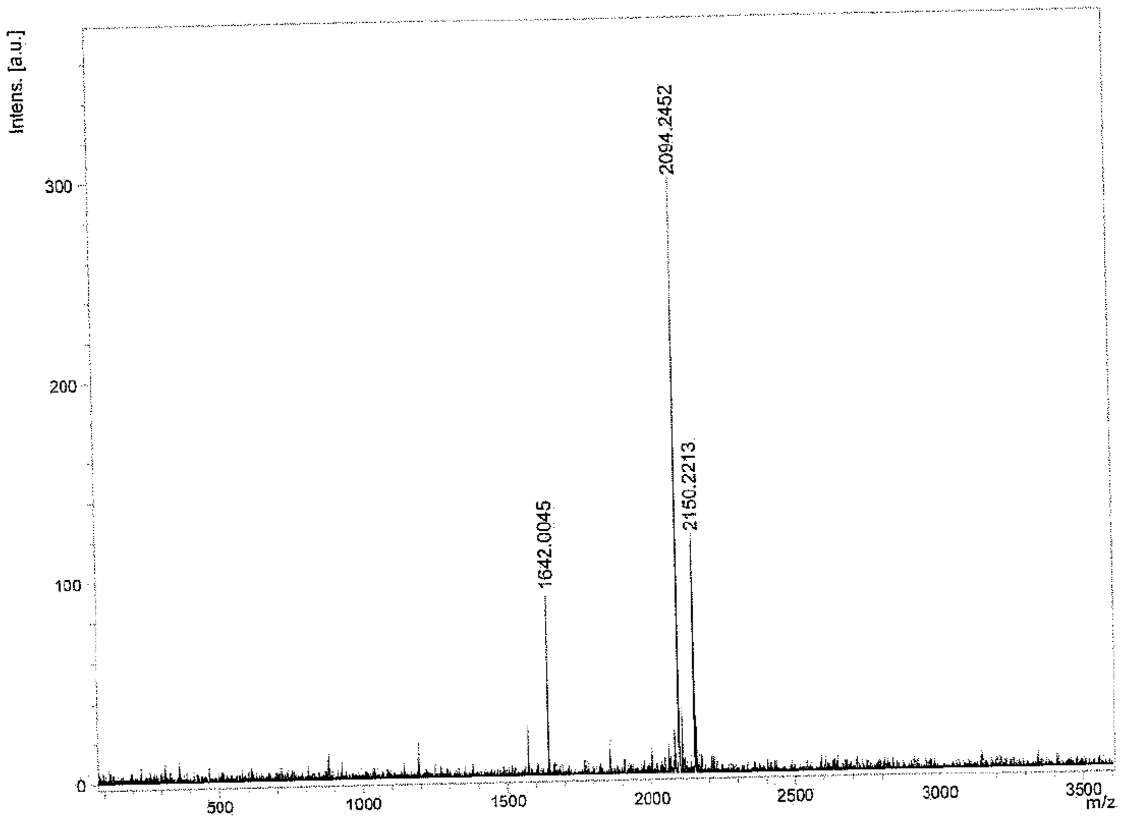

Fig. S13. High-resolution mass spectrum of **ZRL$_5$P$_4$**.

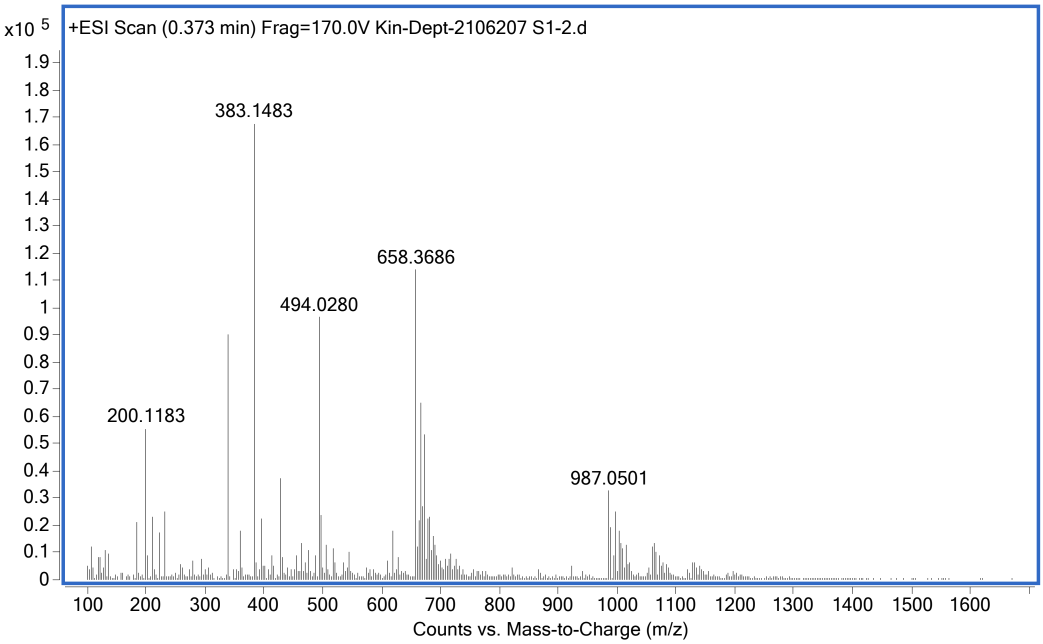
x10 5
+ESI Scan (0.373 min) Frag=170.0V Kin-Dept-2106207 S1-2.d
383.1483
658.3686
494.0280
200.1183
987.0501
Counts vs. Mass-to-Charge (m/z)

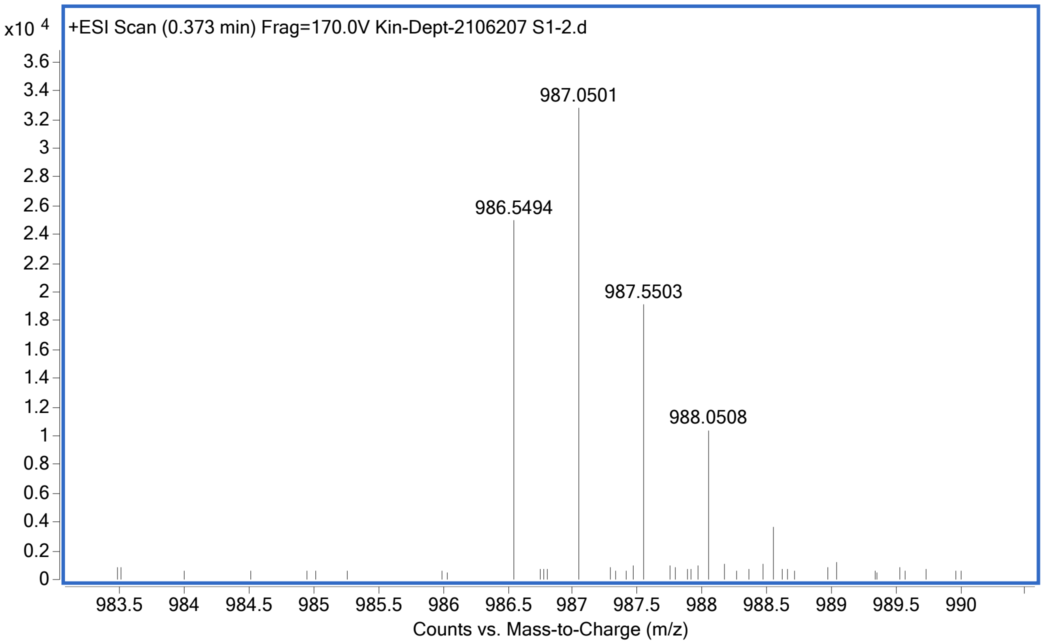
x10 4
+ESI Scan (0.373 min) Frag=170.0V Kin-Dept-2106207 S1-2.d
987.0501
986.5494
987.5503
988.0508
Counts vs. Mass-to-Charge (m/z)

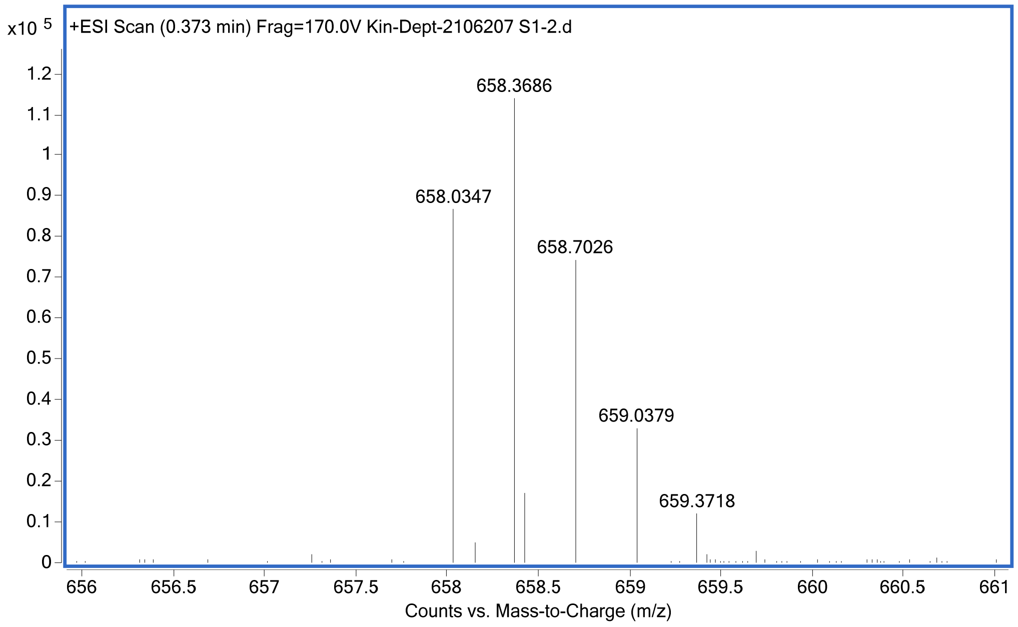

Fig. S14. High-resolution mass spectrum of **ZRL_5P_6**.

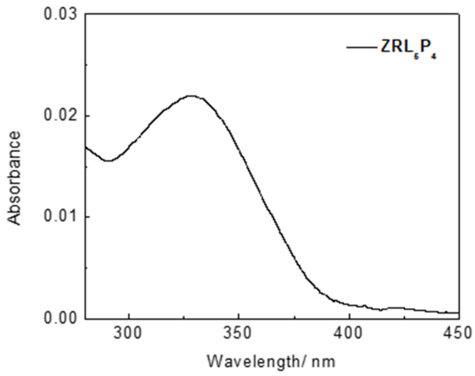

Fig. S15. Absorption spectrum of **ZRL$_5$P$_4$** (2 μM) in HEPES buffer.

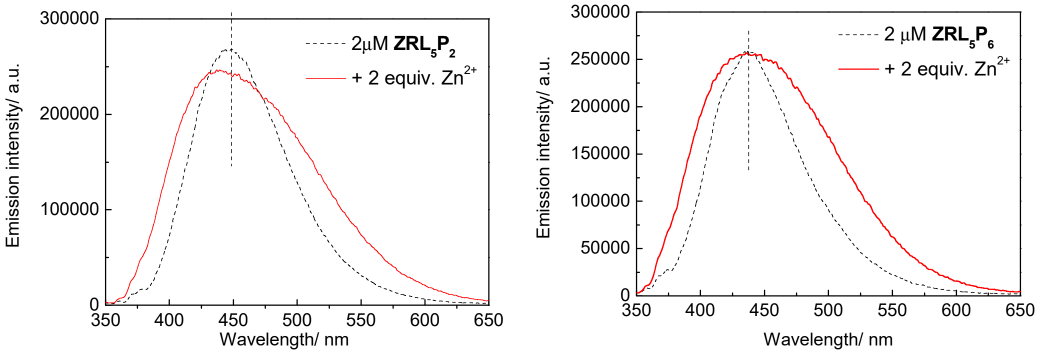

Fig. S16. Fluorescence spectral changes of **ZRL$_5$P$_2$** (left) and **ZRL$_5$P$_6$** (right) (2 μM; excitation 337 nm) in the presence of Zn^{2+} in aqueous solution [CH_3CN/0.05 M HEPES (pH 7.4), 50:50].

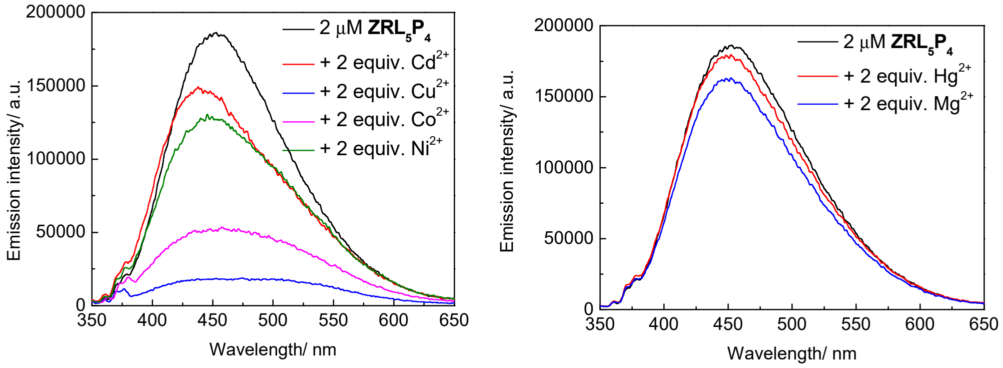

Fig. S17. Fluorescence spectral changes of **ZRL_5P_4** (2 μM; excitation 337 nm) in the presence of Cd^{2+}, Cu^{2+}, Co^{2+} and Ni^{2+} (left), Hg^{2+} and Mg^{2+} (right) in aqueous solution [CH_3CN/0.05 M HEPES (pH 7.4), 50:50].

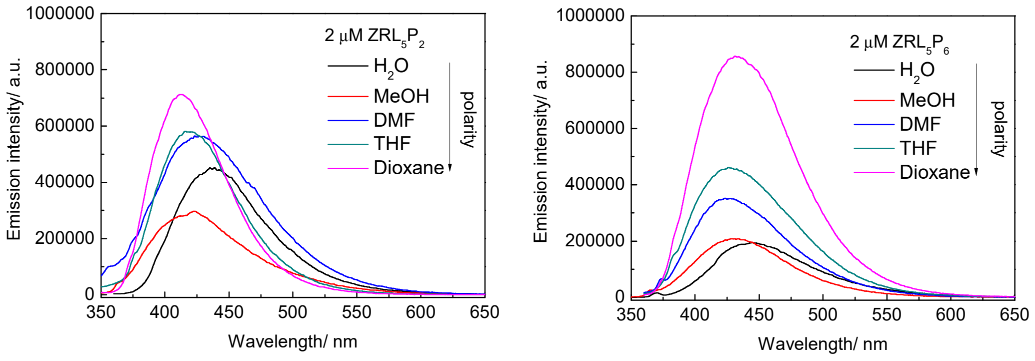

Fig. S18. Solvation study of **ZRL₅P₂** (left) and **ZRL₅P₆** (right) with a decrease of the polarity of the solvent.

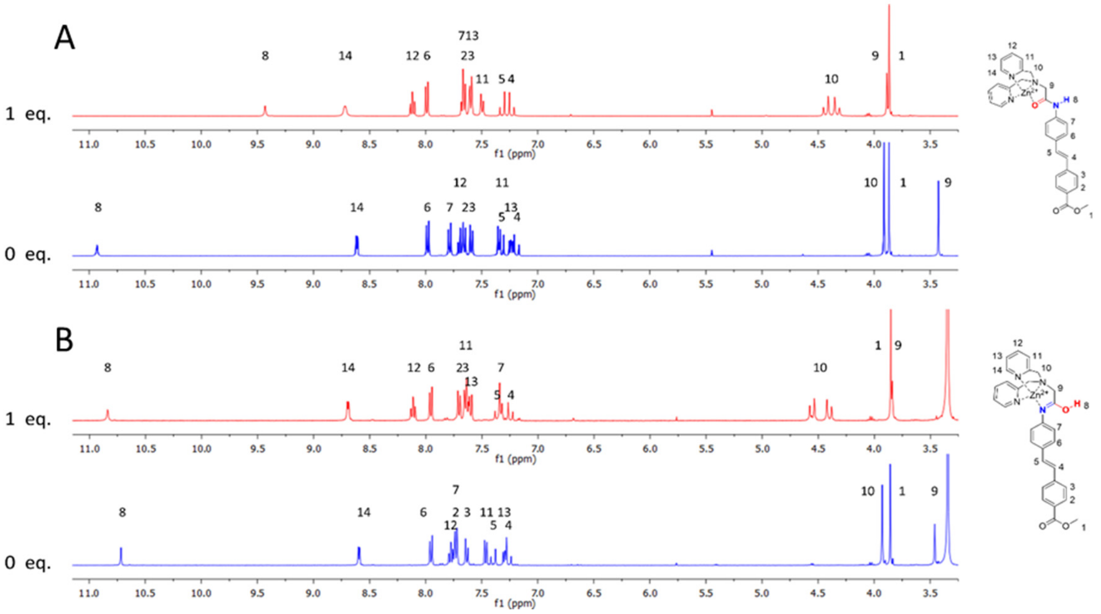

Fig. S19. ^{1}H NMR spectra of compound **5** in the presence of Zn^{2+} in (A) CD_3CN and (B) DMSO-d_6. Binding of the carbonyl oxygen to Zn^{2+} in CD_3CN blocks the resonance structure of the amide group and shifts proton 8 upfield. Binding of the amide nitrogen to Zn^{2+} in DMSO-d_6 functions as the electron-withdrawing group to shift proton 8 downfield.

Table S5. STR profiles of cell lines.

Cell line / Locus	C666-1	HK-1	HONE-1	HONE-1-EBV	NPC43	Raji
**D3S1358**	17	15	15.3, 16.3, 18.2	15..3, 16.3, 18.2	18	15, 16
**vWA**	17, 20	18,19	14, 16	14, 16	14, 16	17, 19
**D7S820**	11, 12	8, 11	10, 12	10, 12	12, 13	10
**CSF1PO**	11, 12	11, 12	10, 11	10, 11	12, 13	10, 12
**Penta E**	11, 15	15, 19	17	17	9.2, 12.2	5, 13
**D8S1179**	11, 13, 15	11, 13	13	13	11	14, 15
**D21S11**	29, 31.2	28, 29	27, 30	27, 30	32	28, 31
**D16S539**	10	9, 11	9, 10, 11	9, 10, 11	9, 10	8, 11
**D2S1338**	16, 23	20, 23	17, 23	17, 23	17, 21	22
**Penta D**	9, 10	9, 12	9, 12	9, 12	8	3.2, 9
**D19S433**	13, 14.2, 15.2	14, 16	13.2	13.2	13, 14, 15.2	14, 14.2
**TH01**	5.3, 7.3	7, 9	6, 7, 9	6, 7, 9	7	6, 7
**D13S317**	8, 11	11	10, 12, 14	10, 12, 14	12	13
**TPOX**	8, 11	8, 11	8, 12	8, 12	8	8, 13
**D18S51**	16	17	13, 16	13, 16	13, 15, 16.2	17
**D6S1043**	19	12, 20	11, 14, 18	11, 14, 18	13, 18	21
**AMEL**	X, Y	X	X	X	X	X, Y
**D1S1656**	12, 17.3	15	13, 15.2	13, 15.2	16	14
**D5S818**	10, 11	11, 13	11, 12	11, 12	11	10, 13
**D12S391**	23	17, 19	20, 21	20, 21	16, 19	18, 19
**FGA**	23, 24	26	17.2, 20.2	17.2, 20.2	22	18.3, 27
**Matched to**	C6661/ CVCL_7949	NPC/HK1/ CVCL_7084	HONE1/ CVCL_8706	HONE1/ CVCL_8706	Nat. Commu, 2018, 9, 4663	Raji/ CVCL_0511

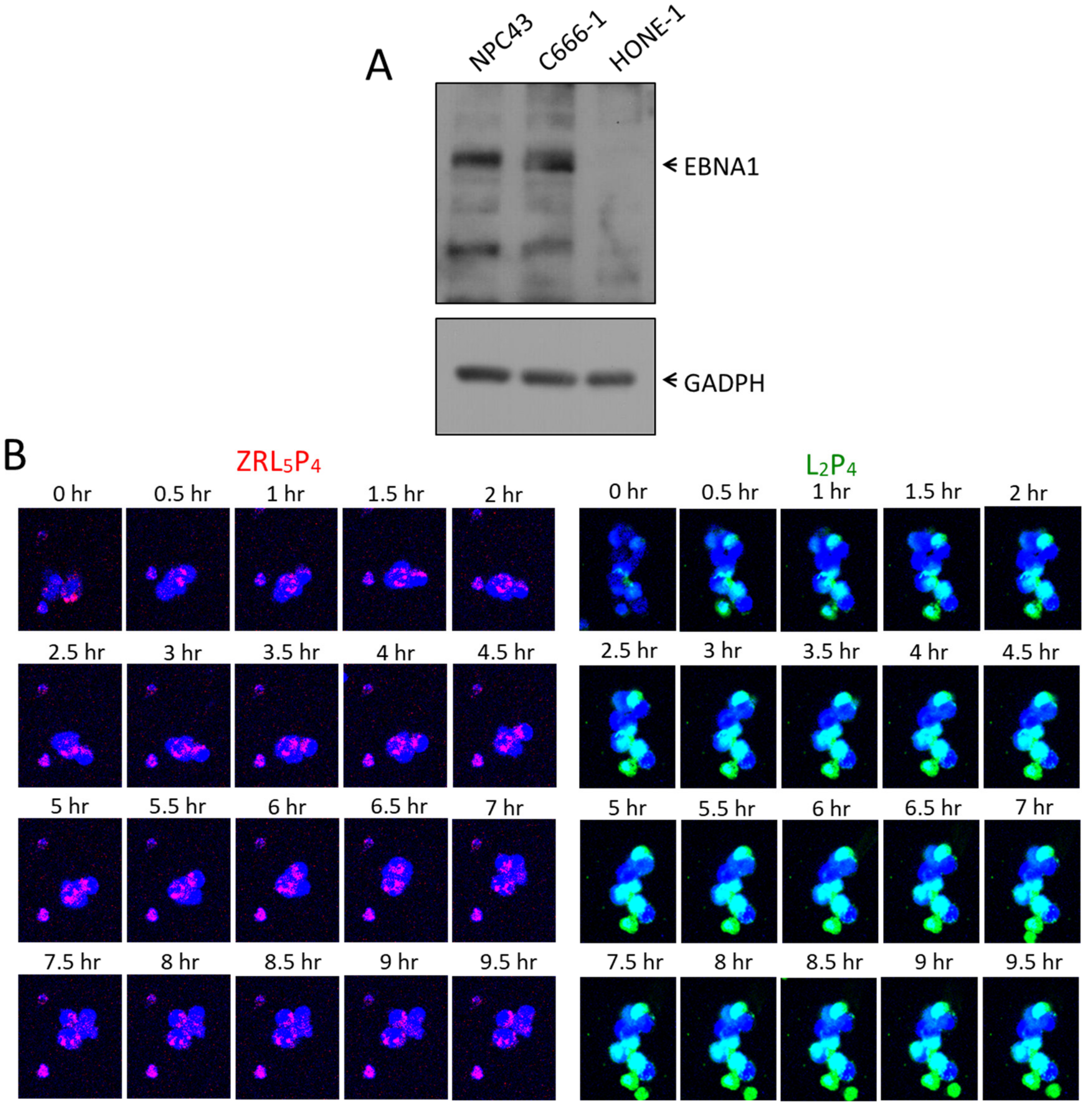

Fig. S20. (A) Protein expression analysis of EBNA1 in NPC43, C666-1, and HONE1 cells. The protein expression was detected with Western blot, and GAPDH serves as the loading control. (B) Comparison of cellular uptake of **ZRL$_5$P$_4$** versus **L$_2$P$_4$** in C666-1 cells. Images were captured by confocal microscopy, and time lapse analysis was performed from 0.5 to 9.5 hours after the addition of **ZRL$_5$P$_4$** or **L$_2$P$_4$**. The red fluorescence indicates signals emitted from **ZRL$_5$P$_4$**, whereas the green fluorescence indicates signals emitted from **L$_2$P$_4$**.The white arrows indicate the presence of **L$_2$P$_4$** signal in both the cytoplasm and the nucleus of a C666-1 cell. The nuclei were stained with Hoechst 33342 dye, and indicated by the blue fluorescent signal. Magnification, 200 ×. Scale bar, 5 µm.

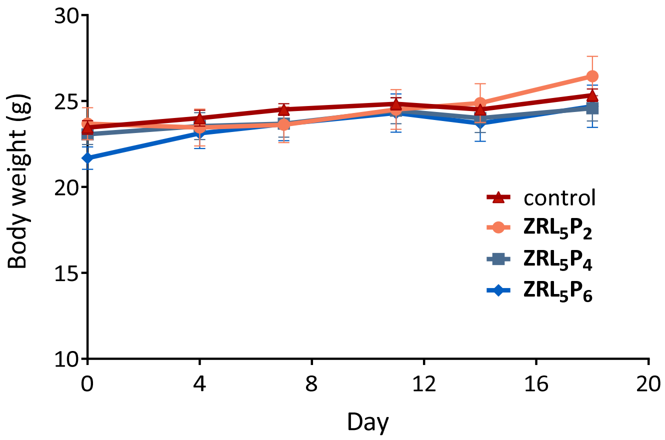

Fig. S21. Effect of $\mathbf{ZRL_5P_2}$, $\mathbf{ZRL_5P_4}$, and $\mathbf{ZRL_5P_6}$ on body weights. Data are expressed as the mean ±SEM. *P<0.05; **P<0.005; ***P<0.005 vs. control. Treatment with $\mathbf{ZRL_5P_2}$, $\mathbf{ZRL_5P_4}$, and $\mathbf{ZRL_5P_6}$ did not cause any significant changes in body weights when compared to the control.

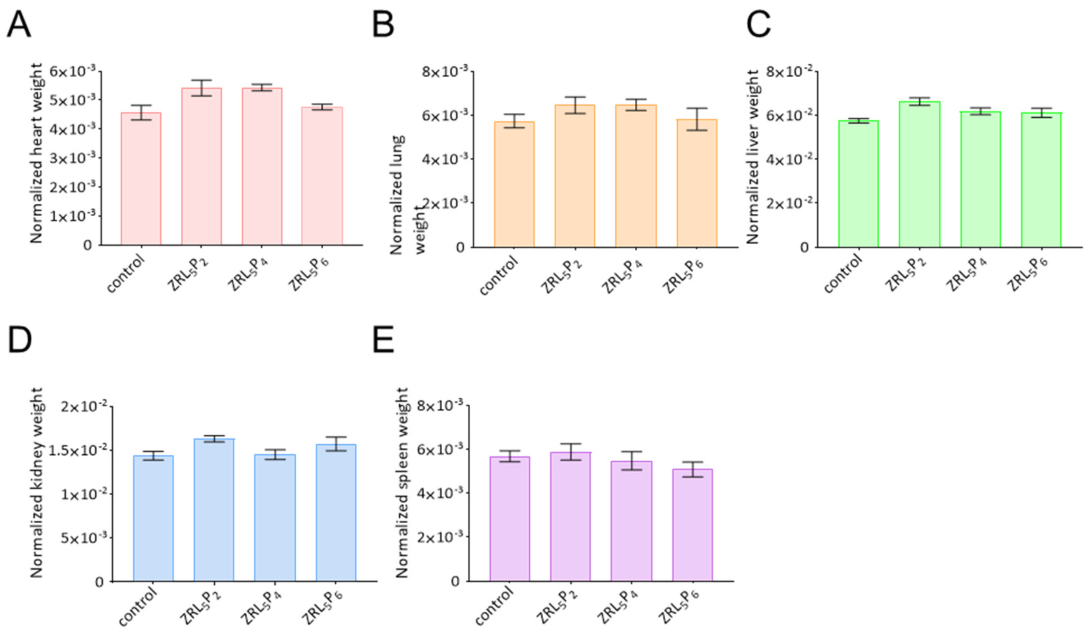

Fig. S22. Effect of **ZRL$_5$P$_2$**, **ZRL$_5$P$_4$**, and **ZRL$_5$P$_6$** on weights of vital organs. Weights of (A) heart, (B) lung, (C) liver, (D) kidney and (E) spleen normalized to mouse body weights at experimental endpoint. Data are expressed as mean ± SEM. *P<0.05; **P<0.005; ***P<0.005 vs. control. Treatment with **ZRL$_5$P$_2$**, **ZRL$_5$P$_4$**, and **ZRL$_5$P$_6$** did not cause any significant changes in organ weights when compared to the control.

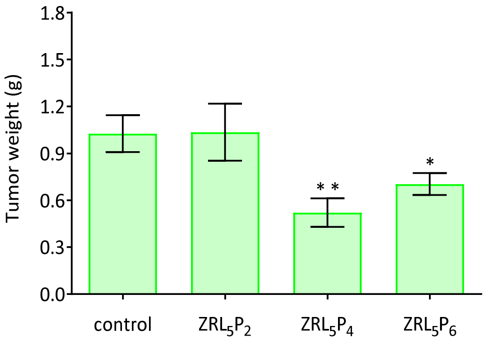

Fig. S23. *In vivo* anti-tumour activity. Mice were treated twice weekly with 4 µg/ injection of the probes for 18 days. At the experimental endpoint, tumours were excised and weighted. Data are expressed as the mean ±SEM. *P<0.05; **P<0.005 vs. control.

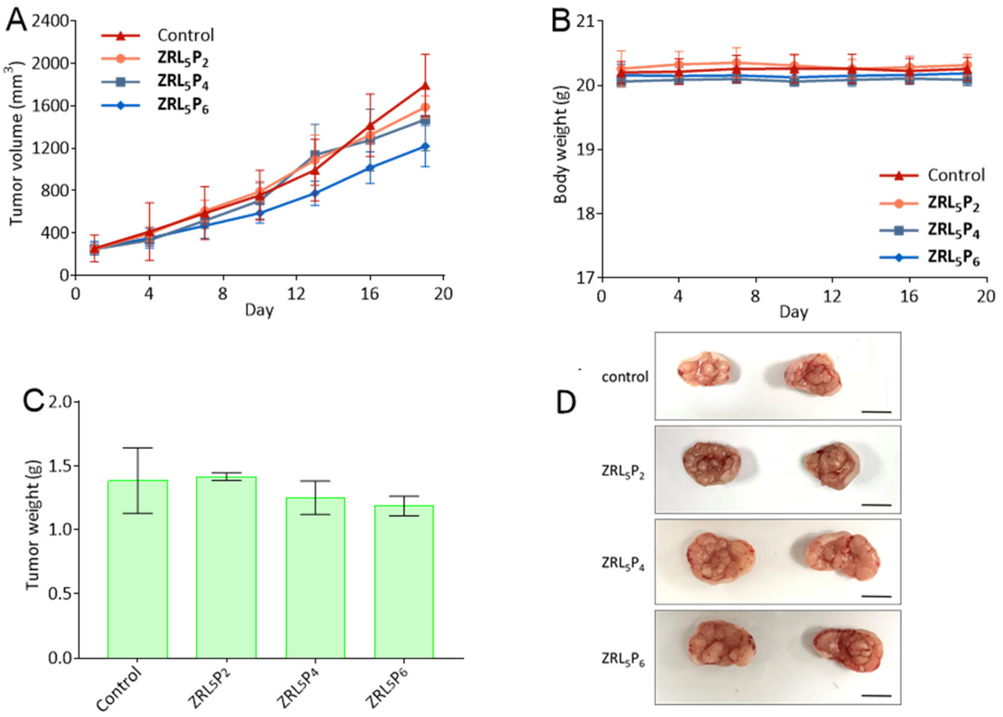

Fig. S24. *In vivo* anti-tumour activity of **ZRL₅P₂**, **ZRL₅P₄**, and **ZRL₅P₆** in EBV-negative HeLa xenografts. Mice were treated every three days with 4 µg/ injection of the probes for 19 days. Throughput the treatment period, (A) tumour volumes and (B) body weights were measured. At the experimental endpoint, tumours were excised. (C) tumour weights and (D) representative photographs of tumours. Scale bar: 10 mm. Data are expressed as mean ± SEM. *P < 0.05; **P < 0.005; ***P < 0.005 vs. control. There was no significant difference in both the tumour volume and tumour weight between the control mice and those treated with the probes. Also, treatment with **ZRL₅P₂**, **ZRL₅P₄**, and **ZRL₅P₆** did not cause any significant changes in body weights compared to the control.

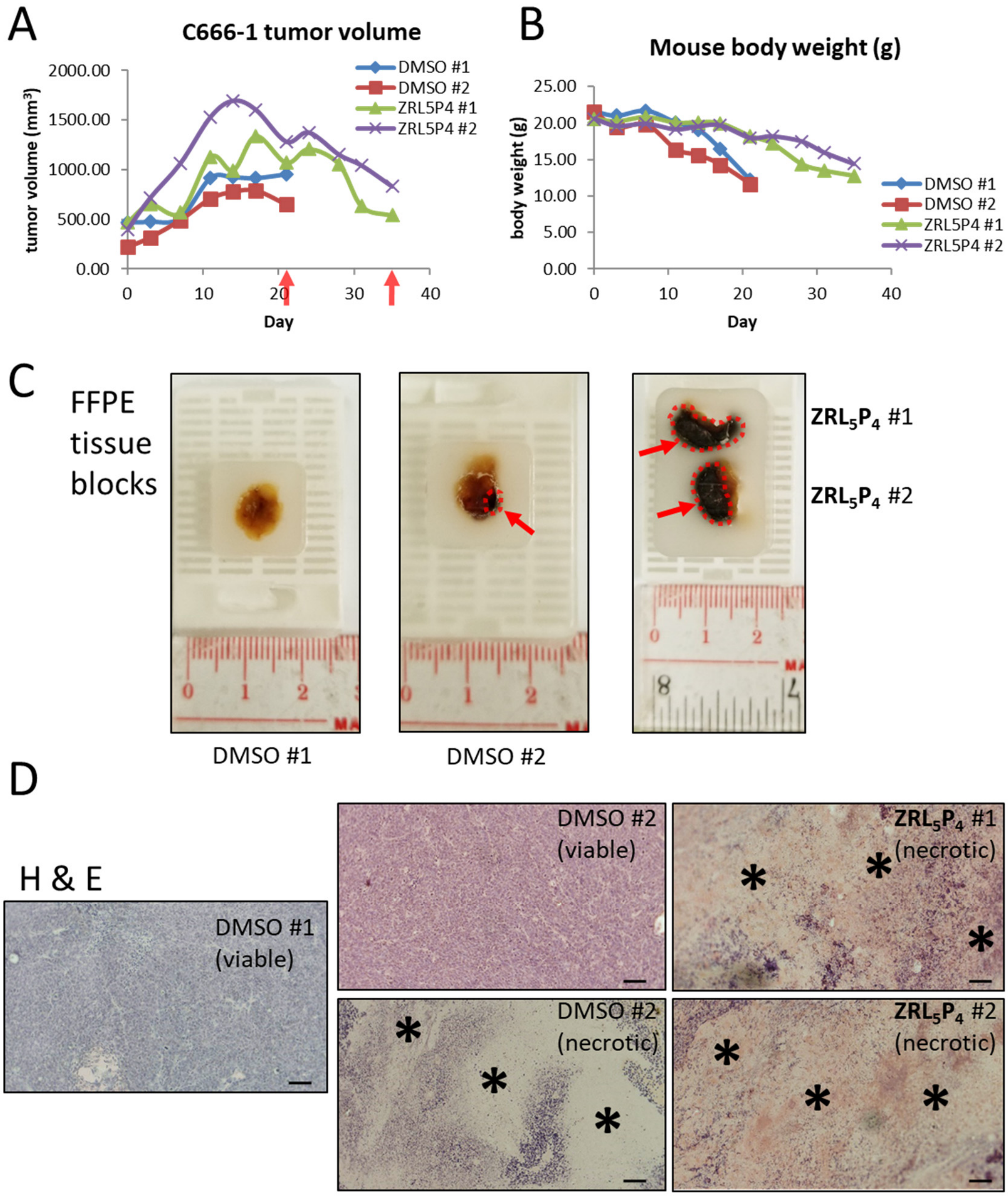

Fig. S25. Analysis of mice and their transplanted tumours with plasma EBV DNA examined. *In vivo* anti-tumour activity of **ZRL₅P₄**. Mice transplanted with C666-1 – derived tumours were treated twice weekly with 4 µg/injection of **ZRL₅P₄** for 35 days. Throughout the treatment period, (A) tumour volumes and (B) body weights were measured. At the experimental endpoint indicted by the red arrows, tumours were excised. (C) Cross-section images of tumour tissue blocks derived from the animal for plasma EBV DNA analysis. The red arrows and dotted lines indicate the areas necrotic tissues. (D) Representative hematoxylin and eosin (H & E) staining images of tumour sections derived from the above *in vivo* animal study. Cell necrosis (acellular areas indicated by *) was observed in the tumour nodules. Magnification, 40 ×. Scale bar, 20 µm.

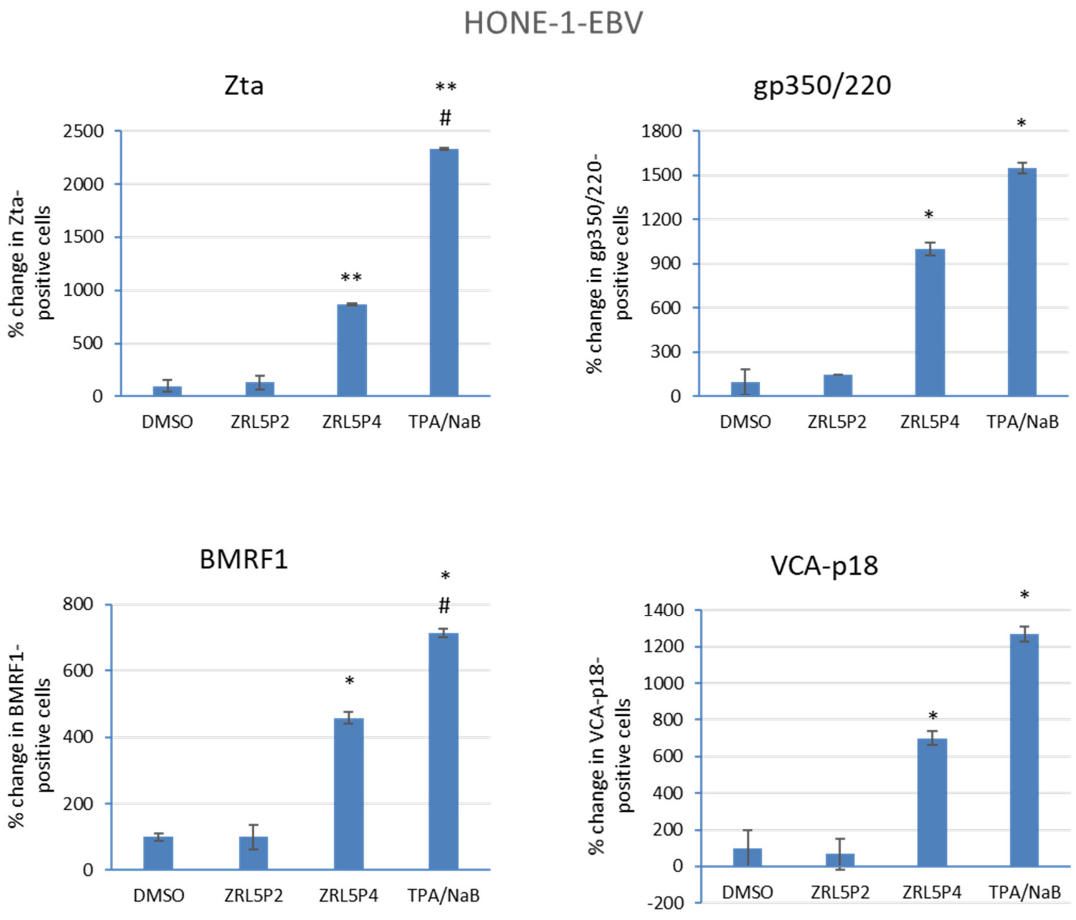

Fig. S26. Quantitative analysis of the immunofluorescent staining results of lytic proteins shown in Fig. 5B. The number of Zta, BMRF1, gp350/220, and VCA-p18 positive HONE-1-EBV cell were counted and the percentage changes in response to 10 µM **ZRL$_5$P$_2$**, **ZRL$_5$P$_4$**, and TPA/NaB when compared to the solvent control (DMSO) are shown in the bar charts. **P < 0.01, *P < 0.05, statistically significant difference from the solvent control (DMSO); #P < 0.05 from the treatment with **ZRL$_5$P$_4$**.

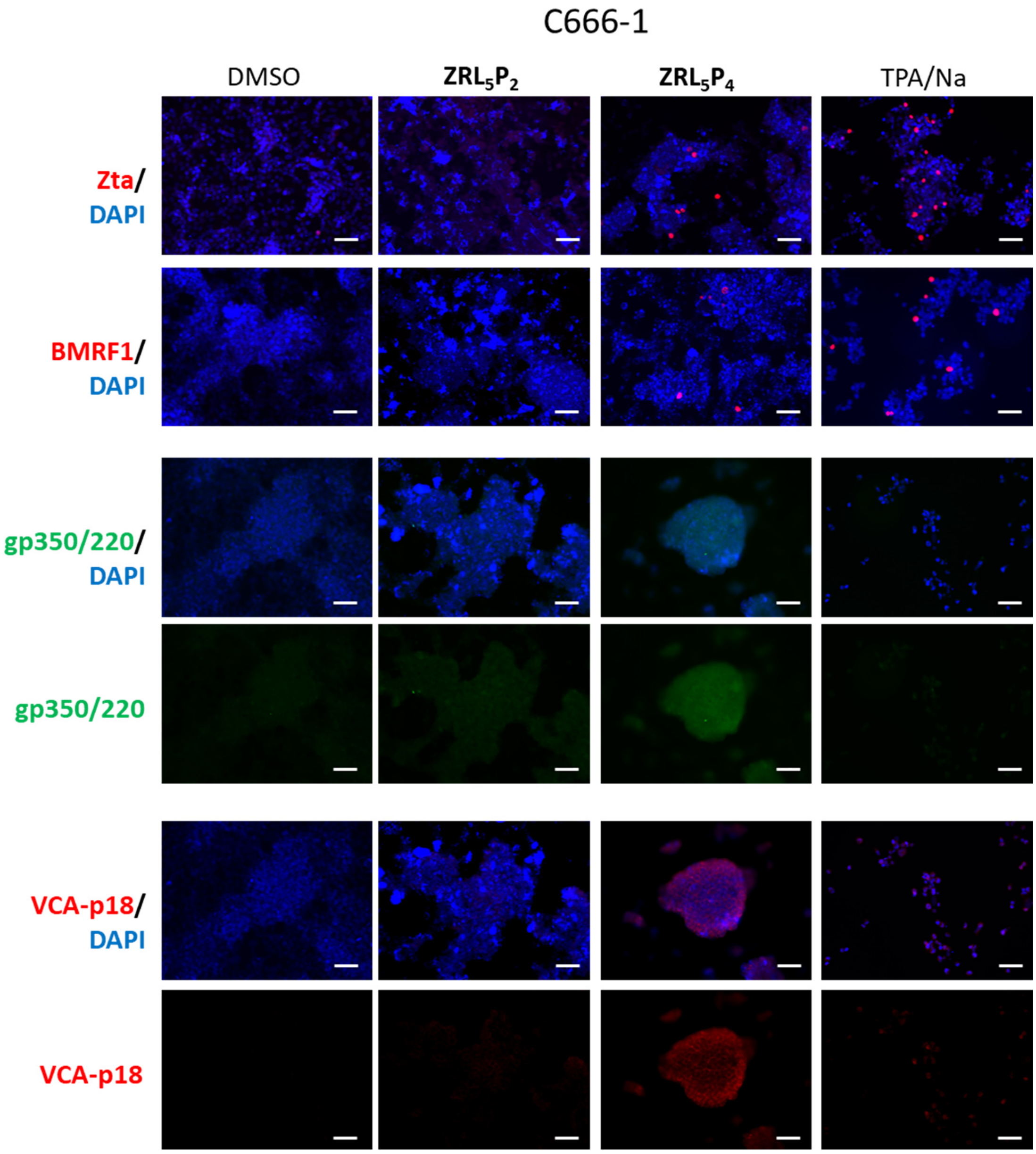

Fig. S27. Representative images of immunofluorescent analysis of EBV lytic proteins, Zta, BMRF1, gp350/220, VCA-p18 in C666-1 cells in response to **ZRL$_5$P$_2$** (10 µM), **ZRL$_5$P$_4$** (10 µM), and TPA/NaB (20 ng/mL TPA, 3 mM NaB). The gp350/220 and VCA-p18 were co-stained in the same slides. Red signals indicate the presence of Zta, BMRF1, and VCA-p18, whereas the green signals indicate the presence of gp350/220. The nuclei were counter-stained with DAPI and indicated in blue. For clearer comparison of gp350/220 and VCA-p18 in response to each treatment, images of the target protein signal with DAPI and the target signal-alone are shown in parallel. Scale bar, 50 µm.

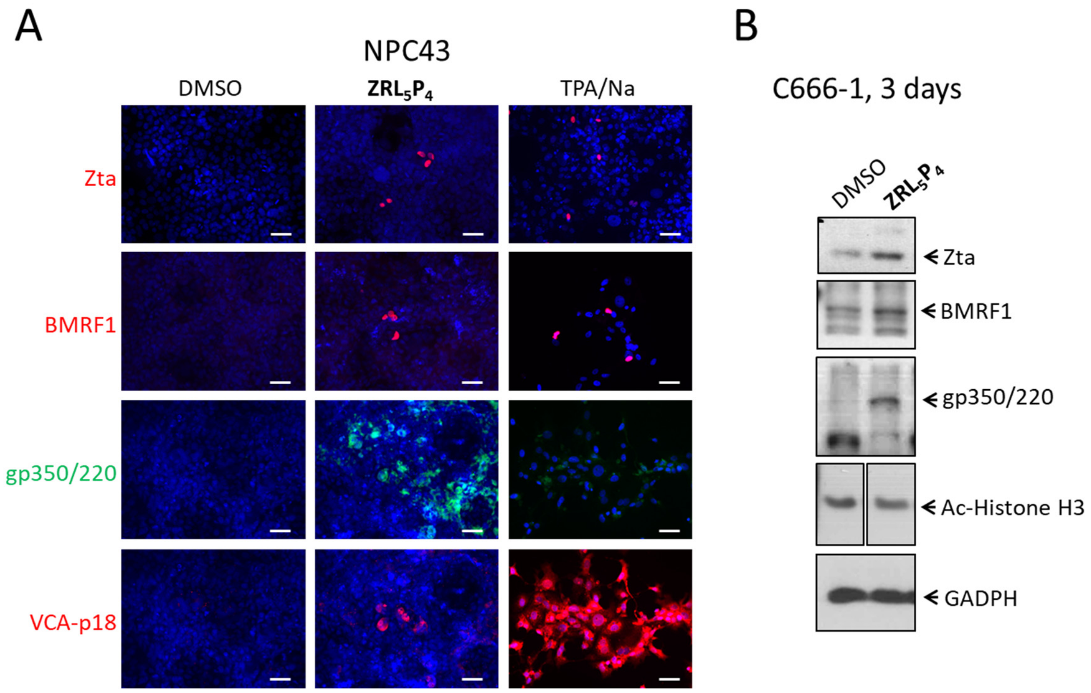

Fig. S28. (A) Representative images of immunofluorescent analysis of EBV lytic proteins, Zta, BMRF1, gp350/220, VCA-p18 in NPC43 cells in response to **ZRL_5P_4** (10 μM) and TPA/NaB (20 ng/mL TPA, 3 mM NaB). The gp350/220 and VCA-p18 were co-stained in the same slides. Red signals indicate the presence of Zta, BMRF1, and VCA-p18, whereas the green signals indicate the presence of gp350/220. The nuclei were counter-stained with DAPI and indicated in blue. Scale bar, 50 μm. (B) Western blot analysis of Zta, BMRF1, gp350/220, and Acetylated histone H3 (Ac-Histone H3) in C666-1 cells cultured with or without 10 μM **ZRL_5P_4** for 3 days. GADPH serves as the loading control.

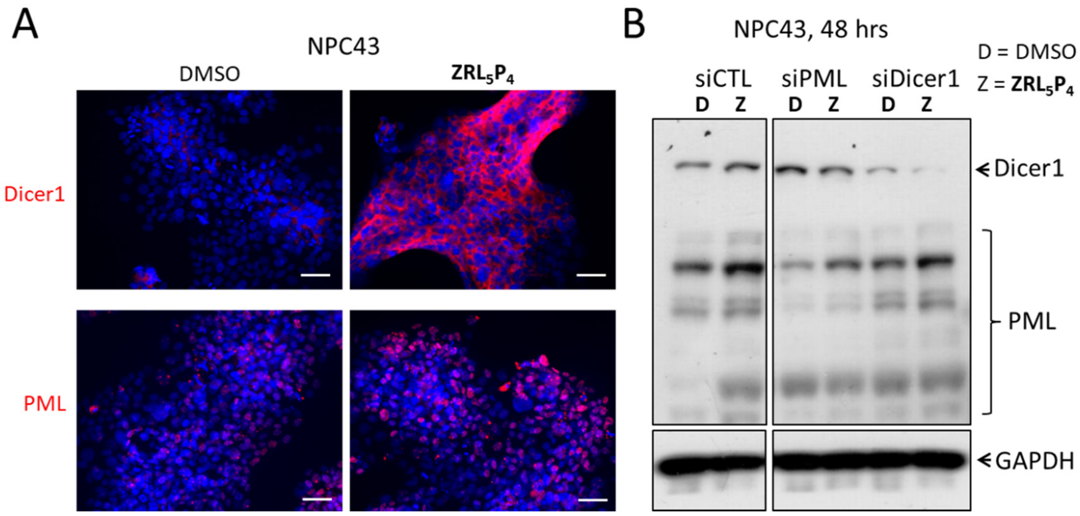

Fig. S29. (A) Representative images of immunofluorescent analysis of Dicer1 and PML in NPC43 in response to **ZRL$_5$P$_4$** (10 μM). Red signals indicate the presence of Dicer1 and PML. The nuclei were counter-stained with DAPI and indicated in blue. Scale bar, 50 μm. (B) Protein expression analysis of NPC43 cells with Dicer1 or PML gene silencing in response to **ZRL$_5$P$_4$** (10 μM for 2 days). Gene silencing was achieved with the respective siRNAs. The negative control siRNA (siCTL) was included. These proteins were detected with Western blot. GADPH serves as the loading control. D, DMSO; Z, **ZRL$_5$P$_4$**.

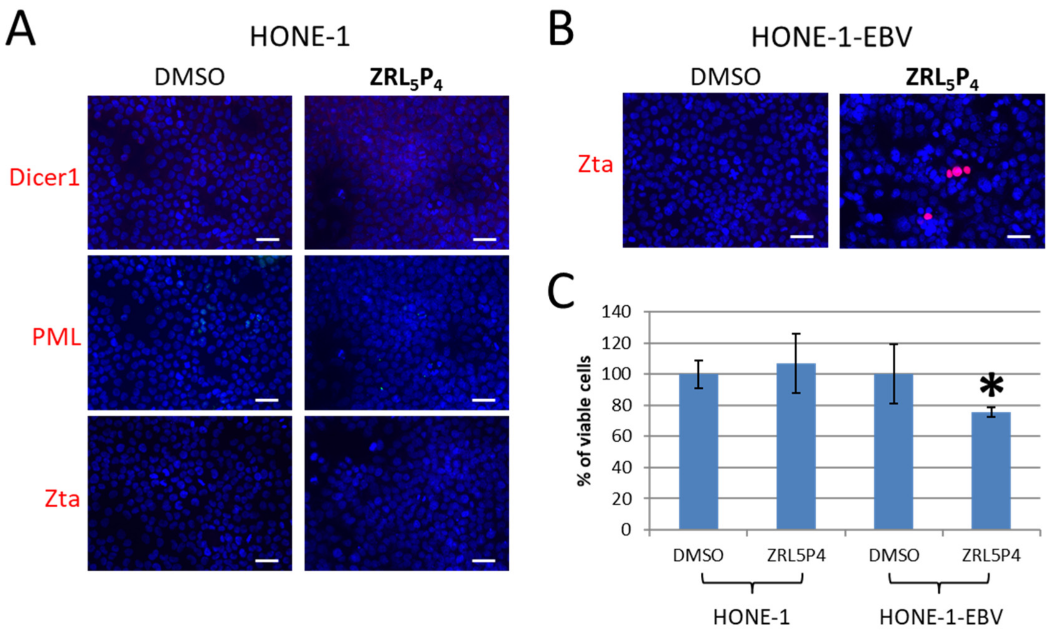

Fig. S30. (A) Representative images of immunofluorescent analysis of Dicer1, PML, and Zta in the EBV-negative HONE-1 cells in response to **ZRL_5P_4** (10 μM). The simultaneous detection of Zta in HONE-1 and Scale bar, 50 μm. (B) HONE-1-EBV to demonstrate the lytic-inducing activities of **ZRL_5P_4**. Red signals indicate the presence of Dicer1, PML, and Zta. The nuclei were counter-stained with DAPI and indicated in blue. Scale bar, 50 μm. (C) Cell viability of HONE1 and HONE-1-EBV in response to **ZRL_5P_4** (10 μM) for 3 days. MTT assay was used to detect the cell viability. The percentage change of cell viability was compared with the solvent control (DMSO). $*P < 0.05$, statistically significant difference from the solvent control (DMSO).

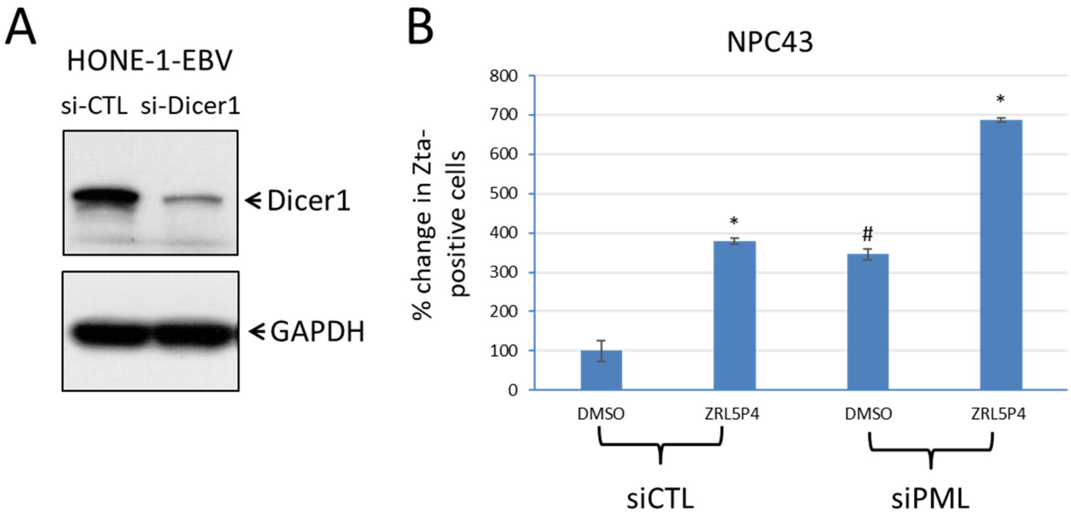

Fig. S31. (A) Protein expression analysis of Dicer1 in HONE-1-EBV cells after Dicer1 gene silencing. These proteins were detected with Western blot. GAPDH serves as the loading control. D, DMSO; Z, **ZRL_5P_4**. (B) Comparison of the number of Zta-positive cells after treatment with **ZRL_5P_4** in the presence versus the absence of PML. NPC43 were included. Gene silencing of PML was achieved by siRNA transfection. Zta was detected by immunofluorescent analysis. Control siRNA (siCTL) was used as a negative control. Relative percentage of Zta-positive was compared against the DMSO solvent control in the siCTL cells. *$P < 0.05$, statistically significant difference from the solvent control (DMSO) in the siCTL cells.

References

1. L. Jiang et al., EBNA1-targeted probe for the imaging and growth inhibition of tumours associated with the Epstein–Barr virus. Nat. Biomed. Eng. 1, 0042 (2017).
2. M. Kurcinski, M. Jamroz, M. Blaszczyk, A. Kolinski, S. Kmiecik, CABS-dock web server for the flexible docking of peptides to proteins without prior knowledge of the binding site. Nucleic Acids Res. 43, W419-424 (2015).
3. A. W. Gotz et al., Routine Microsecond Molecular Dynamics Simulations with AMBER on GPUs. 1. Generalized Born. J. Chem. Theory Comput. 8, 1542-1555 (2012).
4. R. Salomon-Ferrer, A. W. Gotz, D. Poole, S. Le Grand, R. C. Walker, Routine Microsecond Molecular Dynamics Simulations with AMBER on GPUs. 2. Explicit Solvent Particle Mesh Ewald. J. Chem. Theory Comput. 9, 3878-3888 (2013).
5. D. R. Roe, T. E. Cheatham, PTRAJ and CPPTRAJ: Software for Processing and Analysis of Molecular Dynamics Trajectory Data. J. Chem. Theory Comput. 9, 3084-3095 (2013).
6. Anonymous (2012) Molecular Operating Environment (MOE) (Chemical Computing Group Inc., Montreal).
7. E. Vanquelef et al., R.E.D. Server: a web service for deriving RESP and ESP charges and building force field libraries for new molecules and molecular fragments. Nucleic Acids Res. 39, W511-517 (2011).
8. J. Wang, W. Wang, P. A. Kollman, D. A. Case, Antechamber, an accessory software package for molecular mechanical calculations. J. Chem. Inf. Comput. Sci., 1-41 (2001).
9. B. R. Miller et al., MMPBSA.py: An Efficient Program for End-State Free Energy Calculations. J. Chem. Theory Comput. 8, 3314-3321 (2012).
10. Z. Xu et al., Zn2+-Triggered Amide Tautomerization Produces a Highly Zn2+-Selective, Cell-Permeable, and Ratiometric Fluorescent Sensor. J. Am. Chem. Soc. 132, 601-610 (2010).
11. N. G. Anthony et al., Antimicrobial Lexitropsins Containing Amide, Amidine, and Alkene Linking Groups. J. Med. Chem. 50, 6116-6125 (2007).
12. A. Trösch, H. Vahrenkamp, Zinc Complexes of Tripodal N3O Ligands. Eur. J. Inorg. Chem. 1998, 827-832 (1998).
13. E. N. Durantini, Synthesis of functonal olefins using the Wittig-Horner reaction in different media. Synth. Commun. 29, 4201-4222 (1999).
14. B. D. Wall et al., Variation of Formal Hydrogen-Bonding Networks within Electronically Delocalized π-Conjugated Oligopeptide Nanostructures. Langmuir 30, 11375-11385 (2014).
